# In Silico Identification of MYB and bHLH Families Reveals Candidate Transcription Factors for Secondary Metabolic Pathways in *Cannabis sativa* L.

**DOI:** 10.3390/plants9111540

**Published:** 2020-11-11

**Authors:** Laura Bassolino, Matteo Buti, Flavia Fulvio, Alessandro Pennesi, Giuseppe Mandolino, Justyna Milc, Enrico Francia, Roberta Paris

**Affiliations:** 1 CREA-Research Centre for Cereal and Industrial Crops, 40128 Bologna, Italy; flavia.fulvio@crea.gov.it (F.F.); alessandro.pennesi@studenti.unipr.it (A.P.); giuseppe.mandolino@crea.gov.it (G.M.); 2 Department of Agriculture, Food, Environment and Forestry, University of Florence, 50144 Firenze, Italy; matteo.buti@unifi.it; 3 Department of Life Sciences, Centre BIOGEST-SITEIA, University of Modena and Reggio Emilia, 42122 Reggio Emilia, Italy; justynaanna.milc@unimore.it (J.M.); enrico.francia@unimore.it (E.F.)

**Keywords:** *Cannabis sativa*, genome-wide analysis, MYBs, bHLH, flavonoids

## Abstract

Plant secondary metabolic pathways are finely regulated by the activity of transcription factors, among which members of the bHLH and MYB subfamilies play a main role. *Cannabis sativa* L. is a unique officinal plant species with over 600 synthesized phytochemicals having diverse scale-up industrial and pharmaceutical usage. Despite comprehensive knowledge of cannabinoids’ metabolic pathways, very little is known about their regulation, while the literature on flavonoids’ metabolic pathways is still scarce. In this study, we provide the first genome-wide analysis of bHLH and MYB families in *C. sativa* reference cultivar CBDRx and identification of candidate coding sequences for these transcription factors. *Cannabis sativa* bHLHs and MYBs were then classified into functional subfamilies through comparative phylogenetic analysis with *A. thaliana* transcription factors. Analyses of gene structure and motif distribution confirmed that CsbHLHs and CsMYBs belonging to the same evolutionary clade share common features at both gene and amino acidic level. Candidate regulatory genes for key metabolic pathways leading to flavonoid and cannabinoid synthesis in *Cannabis* were also retrieved. Furthermore, a candidate gene approach was used to identify structural enzyme-coding genes for flavonoid and cannabinoid synthesis. Taken as a whole, this work represents a valuable resource of candidate genes for further investigation of the *C. sativa* cannabinoid and flavonoid metabolic pathways for genomic studies and breeding programs.

## 1. Introduction

Accumulation of plant secondary metabolites is considered a response to biotic and abiotic cues; thus, their energy-consuming synthesis needs to be fine-tuned and tightly regulated in time (e.g., developmental stages) and space (e.g., tissues and organs). Transcription factors (TFs) are essential as players of transcriptional regulation, as they act on target genes’ cis-regulatory promoters to modulate their expression, thereby controlling several aspects of plant growth, development, and adaptation to the environment. Despite the variability in TF numbers among plant species, a common trend was observed: the larger the genome size, the higher the number of TFs [1,2]. According to the DNA binding domain, plant TFs are grouped into different families, with 58 groups identified to date; myeloblastosis (MYB) and basic helix-loop-helix (bHLH) superfamilies were found in all eukaryotes and are enormously expanded in plants, playing key roles in the regulation of several cellular processes, responses to stresses, and primary and secondary metabolism [3]. MYB proteins share a highly conserved domain at the N-terminal involved in DNA binding and a more variable C-terminal region. The DNA binding region of the MYB domain consists of one to four imperfect R repeats, each composed of approximately 50–52 aminoacidic residues forming three helices; the second and third helices constitute a helix-turn-helix (HTH) fold with three regularly interspaced tryptophan residues interacting with the DNA major groove. According to the MYB domain number, MYBs are sub-grouped into R3-MYB, R2R3-MYB, and R1R2R3-MYB harboring one, two, and three repeats, respectively. Furthermore, the R4-MYB sub-group is characterized by atypical R1/R2 repeats [4,5,6]. 

Basic helix-loop-helix (bHLH) transcription factors modulate plant secondary metabolite pathways, epidermal differentiation (e.g., trichomes), and responses to biotic and abiotic stresses [7,8]. These proteins are defined by a conserved domain of approximately 50–60 amino acids consisting of two functional regions: (i) a 15-aminoacidic long basic region at the N-terminus, involved in DNA binding and enriched in basic residues; (ii) a dimerization domain (HLH) at the C-terminal region, consisting of a hydrophobic core that forms two α-helices separated by a loop region of variable length. Detailed reviews on MYB and bHLH TFs in plants are available [4,5,6,9].

*Cannabis* (*Cannabis sativa* L.) is a species belonging to the *Cannabaceae* family, which also includes *Humulus* L. It is an herbaceous annual plant native to Central Asia and is currently cultivated worldwide in diversified pedo-climatic conditions. It is a dioecious plant; however, due to breeding and selection processes, monoecious varieties with male and female separate flowers on the same individual have also been developed [10]. *Cannabis* is considered a versatile multipurpose crop due to the diverse usages in both pharmaceutical and industrial chains of *Cannabis*-derived products (e.g., fibers, phytochemicals, and oil), which resemble the multifaceted use of plant tissues (stems, inflorescence, seeds) and that are well suited for a circular bio-economy scenario. Its female inflorescence is a unique bio-factory where over 600 phytochemicals are synthesized, although the number of newly isolated metabolites is constantly growing [11,12,13,14] Phytocannabinoids—a class of terpenophenolics—and terpenoids—lipophilic compounds with diverse repeats of isoprene units—are the most important *Cannabis*-derived secondary metabolites, accumulating in specialized epidermal glands known as trichomes. Cannabinoids and terpenoids are both considered chemotaxonomic markers and valuable compounds of medicinal interest [15]. 

Flavonoids are the largest class of polyphenolic compounds, with more than 8000 metabolites identified to date. These molecules share a general C3-C6-C3 diphenylpropane structure with two aromatic rings linked by a three-carbon bridge and have diverse roles in biological and eco-physiological processes. More than 20 flavonoids belonging to flavone and flavonol subclasses (mainly the O-glycosylated forms of diverse aglycones like apigenin, leuteolin, quercetin, kaempferol) have been identified in *Cannabis* [16]. Interestingly, *Cannabis* synthesizes unique methylated isoprenoid flavones, namely cannflavin A, cannflavin B, and cannflavin C, whose anti-inflammatory properties have been demonstrated [17,18]. Despite the efforts in understanding the molecular basis of cannabinoid and terpenoid synthesis driven by medical, recreational, and industrial end uses of *Cannabis* inflorescences, very few reports are available on flavonoid synthesis in *Cannabis* [11,16,19].

*Cannabis* is a diploid species (n = 10) with nine autosomes and a pair of sex chromosomes and an estimated haploid genome size of 818 and 843 mega base pairs (Mbp) for female and male plants, respectively [20]. The species is characterized by high heterozygosity due to the wind pollination, large effective population size, and recent hybridization with divergent lineage. Twelve *Cannabis* genome assemblies are available to date, including drug-type (Purple Kush, PK) and industrial-type (e.g., Finola) cultivars [21,22,23]. However, the quality of genome resources is affected by diverse sequencing platforms and coverage. Indeed, draft assemblies with chromosome pseudomolecule sequences are available only for PK, Finola, CBDRx, and a wild *Cannabis* line [24] while, in the other cultivars, assemblies are still at the unmapped scaffold stage. Due to the newly released high-quality gene annotation [25], the genome assembly, namely cs10 of a high cannabidiolic acid (CBDA) cultivar of *C. sativa* (CBDRx) [23,26,27], has been chosen as reference genome by the *Cannabis* scientific community and is publicly available on the Genome Data Viewer (GDV) platform, provided by the National Center for Biotechnology Information (NCBI) website. The availability of chromosome-level cs10 genome assembly and genome-wide annotations, together with abundant transcriptomic data and recently released long read-sequencing [28], will further facilitate in silico analyses on target gene loci affecting key phenotypic traits of *Cannabis* and will speed up the improvement of genetic and genomic knowledge on this species.

The present study identifies TFs belonging to *Cannabis* MYB and bHLH families by exploiting the cs10 genome assembly using comprehensive genome-wide analysis and bioinformatics approaches. Furthermore, mapping of genomic loci involved in flavonoid and phytocannabinoid synthesis on cs10 assembly is reported. To the best of our knowledge, such a study has not yet been reported to date for this species. Therefore, even if the work was entirely performed in silico and does not contain any experimental evidence, we are confident that our data on the organization, distribution, and evolution of MYBs and bHLHs will provide a useful baseline resource for both plant scientists and breeders aiming at genetic improvement for specific metabolic traits via conventional breeding and/or metabolic engineering.

## 2. Results and Discussion

### 2.1. Identification of Cannabis MYB and bHLH Genes

To identify candidate regulatory TFs belonging to bHLH and MYB superfamilies, a BLASTP search was done using the HMM profile of bHLHs (PF00010) and MYBs (PF00249) as queries and the genome assembly cs10 [29] as subject, as described in the Materials and Methods section of this paper. Retrieved sequences were double-checked using the CD Database (which allows scoring proteins based on conserved domains querying different databases) and ScanProsite (Supplementary Dataset S1–S3). A total of 121 bHLH and 104 MYB protein sequences were identified. Searching for genomic loci, 89 genes encoding for *Cannabis* bHLHs, and 94 genes encoding for *Cannabis* MYBs were found, suggesting that isoforms are present in the amino acid sequence dataset of both families. For simplicity, all sequences were renamed as CsMYBX and CsbHLHX, where X is a progressive number; all sequences are listed in Supplementary Dataset S1 and S2 and Supplementary Tables S1 and S2. 

Based on the number of R repeats in the N-terminal domain, MYB proteins were classified into three subclasses: 10 belonging to R3-MYBs, 6 to R1R2R3-MYBs, and 88 to R2R3-MYBs; these MYBs are encoded by 6, 4, and 79 genomic loci, respectively. The R2R3-MYB is the largest MYB class in *Cannabis*, according to the findings in other species [4]. We also checked the protein subcellular localization using the PSORT software and, as expected, according to their transcription factor nature, the probability of nuclear localization of these proteins is 95% and 94% for bHLHs and MYBs, respectively (Supplementary Tables S1 and S2).

### 2.2. Phylogenetic Analysis, Gene Structures, and Motif Composition of bHLH Genes in Cannabis

**Figure 1:**
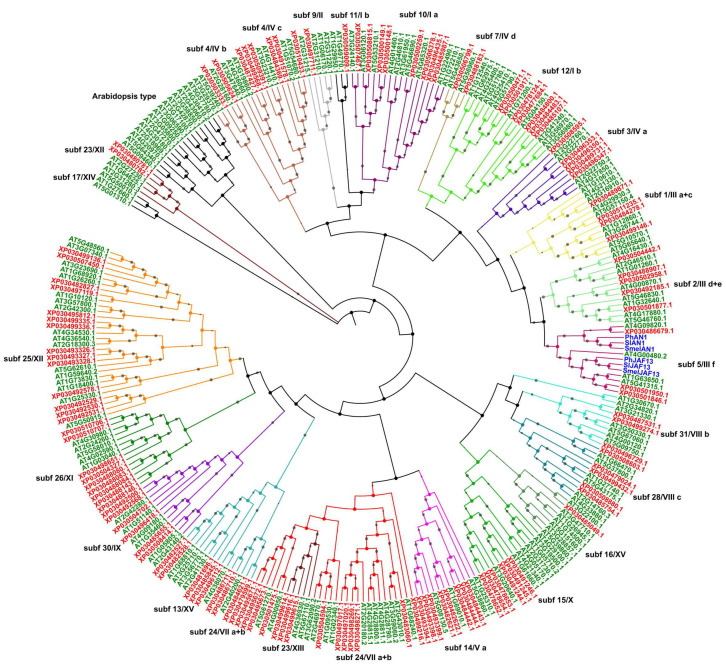
Comparative phylogenetic tree and classification of bHLH proteins in *Cannabis sativa* and *Arabidopsis thaliana*. The optimal tree (sum of branch length = 74.12972819) is shown. The tree is drawn to scale, with branches’ lengths in the same units as those of the evolutionary distances used to infer the phylogenetic tree. The percentage of replicate trees in which the associated taxa clustered together in the bootstrap test (1000 replicates) is displayed next to the branches as gray circle with size ranging from 1 to 5. Branches corresponding to partitions reproduced in less than 50% bootstrap replicates are collapsed. The genome-wide analysis involved 294 amino acid sequences divided into 26 phylogenetic subgroups marked with different clade colors and numbers corresponding to known subfamilies [30,31]. The leaf labels of bHLH members from *Arabidopsis*, *Cannabis*, and other species are written in green, red, and blue, respectively.

To gain insights into the evolutionary relationships between *Cannabis* bHLH proteins, to classify them into subfamilies, and thus to identify candidate genes for target traits, the *Arabidopsis* bHLHs were used as reference, and a comparative phylogenetic analysis between the two dicots was performed in MEGA X. The resulting Neighbor-Joining tree was built using a sequence dataset including 121 CsbHLH *Cannabis* proteins, 167 AtbHLH *Arabidopsis* proteins selected according to Carretero-Paulet et al. [30], and 5 proteins known to be involved in anthocyanin regulation in *Solanaceae* (File S1). The overall bHLH set was clustered into twenty-five clades or evolutionary lineages corresponding to distinct functional subfamilies based on tree topology and on previously reported *Arabidopsis* classification [30,31] (Figure 1). This distribution is consistent with earlier reports in other species, indicating 15 to 25 bHLH subfamilies [31,32,33] and suggesting a conserved role in plant development. In this work, we divide and discuss *Cannabis* subfamilies according to the most recent classification [31], although the previous one [30] is also reported in Figure 1 for complete information. *Cannabis* proteins were distributed in all subfamilies, with exception of the *Arabidopsis*-specific clade, and the numbers in each clade varied from 1 (subfamily 9) to 14, with subfamily 25 being the largest group (Supplementary Table S1). Members within the same subfamily are likely involved in the same pathways [34], and the distribution of *Cannabis* bHLH proteins within distinct subfamilies provides evidence of conserved molecular function with respect to members from other species. Interestingly, the Pfam analysis identified the conserved PF14215.6 signature for CsbHLH111–121, suggesting that these bHLHs might be involved in the regulation of phenylpropanoid synthesis. We identified three *Cannabis* proteins, namely CsbHLH112, CsbHLH113, and CsbHLH114, showing high homology with known flavonoid-related bHLHs both in *Solanaceae* and in *Arabidopsis* within subfamily 5/III f. Indeed, members clustering in this clade have been shown to regulate anthocyanin synthesis by acting as components of MYB-bHLH-WD40 regulatory complex on the promoter sequences of structural genes. Phylogenetic results indicate that *Cannabis* bHLHs belonging to subfamily 5 diverged in two distinct subclades, both involved in anthocyanin regulation: CsbHLH112 is the orthologous of AtTT8 (AT4G09820.1) and, together with AN1 (petunia, eggplant and tomato), is grouped in the AN1 subclade, while the two paralogs CsbHLH113 and CsbHLH114 fall into the JAF13 subclade including JAF13 (*Solanaceae* proteins) and two *Arabidopsis* paralogs, GL3 (AT5G41315.1) and EGL3 (AT1G63650.1), involved in trichome patterning. This pattern is common to other species where anthocyanin regulation has been investigated [35,36,37,38], thus suggesting that these three bHLH-encoding genes are strong candidates as anthocyanin regulators in *Cannabis*, although their function needs to be further explored.

Three *Arabidopsis* bHLH proteins, FAMA (FMA), SPEECHLESS (SPCH), and MUTE encoded by AT3G24140, AT5G53210, and AT3G06120 genes, respectively, are members of subfamily 10 and act in controlling stomata differentiation and development affecting plant performance under diverse environmental conditions [39,40]. We found the corresponding *Cannabis* orthologs CsSPCH (CsbHLH61 and CsbHLH62 isoforms encoded by LOC115715630 gene), CsFMA (CsbHLH59 encoded by LOC115722405), and CsMUTE (CsbHLH60 encoded by LOC115722405) in subfamily 10, suggesting that, due to functional conservation of transcription factors within each subfamily, they might be involved in stomatal development also in *Cannabis*, according to previous reports in tomato and peach [41]. Knowledge of genes involved in stomatal development in dicots is limited to *Arabidopsis* and tomato; thus, functional characterization of these genes in *Cannabis* would contribute to shedding light on underlying molecular mechanisms.

*C. sativa* is a facultative short-day plant, but photoperiod-independent genotypes (autoflowering) also exist; however, the molecular mechanisms underlying flowering time regulation in *Cannabis* are still unknown. Interestingly, we identified *Cannabis* bHLH orthologs of *Arabidopsis* genes within subfamilies 2, 24, and 28 whose members are known to be involved in flower development and light signaling perception, including CsbHLH27 (ortholog of AtHEC3) and CsbHLH27 (ortholog of AtHEC1), CsbHLH proteins of the PIF subfamily (CsbHLH30/PIF3, CsbHLH41/PIF7, CsbHLH33/PIF1, and bHLH31/PIF5), CsbHLH29 (ortholog of AtUNE10), and CsSPATULA (CsbHLH25). Moreover, we found five CsbHLHs (from bHLH115 to bHLH119) among members of subfamily 2/III d + e, known to include genes involved in response to several biotic and abiotic stimuli as well as anthocyanin metabolism and hormone (jasmonic and abscisic acid) signaling [28]. *Cannabis* orthologs of AtMYC2 (CsbHLH117-CsbHLH119) and AtMYC3 (CsbHLH118) were identified, suggesting a potential role in jasmonic acid-dependent flowering time regulation.

**Figure 2:**
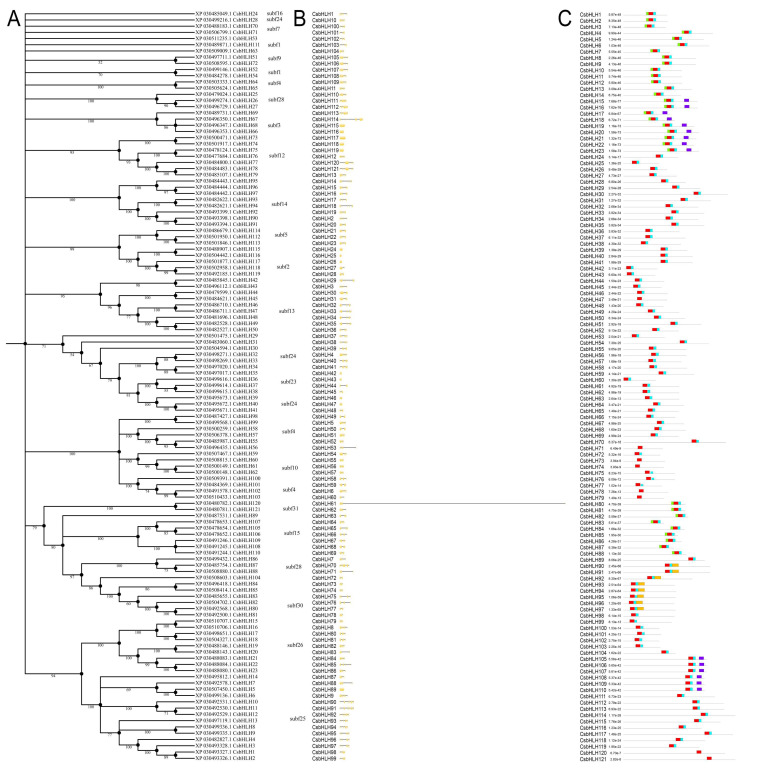
Phylogenetic analysis, gene structure, and motif distribution of *Cannabis* bHLH subfamilies. (**A**) Phylogenetic analysis of CsbHLHs. Amino acid sequences of the 121 CsbHLHs were used to construct the NJ tree using MEGA X with 1000 bootstrap replicates. Bootstrap values > 50 are shown on the branches. (**B**) Location and length of exons and introns of *Cannabis* bHLH family genes. Exons and introns are presented as filled yellow sticks and thin gray single lines, respectively. (**C**) MEME analysis of CsbHLHs motifs. Five kinds of colored blocks correspond to five different motifs in bHLH proteins. The length of gray line indicates the length of a sequence with respect to the total sequences dataset, while the position of each block represents for the location of a motif within a target sequence. Details of motifs consensus sequences are listed in Supplementary File S4.

We identified *Cannabis* bHLH clustering in brassinosteroids and metal homeostasis-related subfamilies: BEE (CsbHLH7/BEE3) in Subf 25, BIM (CsbHLH91 and isoforms/BIM1, CsbHLH94 and isoforms/BIM2) in Subf 14 and CsbHLH103, the ortholog of AtILR3 falling into subfamily 4. Phylogenetic analysis was performed by considering the 121 *Cannabis* amino acid sequences (Figure 2A) to address member composition within each subfamily; as expected, *Cannabis* proteins were distributed in each clade with a slightly different order, but with the same pattern as in the comparative phylogenetic tree.

Gene structure analysis of *CsbHLHs* was performed by submitting both genomic sequences and CDSs to the Gene Structure Display Server (GSDS) web-tool. The number of introns ranged from 0 to 9 with various lengths: 9 genes were with a single exon, 11 with one intron, and the remaining genes shared an average of 5 introns (Figure 2B). Moreover, to investigate whether *Cannabis* bHLH proteins clustered in the same or adjacent clades also sharing a similar pattern of conserved motifs, their distribution and identity were analyzed by using MEME suite. The search was limited to five motifs including the HLH domain. All CsbHLH proteins shared the highly conserved motif 1 (red blocks) including the basic region, first helix, and variable loop region of the N-terminal domain involved in DNA binding (Figure 2C, Supplementary File S4). Motif 2 (blue blocks), shared by 113 proteins, was composed only by the second helix; this pattern is *Cannabis*-specific since in other species, motif 2 includes both loop region and helix 2 [32,41,42]. The gene *LOC115703614* encodes for two different isoforms: CsbHLH46 and 47. Interestingly, the CsbHLH46 has both the conserved motifs 1 and 2, while CsbHLH47 lacks motif 2 corresponding to the DNA binding domain, thus suggesting that it might be a not functional isoform. Additionally, CsbHLH71, CsbHLH73, CsbHLH74, CsbHLH78, and CsbHLH79, clustered in subfamily 12, and CsbHLH120/121 isoforms are atypical bHLH proteins with only basic region and helix 1.

**Figure 3:**
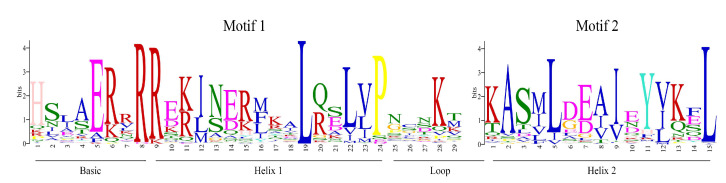
Logo consensus sequences of bHLH subfamily from *Cannabis.* Logo sequences of motif 1 and 2 represent the bHLH domain in *Cannabis*. The overall height of each stack indicates the conservation of the sequence at a certain position. Capital letters indicate >50% conservation of amino acids within the domains. The width of motifs is indicated by Arabic number under the colored letters, where each color represents a type of amino acid. Full sequences are listed in Supplementary Files S1.

Logos of motif 1 and motif 2 are shown in Figure 3. According to other species [43], the N-terminal DNA binding and protein dimerization domain is composed of a stretch of 10 basic amino acid residues (the basic region) characterized by the conserved glutamic acid (E) residue at position 5 and a portion of approximately 40 (29 and 15 amino acids of motif 1 and 2, respectively) conserved amino acids which constitutes the helix 1-loop-helix 2 region (the HLH motif). The regions of both helices are characterized by conserved hydrophobic amino acids like isoleucine (I), leucine (L), and valine (V). The loop, which starts after a conserved P residue that breaks the two helices, is generally of variable length and in *Cannabis* is 5 amino acids long with a conserved K residue, similar to most plant bHLHs [31].

Apart from the bHLH domain, CsbHLH proteins shared conserved non-bHLH motifs like motif 3 (green blocks) that were present in 32 *Cannabis* proteins clustering in adjacent subfamilies (CsbHLH1-23 in Subf 25–26 and CsbHLH80-88 in Subf 28–30). The CsbHLH15-23 and CsbHLH105-110 (subfamily 15) proteins shared motif 4 (dark blue blocks) while motif 5 (orange blocks) is shared only by eight members of BIM-type bHLHs falling into subfamily 14.

### 2.3. Phylogenetic Analysis, Gene Structure, and Motif Composition of MYB Genes in Cannabis 

**Figure 4:**
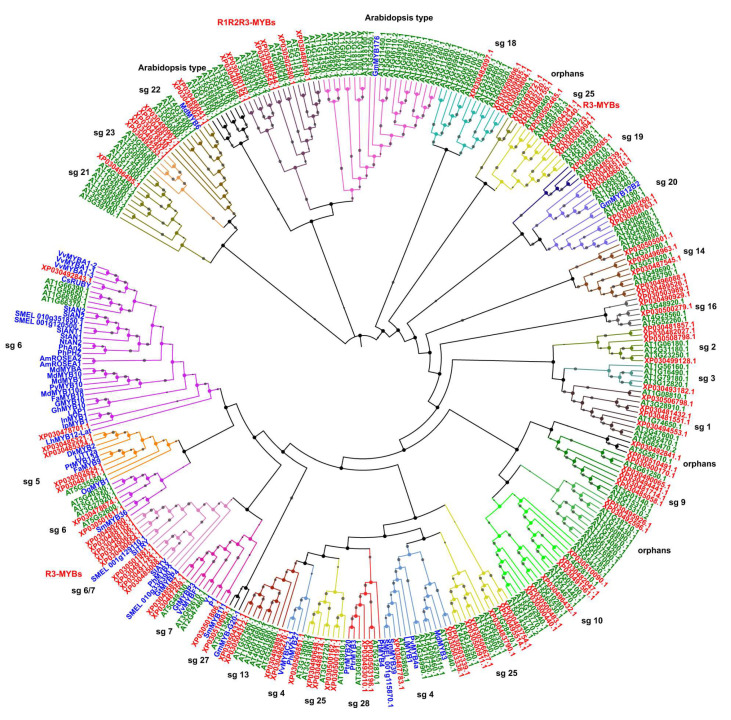
Comparative phylogenetic tree and classification of MYB proteins in *Cannabis sativa* and *Arabidopsis thaliana*. The optimal tree (sum of branch length = 69.59273343) is shown. The tree is drawn to scale, with branch lengths in the same units as those of the evolutionary distances used to infer the phylogenetic tree. The percentage of replicate trees in which the associated taxa clustered together in the bootstrap test (1000 replicates) is displayed next to the branches as gray circle with size ranging from 1 to 5. Branches corresponding to partitions reproduced in less than 50% bootstrap replicates are collapsed. The genome-wide analysis involved 330 amino acid sequences divided into 30 phylogenetic subgroups marked with different clade colors and numbers corresponding to known subfamilies. The leaf labels of MYB members from *Arabidopsis*, *Cannabis*, and other species are written in green, red, and blue, respectively.

A similar approach was applied to classify *Cannabis* MYBs based on *A. thaliana* reference proteome and using the already published information [6]. To identify flavonoid-related MYB proteins, 58 amino acid sequences known to regulate flavonoids in other species and clustered in subgroups 4 to 7 were included in the analysis (File S2). *Cannabis* MYBs clustered in 27 distinct phylogenetic groups (subgroups 1–27) including the orphan groups (Figure 4). Besides this, two *Arabidopsis*-specific subgroups were identified, with no *Cannabis* sequences included. Based on phylogenetic tree results, we identified a set of candidate genes for the regulation of flavonoid metabolism, which clustered in subgroup 6, like XP030492843.1 (CsMYB82), the ortholog of CsRUBY, and XP030478701.1 (CsMYB87), the ortholog of *InMYB1* gene. Both these MYBs are positive inducers of anthocyanin pigmentation in *Citrus* and *Ipomea* [44,45], respectively. In a separate subclade of subgroup 6, we also found XP030478674.1 (CsMYB45), corresponding to the AtMYB82 from *Arabidopsis*, known to be involved in trichome differentiation and anthocyanin metabolism [46], XP030501617.1 (CsMYB39) ortholog of *SmMYB36* gene and master regulator of anthocyanin synthesis in sage (*Salvia officinalis* L.) [47]. Notably, this analysis enables the identification of different types of anthocyanin-related MYBs which might have a role as negative regulators. Indeed, members falling into subgroup 4 are repressors of flavonoid biosynthesis, and we identified four CsMYBs belonging to this subgroup; among them, XP030496270.1 (CsMYB59) clustered with PhMYB27 and VvMYBC2-L1, well-known repressors of anthocyanin and tannin synthesis in petunia and grape, respectively [48]. Furthermore, all CsR3-MYBs, except for CsMYB1 and MYB2 isoforms, fall into a subgroup that spanned from subgroup 6 and 7 and included several known R3-type repressors for anthocyanins [35,49,50], suggesting that these R3-MYBs might be involved in regulating anthocyanin synthesis in *Cannabis* by acting as repressors. Regarding proanthocyanidins/tannins (subgroup 5), we found two *Cannabis* proteins, XP030487751.1 and XP030502824.1, that might be related to this branch of the phenylpropanoid pathway.

Only a few studies focused on the identification of regulatory elements in *C. sativa* involved in the cannabinoid biosynthetic pathway. Marks and coworkers [51] found two candidate MYB-encoding genes, namely CAN-UG-00833 and CAN-UG-00738, orthologs of *Arabidopsis* genes *MYB78* and *MYB12*, respectively, and differentially expressed in *Cannabis* trichomes compared to leaves. Interestingly, we found the corresponding *Cannabis* orthologs in subfamilies 20 and 7, respectively. Members of subfamily 20 are involved in biotic and abiotic stress response [5] and two candidate *Cannabis* proteins were identified, CsMYB77 (XP030482260.1) and CsMYB94 (XP030508763.1), which clustered with ATG5G49620.1 (AtMYB78). Proteins that fall into subfamily 7 are mainly involved in secondary metabolism (e.g., flavonols) and we found two *Cannabis* MYBs, CsMYB37 (XP030487259.1) and CsMYB53 (XP030485857.1), with their corresponding proteins from *Arabidopsis*, including AT247460.1 (MYB12), known to be involved in flavonol regulation in *Arabidopsis*, tomato, and eggplant [52,53]. To summarize, these CsMYBs are interesting candidates for both cannabinoid and flavonol regulation, and their functional role needs to be further elucidated.

Trichomes are key specialized glands distributed on leaf epidermis, and they represent the cannabinoid and terpenoid accumulation site; therefore, understanding how their differentiation and development are regulated is a target both for fundamental research studies and breeding activities, aiming at enhancing secondary metabolite accumulation under diverse conditions [54]. The R1R2R3-MYBs clustered all together into the same subgroup with *Arabidopsis* members of MYB3R gene family involved in cell cycle regulation/cell division, suggesting that they might have a similar role in *Cannabis* [55]. In terms of phenylpropanoids, interestingly, we also found orthologs of known monolignol- and phenolic acid-related MYBs. Some examples are proteins in subgroup 13 (XP030495893.1/CsMYB62 and XP030504372.1/CsMYB96 and the associated isoform) and subgroup 4 (XP030503339.1/CsMYB21 and the associated isoform, XP030487783.1/CsMYB48, XP030506851.1/CsMYB57, and XP030496270.1/CsMYB59).

**Figure 5:**
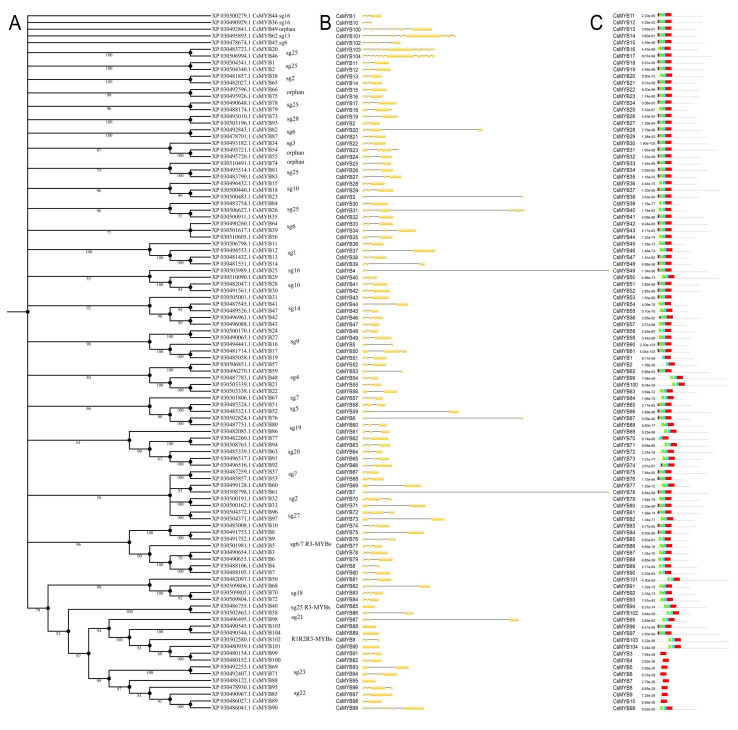
Phylogenetic analysis, gene structure, and motif distribution of *Cannabis* MYBs. (**A**) Phylogenetic analysis of CsMYBs. The amino acid sequences of the 104 CsMYBs were used to construct the NJ tree using MEGA X with 1000 bootstrap replicates. Bootstrap values > 50 are shown on the branches. (**B**) The locations and lengths of exons and introns of *Cannabis* MYB family genes. The exons and introns are presented as filled yellow sticks and thin gray single lines, respectively. (**C**) Motifs of CsMYBs were analyzed using MEME suite. Five kinds of colored blocks correspond to five different motifs in MYB proteins. The length of gray line indicates the length of a sequence with respect to the total dataset, while the position of each block denotes the locations of a motif within a target sequence. Details of motifs’ consensus sequences are listed in Supplementary File S4.

Gene structure organization of *CsMYB*s was analyzed with the same approach described for the bHLH gene family. Introns range from 0 up to 10; however, the average number for R2R3-MYBs is 2 (Figure 5B). On the contrary, the R1R2R3-MYBs share a common structure with the highest number of introns (10), which agrees with corresponding genes in peach [41].

CsMYBs clustered in subgroup 22 (CsMYB85, CsMYB88-90, CsMYB95), encompassing ABA inducible genes involved in lateral root formation, are characterized by only one exon-encoding protein; conversely, the R3-MYB type share a common 2-intron structure, where introns have variable length. As described for bHLHs, these data indicate that members of the same class of R type MYBs or clustering within the same phylogenetic clade share a common organization at exon-intron level.

To gain insights into the features of the R-MYB domain of the *C. sativa* sequences and study protein diversification, the MEME program was used with a similar approach applied to the bHLH gene family (Figure 5C). Among the five identified motifs, motifs 3, 5, and 2 (green, orange, and light blue blocks, respectively) together constitute the conserved N-terminus R2 repeat of DNA binding domain, while motif 1 (red blocks) encodes for the R3 repeat shared among all 104 sequences, according to the evidence that this repeat is present in all the diverse classes of CsMYBs. Apart from these highly conserved motifs, additional motif 4 (the purple block) is common to 50 sequences. The MEME analysis was repeated considering each class of CsMYBs as separate to confirm the overall data and highlight specific motif features within each class (Supplementary File S4).

Interestingly, a conserved motif structure and occurrence are shared by different classes of MYBs. In particular, the R3-MYB type (CsMYB1-10) has the same motif patterns with only the R3-motif (red block); conversely, the atypical-R3 MYBs, CsMYB1 and CsMYB2 (isoforms of *LOC115719431*), have a partial R2 repeat limited to helix 3 (light blue blocks in Figure 5C and orange blocks in Supplementary File). The R1R2R3-MYBs share a diverse pattern with motif 5 (marked with orange blocks in Supplementary File) present in both CsMYB103-104 isoforms of *LOC115706913*. 

**Figure 6:**
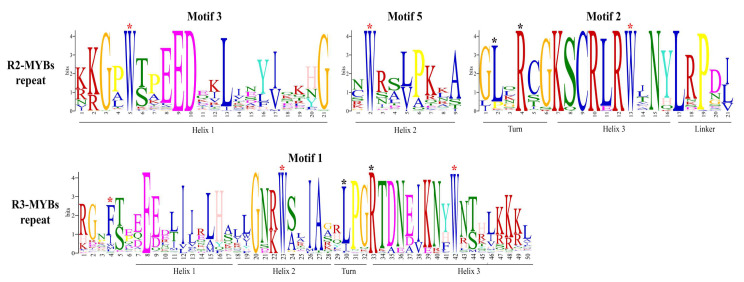
Logo consensus sequences of MYB subfamily from *Cannabis.* The logo sequences of motifs 3, 5, and 2 together constitute the R2 repeat; motif 1 corresponds to the R3 repeat of MYB domain. Red stars indicate conserved tryptophan residues, while black stars mark other conserved amino acid residues discussed in the text. The overall height of each stack indicates the conservation of the sequence at a certain position. The capital letters indicate >50% conservation of amino acids within the domains. The motif width is indicated by Arabic number under the colored letters, where each color represents a type of amino acid. Full sequences are listed in Supplementary Dataset S2.

The distribution of conserved amino acids among all *Cannabis* MYB domain sequences was in turn conserved, like observed in other species [46] (Figure 6). Each R repeat domain is characterized by regularly interspaced and conserved tryptophan (W) amino acid residues that play a key role in sequence-specific DNA binding. Furthermore, the three helices structures are maintained among all *Cannabis* MYB proteins. Indeed, in R2-repeat helix 1 (motif 3), there are 21 amino acid residues with the conserved EED stretch (two glutamic acid and one aspartic acid residues). Helix 2 (motif 5) consists of nine amino acids marked by the second conserved W residue and a turn region with characteristically conserved leucine (L) and arginine (R) residues. A third helix includes the last typical tryptophan mark. A linker region of LRPD amino acid residues is also conserved and spans from R2 to R3 repeats, as reported in tomato (*Solanum lycopersicum* L.) [56]. According to previous reports in tomato [56,57], the first tryptophan in R3 domain is replaced by phenylalanine (P), while the second and the third residues are highly shared among *Cannabis* sequences. The R3 structure is conserved among species with helix 1 containing the characteristic EEE motif (three glutamic acid amino acid residues), followed by helix 2 (sharing the 2 conserved W), a turn region similar to the R2 one and a third helix with the last conserved W residue. Moreover, the frequencies of the most prevalent amino acids at each position within each repeat were evaluated for each class of R-MYBs with WebLogo software, submitting as query the 70 deduced amino acid sequences of R1, R2, and R3 repeats (Supplementary File S4) previously aligned with ClustalW software. Analysis of R1R2R3-MYBs R2R3-MYBs and R3-MYBs, each considered as a separate class, confirmed the overall sequence data, showing conserved tryptophan and helices structure. Considering these results, we concluded that *Cannabis* MYB proteins shared a conserved domain structure compared to other species [41,47,55,56,57,58].

### 2.4. Chromosomal Distribution of Genomic Loci for CsMYBs, CsbHLHs, and Biosynthetic Enzymes for Flavonoids and Cannabinoids

**Figure 7:**
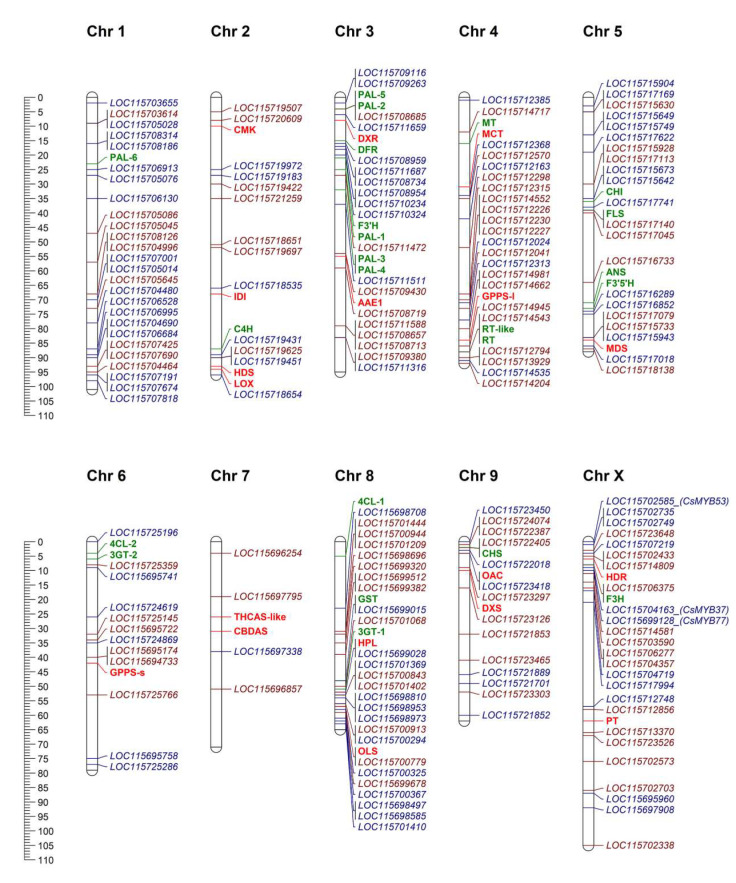
Chromosomal distribution of TFs and genes involved in flavonoid and cannabinoid pathways. Genomic loci corresponding to *CsMYB*s and *CsbHLH*s identified in this work are marked in blue and red, respectively. The position of genes encoding for flavonoid (in green) and cannabinoid (in red) enzymes is indicated by their acronym in capital letters on each chromosome. For the cannabinoid pathway, full names of represented genes are: *AAE1, acyl activating enzyme 1; CBDAS, cannabidiolic acid synthase; CMK, 4-diphosphocytidyl-2-C-methyl-D-erythritol kinase; DXR, 1-deoxy-D-xylulose 5-phosphate reductoisomerase; DXS, 1-deoxyxylulose-5-phosphate synthase; GPPS-l, GPP synthase large subunit; GPPS-s, GPP synthase small subunit; HDR, 4-hydroxy-3-methylbut-2-enyl diphosphate reductase; HDS, 4-hydroxy-3-methylbut-2-en-1-yl diphosphate synthase; HPL, hydroperoxide lyase; IDI, IPP isomerase; LOX, lipoxygenase; MCT, 4-diphosphocytidyl-methylerythritol 2-phosphate synthase; MDS, 2-C-methyl-D-erythritol 2:4-cyclodiphosphate synthase; OAC, olivetolic acid synthase; OLS, olivetol synthase; PT: prenyltransferase; THCAS-like, inactive Δ9-tetrahydrocannabinolic acid synthase.* For the flavonoid pathway, the following are represented: *PAL, phenylalanine ammonia-lyase; C4H, trans-cinnamate 4-monooxygenase; DFR, dihydroflavonol 4-reductase; F3′H, flavonoid 3′-monooxygenase; MT, flavonoid 3′5′-methyltransferase; RT, anthocyanidin-3-O-glucoside rhamnosyltransferase; RT-like, anthocyanidin-3-O-glucoside rhamnosyltransferase-like; CHI, chalcone-flavonone isomerase; FLS, flavonol synthase/flavanone 3-hydroxylase; ANS, leucoanthocyanidin dioxygenase; F3′5′H, flavonoid 3′5′-hydroxylase; 4CL2-1/2, 4-coumarate-CoA ligase 1/2; 3GT-1/2, anthocyanidin 3-O-glucoside 2″-O-glucosyltransferase 1/2; CHS, naringenin-chalcone synthase; F3H, flavanone 3-dioxygenase; F3H, flavanone 3-dioxygenase.* Positions and full names of the represented genes are listed in Supplementary Tables S3 and S4; full sequences are reported in Supplementary Dataset S5.

Chromosomal location of *CsMYB* and *CsbHLH* genes identified in the present work was retrieved in the cs10 *Cannabis* genome and displayed in Figure 7. With the only exception of chromosome 7, harboring very few loci, and the initial portion of chromosome X, the distribution of the genes involved in flavonoid and cannabinoid pathways is homogeneous without any evident physical clustering. Moreover, the structural genes encoding putative flavonoid and cannabinoid biosynthetic enzymes were identified in the cs10 assembly by performing protein-to-protein sequence searches using the BLAST tool sequences from other species [59] as queries. In total, 20 sequences related to the anthocyanin branch of the flavonoid pathway and including both early and late biosynthetic genes from Cs*PAL* (*Phenylamonia Lyase*) to Cs*GST (Glutathione S Transferase)* were mapped in the *Cannabis* genome (Supplementary Dataset S5). The retrieval of these sequences integrates a previous report [19], thus revealing the presence in the *Cannabis* genome of almost all structural genes of the biosynthetic branch of anthocyanins, although further studies are required to elucidate the details of their synthesis in *Cannabis sativa.* The same was done for enzymes involved in cannabinoid biosynthesis; a total of 18 sequences belonging to the methylerythritol phosphate pathway (MEP), hexanoate pathway, and cannabinoid pathway have been searched in the cs10 assembly using as query the *Cannabis* protein sequences listed in a recent review [27].

We did not observe a direct correlation between chromosome length and genomic loci distribution. Indeed, one of the shortest chromosomes—chr 8—is highly enriched in genomic loci for both TF families as well as structural genes. Genes encoding for inactive THCA synthase (Δ9-TetraHydroCannabinolic Acid synthase) and a CBDA synthase (CannaBidiolic Acid synthase) were located on chromosome 7, corresponding to the CBDRx chromosome previously named as 9 [29]. As expected for hemp, no functional *THCAS* was found in the cs10 assembly, while the functional CBDA gene was present. To date, little information is available on cannabinoid synthase transcriptional regulation [14,51]. A main effect Quantitative Trait Locus (QTL) for cannabinoid content (potency) was reported on chromosome 3 [29] rather than on actual chromosome 7. Interestingly, the *AAE1* gene together with several *bHLH* transcription factors were localized on this chromosome. 

We found that three out of four candidate *CsMYBs* (*MYB53, MYB37,* and *MYB77*) for the pattern of cannabinoids and flavonols are localized on chromosome X, with *MYB37* and *MYB53* also clustering in the same subfamily 20, and *MYB37* and *MYB77* are close to the *F3H*-encoding gene. The products of F3H are substrates of FLS (FLAVONOL SYNTHASE) and DFR (DIHYDROFLAVONOL REDUCTASE) enzymes leading to flavonols and anthocyanins, respectively. Moreover, two cannabinoid genes, *HDR*—a component of the MEP pathway—and *GOT*—belonging to cannabinoid-specific pathway and leading to CBGA, the precursor of all other cannabinoids—mapped on the same chromosome X, suggesting that this region might be of interest for future strategies aiming at identifying QTLs for secondary metabolite traits and/or breeding strategies. The association of the aromatic PRENYLTRANSFERASE (PT), located in chromosome X, with the total cannabinoid content and the THCA/CBDA ratio, was already suggested in a previous study [26]. The *Cannabis CsFMA (LOC115722405)* and *CsMUTE (LOC11572240)* encoding genes are located on chromosome 5 in adjacent positions (Figure 7). Adjacent genes have been already reported by other authors to cluster within the same clade [41]. Moreover, we found that some other genes co-mapping on the same chromosomes were grouped in the same subfamily. For example, CsbHLH86, CsbHLH87, and CsbHLH88 belong to subfamily 28, the members of which are required for the formation of root hairs [30,31]. Regarding the bHLH family, a total of 21 ortholog pairs between *Cannabis* and *Arabidopsis* were identified; interestingly, in some cases, bHLHs (i.e., CsbHLH19/20 (*LOC115705045*), CsbHLH21/22/23 (*LOC115704996*)), which are physically adjacent on the chromosome, were clustered in the same subfamily (Figure 1 and Figure 6). The two bHLH candidates for anthocyanin regulation in *Cannabis* previously described, CsbHLH112 and CsbHLH113, are encoded by two genomic loci (*LOC115717140* and *LOC115717045*, respectively) both mapping on chr 5 close to the *FLS* gene. Moreover, other genes encoding for flavonoid metabolism-related enzymes (*CHI*, *ANS,* and *F3′5′H*) are located on chromosome 5, suggesting that TFs mapping there might be of interest for the regulation of the flavonoid pathway. Moreover, regarding MYBs loci, members of subgroup 6 of R-type map on chr 1 (*LOC115707001*, *LOC115705014*, *LOC115707818*), suggesting that also this region might be further investigated for flavonoid traits. 

## 3. Materials and Methods 

### 3.1. Database Searches and Identification of the MYB and bHLH Genes in Cannabis sativa 

The *C. sativa* cs10 genome assembly [23,26,60], was used for genome-wide identification of ***Cannabis*** MYB and bHLH sequences. Members of both TF superfamilies were identified using a combined strategy: (i) the Hidden Markov Model (HMM) profiles for bHLH (PF00010) and MYB (PF00249) binding domain retrieved from the Pfam database [61] were used as query in tBLASTn and BLASTP search with default parameters; (ii) tomato SlAN1 and SlAN2 amino acid sequences were selected as representative of bHLH and MYB TFs, respectively, and used as query against *C. sativa* proteome with a BLASTP search. Sequences were manually checked to exclude redundancy from the final datasets. For further confirmation of sequence identity of each MYB or bHLH candidate, retrieved sequences were examined with ScanProsite tool [62] selecting “option 2”. Additionally, sequences were assessed for MYB and bHLH protein domains using the Conserved Domain Database, CDD [63]. 

Sequences without a representative domain were manually checked and excluded from the final dataset. 

### 3.2. Gene Structure Analysis

The genomic DNA and cDNA sequences of each predicted CsbHLH and CsMYB-encoding gene were manually retrieved in cs10 genome via NCBI by exploiting the Genome Data Viewer platform (GDV) and were used as aligned query for graphical display of intron and exon distribution patterns via the web-based platform Gene Structure Display Server version 2.0 (GSDS) [64,65].

### 3.3. Conserved Motif Identification and Prediction of Subcellular Localization

The motif analysis of the deduced amino acid sequences of 121 bHLHs and 104 MYBs was performed using the Multiple Em for Motif Elicitation, MEME suite [66] with the following parameters: selection of the maximum number of motifs set to X (6 to 40 width amino acid length), other parameters including e-value <1 × 10^−8^ were set to default (distribution of motif was zero or one per sequence and the motif must be present in all members within the same subfamily, e-value was set < 1 × 10^−20^). 

The distribution of amino acid residues of the R repeats motif of MYB TFs from ***Cannabis*** was obtained using the WebLogo software with default parameters [67,68] and submitting the multiple sequence alignments as queries. WoLF PSORT online software [69,70] was used to predict subcellular localization of CsMYBs and bHLHs by using plant-mode default parameters and the FASTA amino acid sequences as queries. 

### 3.4. Comparative Phylogenetic Analysis

Protein sequences of *A. thaliana* MYB and bHLH factors were downloaded from The ***Arabidopsis*** Information Resource, TAIR, database [71] using the bulk tool option. All the sequences used for the bioinformatic analyses are listed in Supplementary Files S1–S3. The MYB Dataset (S1 File) includes: 168 ***Arabidopsis*** protein sequences [4,72,73] and additional 58 amino acid sequences of MYBs from other species known to be involved in flavonoid regulation [6]. The bHLH Dataset (S2 File) includes: 167 *Arabidopsis* protein sequences and 6 amino acid sequences of bHLHs known to be involved in flavonoid regulation in several species [35,38,74]. Full length amino acid sequence alignments of identified MYBs and bHLHs from *C. sativa*, *A. thaliana,* and other species were produced via multiple sequence alignment using the ClustalW algorithm in the MEGA version X package [75]. The evolutionary history of both MYBs and bHLHs was inferred using the Neighbor-Joining method [76]. Evolutionary analyses were conducted in MEGA X according to the following parameters: all ambiguous positions were removed for each sequence pair (pairwise gap deletion option), resulting in 1368 and 1989 positions in the final datasets for bHLHs and MYBs, respectively; the statistical significance of individual nodes was assessed by bootstrap test (1000 replicates with only bootstrap values >50 displayed on the final trees) and the evolutionary distances were computed using the p-distance method, leaving related parameters as default. The NJ phylogenetic analysis involved 294 and 330 amino acid sequences for bHLHs and MYBs, respectively. 

The final figures of genome-wide NJ MYBs and bHLHs trees were obtained by using the iTOL tree editor [77,78] by using the newick extension of MEGA NJ output as query. 

The NJ phylogenetic analysis of both TFs families from ***Cannabis*** was performed as described above using the same parameters, resulting in a total of 121 and 104 amino acid sequences for bHLHs and MYBs, respectively.

### 3.5. Identification of Putative Cannabis Flavonoid and Cannabinoid Biosynthetic Genes, Chromosomal Mapping of TFs and Structural Genes on cs10 Assembly 

The putative structural enzymes involved in flavonoid biosynthetic grid were retrieved from the *C. sativa* genome assembly cs10 by using known flavonoid enzyme-encoding genes from other species (*S. melongena* and *S. lycopersicum*) as query [59] in NCBI BLASTP tool [79]. Candidate enzyme sequences were selected based on their best-hit score, and complete deduced sequences are reported in Supplementary Dataset S5. For cannabinoid biosynthetic enzyme-encoding genes, sequences were retrieved from the same assembly using previously released ***Cannabis*** sequences as query [21,27]. 

MapChart software [80] was used for the graphical representation of candidate TFs and enzyme-encoding gene positions on *C. sativa* genome cs10 assembly.

## 4. Conclusions

Despite the high conservation of flavonoid biosynthetic routes resembling the identification of both structural and regulatory enzymes in model and non-model plant species, a unique report does not exist to date on the characterization of early structural genes involved in flavonoid synthesis in *Cannabis*. This study represents the first comprehensive step in this direction. Understanding the regulatory players that control the synthesis of specific secondary metabolites, as well as the chromosomal distribution of genomic loci encoding target biosynthetic enzymes, will drive the discovery of molecular markers and QTLs for specific metabolic traits, thus boosting conventional breeding programs and genetic engineering strategies in this species. Indeed, manipulation of biosynthetic pathways leading to specific bioactive plant-derived metabolites is mainly achieved by acting on master regulatory genes like transcription factors; thus, our results might represent a useful resource to develop such an approach in *Cannabis*. 

## Supplementary Materials

The following are available online at https://www.mdpi.com/2223-7747/9/11/1540/s1, S1—Supplementary Dataset—CsbHLHs; S2—Supplementary Dataset—CsMYBs; S3—Supplementary Dataset—CDD_results; S4—Supplementary File; S5—Supplementary Dataset—Structural Genes; Supplementary Tables S1–S4: Table S1 CsbHLHs; Table S2 CsMYBs; Table S3 TFs; Table S4 Biosynthetic genes.
