# Supplementary material for: In Silico Identification of MYB and bHLH Families Reveals Candidate Transcription Factors for Secondary Metabolic Pathways in Cannabis sativa L
Source: Plants (Basel). 2020 Nov 11;9(11):1540. doi: 10.3390/plants9111540 (PMC7697600; doi:10.3390/plants9111540)
Supplement: Supplementary file 1 [file plants-09-01540-s001.zip › Bassolino et al.,SUPP.FINAL/S4-Supplementary File.docx]

Supplementary File

*In silico* identification of MYB and bHLH families reveals candidate transcription factors for secondary metabolic pathways in *Cannabis sativa* L.

Laura Bassolino^1^*, Matteo Buti^2^, Flavia Fulvio^1^, Alessandro Pennesi^1^, Giuseppe Mandolino^1^, Justyna Milc^3^, Enrico Francia^3^ and Roberta Paris^1,^*

^1^ CREA-Research Centre for Cereal and Industrial Crops Bologna, Italy; [laura.bassolino@crea.gov.it](mailto:laura.bassolino@crea.gov.it) (L.B.); [flavia.fulvio@crea.gov.it](mailto:flavia.fulvio@crea.gov.it) (F.F.); [giuseppe.mandolino@crea.gov.it](mailto:giuseppe.mandolino@crea.gov.it) (G.M.); [alessandro.pennesi@studenti.unipr.it](mailto:alessandro.pennesi@studenti.unipr.it) (A.P.)

^2^ Department of Agriculture, Food, Environment and Forestry, University of Florence, Florence, Italy; [matteo.buti@unifi.it](mailto:matteo.buti@unifi.it) (M.B.)

^3^ Department of Life Sciences, Centre BIOGEST-SITEIA, University of Modena and Reggio Emilia; [enrico.francia@unimore.it](mailto:enrico.francia@unimore.it) (E.F.); [justyna.milc@unimore.it](mailto:justyna.milc@unimore.it) (J.M.)

***** Correspondence: roberta.paris@crea.gov.it (R.P.)

**Motif site distribution analysis.** Motif site distribution analysis for both TFs families was conducted with MEME software version 5.1.1 and parameters used to run the motif analysis are here reported:

| **Motif Site Distribution** | ZOOPS: Zero or one site per sequence |
| --- | --- |
| **Objective Function** | E-value of product of p-values |
| **Starting Point Function** | E-value of product of p-values |
| **Site Strand Handling** | This alphabet only has one strand |
| **Maximum Number of Motifs** | 5 |
| **Motif E-value Threshold** | no limit |
| **Minimum Motif Width** | 2 |
| **Maximum Motif Width** | 50 |
| **Minimum Sites per Motif** | 2 |
| **Maximum Sites per Motif** | 121 for MYBs and 104 for bHLHs |

1. **Motif site distribution analysis of cannabis bHLHs**

Query: All the CsbHLHs amino acid sequences

MEME MOTIF 3 E-value 5.4e-250 Sites 32 Width 15


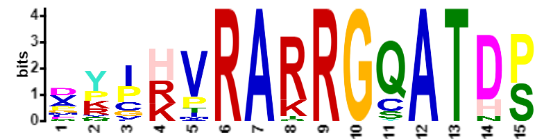


| 9. | CsbHLH9 | 251 | 3.87e-19 | **NSKASEVQKP** | **DYIHVRARRGQATDS** | **HSLAERVRRE** |
| --- | --- | --- | --- | --- | --- | --- |
| 8. | CsbHLH8 | 250 | 3.87e-19 | **NSKASEVQKP** | **DYIHVRARRGQATDS** | **HSLAERVRRE** |
| 6. | CsbHLH6 | 350 | 3.87e-19 | **NSPKPPEPPK** | **DYIHVRARRGQATDS** | **HSLAERVRRE** |
| 5. | CsbHLH5 | 315 | 3.87e-19 | **NNSKPPEPPK** | **DYIHVRARRGQATDS** | **HSLAERVRRE** |
| 3. | CsbHLH3 | 138 | 3.87e-19 | **ASIPSEPPKQ** | **DYIHVRARRGQATDS** | **HSLAERARRE** |
| 2. | CsbHLH2 | 148 | 3.87e-19 | **ASIPSEPPKQ** | **DYIHVRARRGQATDS** | **HSLAERARRE** |
| 1. | CsbHLH1 | 148 | 3.87e-19 | **ASIPSEPPKQ** | **DYIHVRARRGQATDS** | **HSLAERARRE** |
| 12. | CsbHLH12 | 180 | 2.27e-18 | **KCEEQETPPS** | **GYIHVRARRGQATDS** | **HSLAERVRRE** |
| 11. | CsbHLH11 | 179 | 2.27e-18 | **KCEEQETPPS** | **GYIHVRARRGQATDS** | **HSLAERVRRE** |
| 10. | CsbHLH10 | 176 | 2.27e-18 | **KCEEQETPPS** | **GYIHVRARRGQATDS** | **HSLAERVRRE** |
| 14. | CsbHLH14 | 197 | 2.55e-17 | **NEIREDAEKL** | **PYVHVRARRGQATDS** | **HSLAERARRE** |
| 18. | CsbHLH18 | 163 | 4.52e-17 | **QVAPAPQPPT** | **MRPRVRARRGQATDP** | **HSIAERLRRE** |
| 17. | CsbHLH17 | 133 | 4.52e-17 | **PSTVAPHPPA** | **MRPRVRARRGQATDP** | **HSIAERLRRE** |
| 16. | CsbHLH16 | 264 | 4.97e-17 | **SGSSGAAAAQ** | **PRPRVRARRGQATDP** | **HSIAERLRRE** |
| 15. | CsbHLH15 | 262 | 4.97e-17 | **SGSSGAAAAQ** | **PRPRVRARRGQATDP** | **HSIAERLRRE** |
| 4. | CsbHLH4 | 377 | 1.11e-16 | **GSQGSDPPKE** | **EYIHVRARRGQATNS** | **HSLAERVRRE** |
| 81. | CsbHLH81 | 310 | 1.97e-16 | **MEKFLHFQDA** | **VPCKVRAKRGCATHP** | **RSIAERVRRT** |
| 80. | CsbHLH80 | 309 | 1.97e-16 | **MEKFLHFQDA** | **VPCKVRAKRGCATHP** | **RSIAERVRRT** |
| 13. | CsbHLH13 | 265 | 2.72e-16 | **SSNSGDAQKD** | **NYIHVRARRGQATNS** | **HSLAERVRRE** |
| 23. | CsbHLH23 | 284 | 3.61e-16 | **TPAAVECSPT** | **AKPRPRARRGQATDP** | **HSIAERLRRE** |
| 22. | CsbHLH22 | 254 | 3.61e-16 | **TPAAVECSPT** | **AKPRPRARRGQATDP** | **HSIAERLRRE** |
| 21. | CsbHLH21 | 266 | 3.61e-16 | **TPAAVECSPT** | **AKPRPRARRGQATDP** | **HSIAERLRRE** |
| 20. | CsbHLH20 | 284 | 3.61e-16 | **TPAAVECSPT** | **AKPRPRARRGQATDP** | **HSIAERLRRE** |
| 19. | CsbHLH19 | 254 | 3.61e-16 | **TPAAVECSPT** | **AKPRPRARRGQATDP** | **HSIAERLRRE** |
| 7. | CsbHLH7 | 175 | 1.52e-15 | **NTVKVEEKLK** | **EVVHVRARRGQATDS** | **HSLAERVRRG** |
| 85. | CsbHLH85 | 268 | 1.67e-15 | **EKLLHIPEDS** | **VPCKIRAKRGCATHP** | **RSIAERERRT** |
| 82. | CsbHLH82 | 337 | 1.67e-15 | **IEKFLQFQDS** | **VPCKIRAKRGCATHP** | **RSIAERVRRT** |
| 83. | CsbHLH83 | 178 | 3.25e-15 | **TEMEKLLEDS** | **VPLRVRAKRGCATHP** | **RSIAERVRRT** |
| 84. | CsbHLH84 | 305 | 2.18e-14 | **REKVLHFQGS** | **VPCKMRAKRGYATHP** | **RSIAERNRRT** |
| 87. | CsbHLH87 | 232 | 3.74e-13 | **ENNSSSSTFD** | **SQGKTRARRGSATDP** | **QSLYARKRRE** |
| 88. | CsbHLH88 | 314 | 3.80e-12 | **HVKSLSGAVN** | **LNGKTRATRGSATDP** | **QSLYARKRRE** |
| 86. | CsbHLH86 | 274 | 9.46e-12 | **LSPKEPTALN** | **LSGKARASRGSATDP** | **QSVYARKRRE** |

MEME MOTIF 2 E-value 1.3e-767 Sites 32 Width 15


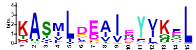


| 23. | CsbHLH23 | 329 | 2.23e-18 | **ELLPNPNKTD** | **KASMLDEIIDYVKFL** | **QLQVKVLSMS** |
| --- | --- | --- | --- | --- | --- | --- |
| 22. | CsbHLH22 | 299 | 2.23e-18 | **ELLPNPNKTD** | **KASMLDEIIDYVKFL** | **QLQVKVLSMS** |
| 21. | CsbHLH21 | 311 | 2.23e-18 | **ELLPNPNKTD** | **KASMLDEIIDYVKFL** | **QLQVKVLSMS** |
| 20. | CsbHLH20 | 329 | 2.23e-18 | **ELLPNPNKTD** | **KASMLDEIIDYVKFL** | **QLQVKVLSMS** |
| 19. | CsbHLH19 | 299 | 2.23e-18 | **ELLPNPNKTD** | **KASMLDEIIDYVKFL** | **QLQVKVLSMS** |
| 16. | CsbHLH16 | 309 | 2.23e-18 | **ELVPNANKTD** | **KASMLDEIIDYVKFL** | **QLQVKVLSMS** |
| 15. | CsbHLH15 | 307 | 2.23e-18 | **ELVPNANKTD** | **KASMLDEIIDYVKFL** | **QLQVKVLSMS** |
| 38. | CsbHLH38 | 144 | 1.91e-17 | **NLIPNSNKTD** | **KASMLDEAIEYLKQL** | **QLQVQMLAMR** |
| 37. | CsbHLH37 | 173 | 1.91e-17 | **NLIPNSNKTD** | **KASMLDEAIEYLKQL** | **QLQVQMLAMR** |
| 36. | CsbHLH36 | 173 | 1.91e-17 | **NLIPNSNKTD** | **KASMLDEAIEYLKQL** | **QLQVQMLAMR** |
| 26. | CsbHLH26 | 204 | 3.64e-17 | **RLVPGGTKMD** | **TASMLDEAIHYVKFL** | **KKQVQTLEQV** |
| 35. | CsbHLH35 | 340 | 5.75e-17 | **ELIPRCNKSD** | **KASMLDEAIEYLKSL** | **QLQVQMMSMG** |
| 34. | CsbHLH34 | 303 | 5.75e-17 | **ELIPRCNKSD** | **KASMLDEAIEYLKSL** | **QLQVQMMSMG** |
| 33. | CsbHLH33 | 340 | 5.75e-17 | **ELIPRCNKSD** | **KASMLDEAIEYLKSL** | **QLQVQMMSMG** |
| 32. | CsbHLH32 | 303 | 5.75e-17 | **ELIPRCNKSD** | **KASMLDEAIEYLKSL** | **QLQVQMMSMG** |
| 31. | CsbHLH31 | 399 | 5.75e-17 | **ELIPHSNKTD** | **KASMLDEAIEYLKSL** | **QLQLQVMWMG** |
| 29. | CsbHLH29 | 345 | 2.97e-16 | **KLVPNSSKTD** | **KASMLDEVIDYLKQL** | **QAQVQMMNRM** |
| 41. | CsbHLH41 | 271 | 4.94e-16 | **KLVPNASKTD** | **KASMLDEVIHYLKQL** | **QTQVQVMSSL** |
| 40. | CsbHLH40 | 270 | 4.94e-16 | **KLVPNASKTD** | **KASMLDEVIHYLKQL** | **QTQVQVMSSL** |
| 39. | CsbHLH39 | 271 | 4.94e-16 | **KLVPNASKTD** | **KASMLDEVIHYLKQL** | **QTQVQVMSSL** |
| 27. | CsbHLH27 | 199 | 7.01e-16 | **RLVPGGTKMD** | **TATMLDEAIRYVKFL** | **KRQIRLLQAS** |
| 30. | CsbHLH30 | 483 | 1.55e-15 | **ELIPNCNKVD** | **KASMLDEAIEYLKTL** | **QLQVQIMSMG** |
| 110. | CsbHLH110 | 430 | 2.81e-15 | **QLVSPFGKTD** | **TASVLHEAIEYIKFL** | **HDQVSVLSTP** |
| 109. | CsbHLH109 | 430 | 2.81e-15 | **QLVSPFGKTD** | **TASVLHEAIEYIKFL** | **HDQVLSTPYM** |
| 108. | CsbHLH108 | 429 | 2.81e-15 | **QLVSPFGKTD** | **TASVLHEAIEYIKFL** | **HDQVSVLSTP** |
| 114. | CsbHLH114 | 529 | 6.66e-15 | **SLVPFVTKMD** | **KASILGDTIEYVKQL** | **RKKVQDLETR** |
| 107. | CsbHLH107 | 448 | 1.01e-14 | **QLVSPFGKTD** | **TASVLSEAIEYIKFL** | **HEQVSVLSNP** |
| 106. | CsbHLH106 | 449 | 1.01e-14 | **QLVSPFGKTD** | **TASVLSEAIEYIKFL** | **HEQVSVLSNP** |
| 105. | CsbHLH105 | 449 | 1.01e-14 | **QLVSPFGKTD** | **TASVLSEAIEYIKFL** | **HEQVLSNPYM** |
| 111. | CsbHLH111 | 377 | 1.33e-14 | **SLVPKISKLD** | **RASILGDAIEYVKDL** | **QSQAKELQDE** |
| 68. | CsbHLH68 | 231 | 1.33e-14 | **AIVPGLKKMD** | **KASVLGDAIVYVKQL** | **QERVNILEEQ** |
| 13. | CsbHLH13 | 311 | 1.33e-14 | **LVPGCNKITG** | **KAVMLDEIINYVQSL** | **QQQVEFLSMK** |
| 12. | CsbHLH12 | 226 | 1.33e-14 | **LVPGCEKVTG** | **KALMLDEIINYVQSL** | **QNQVEFLSMK** |
| 11. | CsbHLH11 | 225 | 1.33e-14 | **LVPGCEKVTG** | **KALMLDEIINYVQSL** | **QNQVEFLSMK** |
| 10. | CsbHLH10 | 222 | 1.33e-14 | **LVPGCEKVTG** | **KALMLDEIINYVQSL** | **QNQVEFLSMK** |
| 6. | CsbHLH6 | 396 | 1.33e-14 | **LVPGCNKVTG** | **KALMLDEIINYVQSL** | **QRQVEFLSMK** |
| 5. | CsbHLH5 | 361 | 1.33e-14 | **LVPGCNKVTG** | **KAVMLDEIINYVQSL** | **QRQVEFLSMK** |
| 4. | CsbHLH4 | 423 | 1.33e-14 | **LVPGCSKVTG** | **KAVMLDEIINYVQSL** | **QRQVEFLSMK** |
| 18. | CsbHLH18 | 208 | 3.31e-14 | **ELVPSCNKTD** | **RAAMLDEIVDYVKFL** | **RLQVKVLSMS** |
| 17. | CsbHLH17 | 178 | 3.31e-14 | **ELVPSVNKTD** | **RAAMLDEIVDYVKFL** | **RLQVKVLSMS** |
| 92. | CsbHLH92 | 186 | 3.76e-14 | **LIPNIDQKRD** | **KASFLLEVIEYIQYL** | **QEKVHKHEGS** |
| 91. | CsbHLH91 | 300 | 3.76e-14 | **LIPNIDQKRD** | **KASFLLEVIEYIQYL** | **QEKVHKHEGS** |
| 90. | CsbHLH90 | 299 | 3.76e-14 | **LIPNIDQKRD** | **KASFLLEVIEYIQYL** | **QEKVHKHEGS** |
| 9. | CsbHLH9 | 297 | 4.26e-14 | **LVPGCNKITG** | **KAGMLDEIINYVQSL** | **QRQVEFLSMK** |
| 8. | CsbHLH8 | 296 | 4.26e-14 | **LVPGCNKITG** | **KAGMLDEIINYVQSL** | **QRQVEFLSMK** |
| 54. | CsbHLH54 | 394 | 4.84e-14 | **SVVPKISKMD** | **RASILGDAIDYLKEL** | **LQRINDLHNE** |
| 25. | CsbHLH25 | 87 | 4.84e-14 | **SLVPGGAKMD** | **TVSMLEEAIHYVKFL** | **KAQIWLHQTI** |
| 94. | CsbHLH94 | 81 | 6.19e-14 | **LVPHSDQKRD** | **TASFLLEVIEYVQFL** | **QEKVHKFEGS** |
| 93. | CsbHLH93 | 81 | 6.19e-14 | **LVPHSDQKRD** | **TASFLLEVIEYVQFL** | **QEKVHKFEGS** |
| 113. | CsbHLH113 | 477 | 7.90e-14 | **GMVPSLSKED** | **KVSILDDAIQYLKEL** | **EKRVEELESS** |
| 112. | CsbHLH112 | 475 | 7.90e-14 | **GMVPSLSKED** | **KVSILDDAIQYLKEL** | **EKRVEELESS** |
| 3. | CsbHLH3 | 185 | 1.01e-13 | **VPGCNKVVIG** | **KALVLDEIINYIQSL** | **QRQVEFLSMK** |
| 2. | CsbHLH2 | 195 | 1.01e-13 | **VPGCNKVVIG** | **KALVLDEIINYIQSL** | **QRQVEFLSMK** |
| 1. | CsbHLH1 | 194 | 1.01e-13 | **LVPGCNKVIG** | **KALVLDEIINYIQSL** | **QRQVEFLSMK** |
| 69. | CsbHLH69 | 207 | 1.13e-13 | **AIVPGLKKMD** | **KASVLGDAIKYMKQL** | **QERVKILEEE** |
| 89. | CsbHLH89 | 449 | 1.44e-13 | **KLVPGGSKMD** | **TASMLDEAANYLKFL** | **RSQVKALENL** |
| 67. | CsbHLH67 | 247 | 1.44e-13 | **AVVPGLKKMD** | **KASVLGDAIKYIKNL** | **QERLNTLEEQ** |
| 83. | CsbHLH83 | 224 | 3.59e-13 | **LVPNMDKQTN** | **TADMLEEAVEYVKCL** | **QKKIQELSEH** |
| 7. | CsbHLH7 | 221 | 4.49e-13 | **IVPGCYKTMG** | **MAVMLDEIINYVQSL** | **QNQVEFLSMK** |
| 82. | CsbHLH82 | 383 | 5.60e-13 | **LVPNMEKQTN** | **TADMLDLAVEYIKDL** | **QSQVQTLSDD** |
| 81. | CsbHLH81 | 356 | 5.60e-13 | **LVPNMDKQTN** | **TADMLDLAVEYIKDL** | **QKQFKTLSDK** |
| 80. | CsbHLH80 | 355 | 5.60e-13 | **LVPNMDKQTN** | **TADMLDLAVEYIKDL** | **QKQFKTLSDK** |
| 97. | CsbHLH97 | 75 | 6.25e-13 | **LIPQSDQKRD** | **KASFLLELIEYVQYL** | **QEKINMYEES** |
| 96. | CsbHLH96 | 68 | 6.25e-13 | **LIPQSDQKRD** | **KASFLLELIEYVQYL** | **QEKINMYEES** |
| 50. | CsbHLH50 | 228 | 6.96e-13 | **SLLPSTTKTD** | **KASLLAEVIQHVKEL** | **KRQTSLIAET** |
| 49. | CsbHLH49 | 189 | 6.96e-13 | **SLLPSTTKTD** | **KASLLAEVIQHVKEL** | **KRQTSLIAET** |
| 28. | CsbHLH28 | 274 | 7.76e-13 | **ALIPNCNKVD** | **KASILEDAIDHLKSL** | **QFQLQMMMSM** |
| 117. | CsbHLH117 | 553 | 1.07e-12 | **AVVPNVSKMD** | **KASLLGDAISYINEL** | **KSKLQTTETD** |
| 66. | CsbHLH66 | 179 | 1.07e-12 | **AVVPGLKKMD** | **KASVLGNAISYVKQL** | **EERVKTLEEE** |
| 115. | CsbHLH115 | 486 | 1.63e-12 | **AVVPNISKMD** | **KASLLGDAIAYINEL** | **QAKLKVMETE** |
| 53. | CsbHLH53 | 86 | 2.22e-12 | **AVVPKISKMD** | **KASIIKDAIEYIQKL** | **HEEERNIQAE** |
| 51. | CsbHLH51 | 334 | 2.72e-12 | **SLVPNPTKND** | **RASVVGDAIEYIREL** | **RRTVNELKIL** |
| 116. | CsbHLH116 | 387 | 4.97e-12 | **AVVPNISKMD** | **KASLLGDAITYITDL** | **QTKIRVMETE** |
| 58. | CsbHLH58 | 201 | 4.97e-12 | **MPDSYVQRGD** | **QASIVGGAIEFVKEL** | **EHLLQSLEAQ** |
| 57. | CsbHLH57 | 194 | 5.48e-12 | **MPDSYVQRGD** | **QASIIGGAINFVKEL** | **EQQVQFLGAQ** |
| 55. | CsbHLH55 | 197 | 5.48e-12 | **MPSSYVQRGD** | **QASIIGGAINFVKEL** | **EQSLQSMEGH** |
| 118. | CsbHLH118 | 368 | 6.67e-12 | **SVVPNVSKMD** | **KASLLADAVVYINEL** | **KAKVEELEAK** |
| 88. | CsbHLH88 | 359 | 7.35e-12 | **NIVPNGTKVD** | **ISTMLEEAVHYVKFL** | **QLQIKLLSSD** |
| 14. | CsbHLH14 | 243 | 7.35e-12 | **LVPGCSKISG** | **TALVLDEIINHVQSL** | **QRQVEFLSMR** |
| 87. | CsbHLH87 | 277 | 8.92e-12 | **NIVPNGTKVD** | **ISTMLEEAVQYVKFL** | **QLQIKLLSSD** |
| 86. | CsbHLH86 | 319 | 8.92e-12 | **NLVPNGTKVD** | **ISTMLEEAVQYVKFL** | **QLQIKLLSSD** |
| 42. | CsbHLH42 | 50 | 8.92e-12 | **SLLPNITKTD** | **KASLLAEVVHRVKEL** | **REKVAEVVRK** |
| 65. | CsbHLH65 | 189 | 1.08e-11 | **SLVPNITKMD** | **KASIVGDAVLYVQDL** | **QTQSKKLKAE** |
| 56. | CsbHLH56 | 212 | 1.08e-11 | **MPSSYVQRGD** | **QASIVGGAINFVKEL** | **EQLLQFLEAQ** |
| 84. | CsbHLH84 | 351 | 1.31e-11 | **LCPNMDKQTN** | **TADMLELVVDYIKDL** | **QKEVKTLSDT** |
| 45. | CsbHLH45 | 95 | 1.73e-11 | **SLTPGAKKMD** | **KASLLAEVISHLKDL** | **KRKTAETSED** |
| 64. | CsbHLH64 | 223 | 2.09e-11 | **SLVPFITKMD** | **KASIVGDAVVYLRDL** | **KKQANNLKDE** |
| 59. | CsbHLH59 | 260 | 2.29e-11 | **MPGSYVQRGD** | **QASIIGGAIEFVREL** | **EQLLQCLESQ** |
| 119. | CsbHLH119 | 325 | 2.75e-11 | **AVVPNVSRMD** | **KASLLSDAVSYINEL** | **KTKIEELESQ** |
| 44. | CsbHLH44 | 105 | 3.95e-11 | **DLVPSNEKMD** | **KATLLAEVIRQVKQL** | **KKNAAEASKG** |
| 24. | CsbHLH24 | 240 | 3.95e-11 | **KLLPWDKKMD** | **MATMLEETFKYVKFL** | **QAQVTALKAM** |
| 52. | CsbHLH52 | 217 | 4.32e-11 | **SIVPKISKMD** | **RTSILGDTIDYMKEL** | **LERISKLQEE** |
| 62. | CsbHLH62 | 157 | 4.72e-11 | **MPCFYVKRGD** | **QASIIGGVVDYITEL** | **QQVLQSLEAK** |
| 61. | CsbHLH61 | 157 | 4.72e-11 | **MPCFYVKRGD** | **QASIIGGVVDYITEL** | **QQVLQSLEAK** |
| 46. | CsbHLH46 | 120 | 4.72e-11 | **KLIPKSDKMD** | **KAALLGSVIDHVKEL** | **KRKAGEVSKY** |
| 43. | CsbHLH43 | 54 | 5.64e-11 | **TILPNLIKMD** | **KASVLAETIKRVREL** | **KKAVEEVDSV** |
| 48. | CsbHLH48 | 114 | 1.34e-10 | **SLLPCNSKTD** | **KASLLAKVVQRVKEL** | **KEQTSELTEL** |
| 60. | CsbHLH60 | 35 | 1.46e-10 | **TPCFYIKRGD** | **QASIIGGVIEFIKEM** | **HQVLQSLESK** |
| 63. | CsbHLH63 | 171 | 2.22e-10 | **LLPQLPSKAD** | **KSTIVDEAVNYIRNL** | **QQTLDKLEKQ** |
| 104. | CsbHLH104 | 276 | 2.42e-10 | **ELVPNGSKVD** | **LVTMLEKAISYVKFL** | **QLQVKVLATD** |
| 98. | CsbHLH98 | 82 | 4.64e-10 | **VLDPDRPKND** | **KATILTDTIQLLKDL** | **TSQVDKLKAE** |
| 85. | CsbHLH85 | 314 | 7.48e-10 | **LVPNMDKQTS** | **YADMLDLAVQHIKGL** | **QNQVKKLHTE** |
| 102. | CsbHLH102 | 106 | 1.10e-9 | **LDPGRPPKMD** | **KGVILGEAVRMVNQL** | **RMESQKLKQS** |
| 103. | CsbHLH103 | 112 | 1.75e-9 | **LEPGRPPKTD** | **KAAILIDAVRMVHQL** | **RGEAQKLKDS** |
| 100. | CsbHLH100 | 113 | 4.90e-9 | **LEPDRPAKTD** | **KTAILDDAVRVLNQL** | **KSENQELKET** |
| 101. | CsbHLH101 | 122 | 5.66e-9 | **MELGRLPKTD** | **KAAILSDAVRIMTQL** | **RSESRQLKQT** |
| 72. | CsbHLH72 | 105 | 6.08e-9 | **LVPGILPKAS** | **KDKVLHAATEYIKYL** | **EEKKKILEKL** |
| 70. | CsbHLH70 | 470 | 1.07e-8 | **SLLPPGTKKD** | **KASVLNSTTDYLNSL** | **KAQVEELSKR** |
| 99. | CsbHLH99 | 73 | 1.61e-8 | **TLDSDRPKND** | **KGTILTDSIQMLKDL** | **TAEVNKLKAE** |
| 95. | CsbHLH95 | 75 | 3.35e-8 | **LIPQSDQKRD** | **KASFLLEVIVCMYKA** | **YEKINMYEES** |
| 75. | CsbHLH75 | 176 | 5.63e-8 | **RLSKLKKRWT** | **APRIIDKALEYIPEL** | **ENEIEELITL** |
| 76. | CsbHLH76 | 183 | 1.52e-7 | **NSRPSKKKWT** | **APIIIDEVLEYIPEI** | **EKEIKQLTLK** |
| 77. | CsbHLH77 | 113 | 7.77e-7 | **PAADQMKKLS** | **NPSTISRTIKYIPEL** | **QNQVKGLIQK** |

MEME MOTIF 1 E-value 1.7e-1595 Sites 121 Width 29


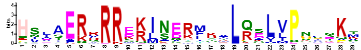


| 18. | CsbHLH18 | 178 | 1.25e-29 | **RARRGQATDP** | **HSIAERLRRERIAERMKALQELVPSCNKT** | **DRAAMLDEIV** |
| --- | --- | --- | --- | --- | --- | --- |
| 3. | CsbHLH3 | 153 | 3.87e-29 | **RARRGQATDS** | **HSLAERARREKISERMKILQDLVPGCNKV** | **VIGKALVLDE** |
| 2. | CsbHLH2 | 163 | 3.87e-29 | **RARRGQATDS** | **HSLAERARREKISERMKILQDLVPGCNKV** | **VIGKALVLDE** |
| 1. | CsbHLH1 | 163 | 3.87e-29 | **RARRGQATDS** | **HSLAERARREKISERMKILQDLVPGCNKV** | **IGKALVLDEI** |
| 16. | CsbHLH16 | 279 | 6.01e-29 | **RARRGQATDP** | **HSIAERLRRERIAERMKALQELVPNANKT** | **DKASMLDEII** |
| 15. | CsbHLH15 | 277 | 6.01e-29 | **RARRGQATDP** | **HSIAERLRRERIAERMKALQELVPNANKT** | **DKASMLDEII** |
| 5. | CsbHLH5 | 330 | 6.01e-29 | **RARRGQATDS** | **HSLAERVRREKISERMKFLQDLVPGCNKV** | **TGKAVMLDEI** |
| 6. | CsbHLH6 | 365 | 9.27e-29 | **RARRGQATDS** | **HSLAERVRREKISERMKLLQDLVPGCNKV** | **TGKALMLDEI** |
| 12. | CsbHLH12 | 195 | 1.75e-28 | **RARRGQATDS** | **HSLAERVRREKISERMKMLQKLVPGCEKV** | **TGKALMLDEI** |
| 11. | CsbHLH11 | 194 | 1.75e-28 | **RARRGQATDS** | **HSLAERVRREKISERMKMLQKLVPGCEKV** | **TGKALMLDEI** |
| 10. | CsbHLH10 | 191 | 1.75e-28 | **RARRGQATDS** | **HSLAERVRREKISERMKMLQKLVPGCEKV** | **TGKALMLDEI** |
| 9. | CsbHLH9 | 266 | 1.75e-28 | **RARRGQATDS** | **HSLAERVRREKISERMKYLQDLVPGCNKI** | **TGKAGMLDEI** |
| 8. | CsbHLH8 | 265 | 1.75e-28 | **RARRGQATDS** | **HSLAERVRREKISERMKYLQDLVPGCNKI** | **TGKAGMLDEI** |
| 4. | CsbHLH4 | 392 | 2.66e-28 | **RARRGQATNS** | **HSLAERVRREKISERMKFLQDLVPGCSKV** | **TGKAVMLDEI** |
| 30. | CsbHLH30 | 453 | 7.36e-28 | **GSKRSRAAEV** | **HNLSERRRRDRINEKMRALQELIPNCNKV** | **DKASMLDEAI** |
| 13. | CsbHLH13 | 280 | 8.98e-28 | **RARRGQATNS** | **HSLAERVRREKISERMRLLQELVPGCNKI** | **TGKAVMLDEI** |
| 7. | CsbHLH7 | 190 | 1.82e-26 | **RARRGQATDS** | **HSLAERVRRGKINERLRCLQDIVPGCYKT** | **MGMAVMLDEI** |
| 23. | CsbHLH23 | 299 | 2.17e-26 | **RARRGQATDP** | **HSIAERLRREKIAERMKGLQELLPNPNKT** | **DKASMLDEII** |
| 22. | CsbHLH22 | 269 | 2.17e-26 | **RARRGQATDP** | **HSIAERLRREKIAERMKGLQELLPNPNKT** | **DKASMLDEII** |
| 21. | CsbHLH21 | 281 | 2.17e-26 | **RARRGQATDP** | **HSIAERLRREKIAERMKGLQELLPNPNKT** | **DKASMLDEII** |
| 20. | CsbHLH20 | 299 | 2.17e-26 | **RARRGQATDP** | **HSIAERLRREKIAERMKGLQELLPNPNKT** | **DKASMLDEII** |
| 19. | CsbHLH19 | 269 | 2.17e-26 | **RARRGQATDP** | **HSIAERLRREKIAERMKGLQELLPNPNKT** | **DKASMLDEII** |
| 17. | CsbHLH17 | 148 | 4.38e-26 | **RARRGQATDP** | **HSIAERLRRERIAERIRALQELVPSVNKT** | **DRAAMLDEIV** |
| 31. | CsbHLH31 | 369 | 6.18e-26 | **SSRKSRAAEV** | **HNLSERRRRDRINEKMKALQELIPHSNKT** | **DKASMLDEAI** |
| 14. | CsbHLH14 | 212 | 7.33e-26 | **RARRGQATDS** | **HSLAERARREKINARMKLLQELVPGCSKI** | **SGTALVLDEI** |
| 35. | CsbHLH35 | 310 | 1.03e-25 | **STKRSRAAEV** | **HNLSERRRRDRINEKMKALQELIPRCNKS** | **DKASMLDEAI** |
| 34. | CsbHLH34 | 273 | 1.03e-25 | **STKRSRAAEV** | **HNLSERRRRDRINEKMKALQELIPRCNKS** | **DKASMLDEAI** |
| 33. | CsbHLH33 | 310 | 1.03e-25 | **STKRSRAAEV** | **HNLSERRRRDRINEKMKALQELIPRCNKS** | **DKASMLDEAI** |
| 32. | CsbHLH32 | 273 | 1.03e-25 | **STKRSRAAEV** | **HNLSERRRRDRINEKMKALQELIPRCNKS** | **DKASMLDEAI** |
| 116. | CsbHLH116 | 357 | 4.49e-25 | **PANGREEPLN** | **HVEAERQRREKLNQRFYALRAVVPNISKM** | **DKASLLGDAI** |
| 115. | CsbHLH115 | 456 | 4.49e-25 | **PANGREEPLN** | **HVEAERQRREKLNQRFYALRAVVPNISKM** | **DKASLLGDAI** |
| 114. | CsbHLH114 | 499 | 8.46e-25 | **GTPQDELSAN** | **HVMAERRRREKLNERFIILRSLVPFVTKM** | **DKASILGDTI** |
| 41. | CsbHLH41 | 241 | 1.35e-24 | **NSTRSRAAAI** | **HNQSERRRRDRINQKMKALQKLVPNASKT** | **DKASMLDEVI** |
| 40. | CsbHLH40 | 240 | 1.35e-24 | **NSTRSRAAAI** | **HNQSERRRRDRINQKMKALQKLVPNASKT** | **DKASMLDEVI** |
| 39. | CsbHLH39 | 241 | 1.35e-24 | **NSTRSRAAAI** | **HNQSERRRRDRINQKMKALQKLVPNASKT** | **DKASMLDEVI** |
| 117. | CsbHLH117 | 523 | 2.13e-24 | **PANGREEPLN** | **HVEAERQRREKLNQRFYALRAVVPNVSKM** | **DKASLLGDAI** |
| 81. | CsbHLH81 | 325 | 2.48e-24 | **RAKRGCATHP** | **RSIAERVRRTRISERMRKLQELVPNMDKQ** | **TNTADMLDLA** |
| 80. | CsbHLH80 | 324 | 2.48e-24 | **RAKRGCATHP** | **RSIAERVRRTRISERMRKLQELVPNMDKQ** | **TNTADMLDLA** |
| 38. | CsbHLH38 | 114 | 3.89e-24 | **SSKRSRAAEV** | **HNLSEKRRRSRINEKMKALQNLIPNSNKT** | **DKASMLDEAI** |
| 37. | CsbHLH37 | 143 | 3.89e-24 | **SSKRSRAAEV** | **HNLSEKRRRSRINEKMKALQNLIPNSNKT** | **DKASMLDEAI** |
| 36. | CsbHLH36 | 143 | 3.89e-24 | **SSKRSRAAEV** | **HNLSEKRRRSRINEKMKALQNLIPNSNKT** | **DKASMLDEAI** |
| 82. | CsbHLH82 | 352 | 1.25e-23 | **RAKRGCATHP** | **RSIAERVRRTKISERMRKLQELVPNMEKQ** | **TNTADMLDLA** |
| 29. | CsbHLH29 | 315 | 2.53e-23 | **STKRSRAAAI** | **HNQSERKRRDKINQRMTTLQKLVPNSSKT** | **DKASMLDEVI** |
| 118. | CsbHLH118 | 338 | 3.83e-23 | **TPTESQMPVN** | **HVEAERQRREKLNHRFYKLRSVVPNVSKM** | **DKASLLADAV** |
| 83. | CsbHLH83 | 193 | 4.39e-23 | **RAKRGCATHP** | **RSIAERVRRTRISDRIRKLQELVPNMDKQ** | **TNTADMLEEA** |
| 28. | CsbHLH28 | 244 | 4.39e-23 | **PLPRKRSAKL** | **HSLSERRRRDKINKRIRTLKALIPNCNKV** | **DKASILEDAI** |
| 104. | CsbHLH104 | 246 | 1.29e-22 | **VQKPCPSKDP** | **QSIAAKNRRERISERLKMLQELVPNGSKV** | **DLVTMLEKAI** |
| 44. | CsbHLH44 | 75 | 1.68e-22 | **EAKALAALKN** | **HSEAERRRRERINAHLATLRDLVPSNEKM** | **DKATLLAEVI** |
| 86. | CsbHLH86 | 289 | 3.65e-22 | **RASRGSATDP** | **QSVYARKRREKINERLKVLQNLVPNGTKV** | **DISTMLEEAV** |
| 50. | CsbHLH50 | 198 | 4.71e-22 | **DAKALAASKS** | **HSEAERRRRERINNHLAKLRSLLPSTTKT** | **DKASLLAEVI** |
| 49. | CsbHLH49 | 159 | 4.71e-22 | **DAKALAASKS** | **HSEAERRRRERINNHLAKLRSLLPSTTKT** | **DKASLLAEVI** |
| 27. | CsbHLH27 | 169 | 5.35e-22 | **RRNVRISDDP** | **QSVAARHRRERISERIRILQRLVPGGTKM** | **DTATMLDEAI** |
| 26. | CsbHLH26 | 174 | 5.35e-22 | **RRNVKISKDP** | **QSVAARHRRERISERIRILQRLVPGGTKM** | **DTASMLDEAI** |
| 94. | CsbHLH94 | 50 | 6.07e-22 | **NDKASAVRSK** | **HSVTEQRRRSKINERFQILRDLVPHSDQK** | **RDTASFLLEV** |
| 93. | CsbHLH93 | 50 | 6.07e-22 | **NDKASAVRSK** | **HSVTEQRRRSKINERFQILRDLVPHSDQK** | **RDTASFLLEV** |
| 92. | CsbHLH92 | 155 | 6.07e-22 | **DQKANTPRSK** | **HSATEQRRRSKINDRFQRLRELIPNIDQK** | **RDKASFLLEV** |
| 91. | CsbHLH91 | 269 | 6.07e-22 | **DQKANTPRSK** | **HSATEQRRRSKINDRFQRLRELIPNIDQK** | **RDKASFLLEV** |
| 90. | CsbHLH90 | 268 | 6.07e-22 | **DQKANTPRSK** | **HSATEQRRRSKINDRFQRLRELIPNIDQK** | **RDKASFLLEV** |
| 119. | CsbHLH119 | 295 | 7.80e-22 | **PGLGRDTPLN** | **HVEAERQRREKLNNRFYALRAVVPNVSRM** | **DKASLLSDAV** |
| 97. | CsbHLH97 | 44 | 1.00e-21 | **GKSREKNRSK** | **HSETEQRRRSKINERFQILRELIPQSDQK** | **RDKASFLLEL** |
| 96. | CsbHLH96 | 37 | 1.00e-21 | **GKSREKNRSK** | **HSETEQRRRSKINERFQILRELIPQSDQK** | **RDKASFLLEL** |
| 95. | CsbHLH95 | 44 | 1.00e-21 | **GKSREKNRSK** | **HSETEQRRRSKINERFQILRELIPQSDQK** | **RDKASFLLEV** |
| 66. | CsbHLH66 | 149 | 1.13e-21 | **NGRSPLHARD** | **HVIAERKRREKLNQKFIALSAVVPGLKKM** | **DKASVLGNAI** |
| 88. | CsbHLH88 | 329 | 1.28e-21 | **RATRGSATDP** | **QSLYARKRRERINERLRILQNIVPNGTKV** | **DISTMLEEAV** |
| 87. | CsbHLH87 | 247 | 1.28e-21 | **RARRGSATDP** | **QSLYARKRRERINERLRILQNIVPNGTKV** | **DISTMLEEAV** |
| 69. | CsbHLH69 | 177 | 1.28e-21 | **TSTKLSQTKD** | **HILAERKRREKLSQRFIALSAIVPGLKKM** | **DKASVLGDAI** |
| 67. | CsbHLH67 | 217 | 1.28e-21 | **ATRSPLHAQD** | **HVIAERKRREKLSQRFIALSAVVPGLKKM** | **DKASVLGDAI** |
| 42. | CsbHLH42 | 20 | 1.63e-21 | **SASKSETCKS** | **HKEAERRRRQRINAHLSTLRSLLPNITKT** | **DKASLLAEVV** |
| 54. | CsbHLH54 | 364 | 3.37e-21 | **KGKKKGLPAK** | **NLMAERRRRKKLNDRLYMLRSVVPKISKM** | **DRASILGDAI** |
| 84. | CsbHLH84 | 320 | 3.79e-21 | **RAKRGYATHP** | **RSIAERNRRTRISERIRRLQDLCPNMDKQ** | **TNTADMLELV** |
| 25. | CsbHLH25 | 57 | 3.79e-21 | **CGGVKLSTDP** | **QSVAARERRHRISERFKILQSLVPGGAKM** | **DTVSMLEEAI** |
| 89. | CsbHLH89 | 419 | 4.27e-21 | **RKNVRISTDP** | **QTVAARQRRERISERIRVLQKLVPGGSKM** | **DTASMLDEAA** |
| 46. | CsbHLH46 | 90 | 6.82e-21 | **EDKAAAASKS** | **HSQAEKRRRDRINAQLTTLRKLIPKSDKM** | **DKAALLGSVI** |
| 65. | CsbHLH65 | 159 | 9.66e-21 | **SKRPKVDRSR** | **TLISERRRRGRMKEKLYALRSLVPNITKM** | **DKASIVGDAV** |
| 70. | CsbHLH70 | 440 | 2.14e-20 | **CRPTTSTQLH** | **HMISERKRREKLNESFQSLRSLLPPGTKK** | **DKASVLNSTT** |
| 52. | CsbHLH52 | 187 | 2.68e-20 | **PKKLEGQPSK** | **NLMAERRRRKRLNDRLSMLRSIVPKISKM** | **DRTSILGDTI** |
| 111. | CsbHLH111 | 347 | 4.17e-20 | **RKSGKSDQCK** | **NLFAERRRRKKLNERLYTLRSLVPKISKL** | **DRASILGDAI** |
| 85. | CsbHLH85 | 283 | 4.17e-20 | **RAKRGCATHP** | **RSIAERERRTRISGKLKKLQDLVPNMDKQ** | **TSYADMLDLA** |
| 59. | CsbHLH59 | 228 | 5.19e-20 | **SEEVESQRMT** | **HIAVERNRRKQMNEHLRVLRSLMPGSYVQ** | **RGDQASIIGG** |
| 68. | CsbHLH68 | 201 | 5.78e-20 | **MIKTPSHAQD** | **HIMAERKRREKLTQRFIALSAIVPGLKKM** | **DKASVLGDAI** |
| 64. | CsbHLH64 | 193 | 8.00e-20 | **TNVKAGDKSK** | **TLISERRRRGRMKEKLYALRSLVPFITKM** | **DKASIVGDAV** |
| 48. | CsbHLH48 | 84 | 8.91e-20 | **HDRALVALKN** | **HKEAEKRRRERINSHLDKLRSLLPCNSKT** | **DKASLLAKVV** |
| 53. | CsbHLH53 | 56 | 9.92e-20 | **DGAASSAASK** | **NIVSERNRRKKLNDRLFALRAVVPKISKM** | **DKASIIKDAI** |
| 47. | CsbHLH47 | 90 | 1.23e-19 | **EDKAAAASKS** | **HSQAEKRRRDRINAQLTTLRKLIPKSDKA** | **ALLGSVIDHV** |
| 60. | CsbHLH60 | 3 | 1.52e-19 | **MS** | **HIAVERNRRKQMNEHLKVLRSLTPCFYIK** | **RGDQASIIGG** |
| 45. | CsbHLH45 | 65 | 1.87e-19 | **AERTIEALKN** | **HSEAEKRRRARINSHFDTLRSLTPGAKKM** | **DKASLLAEVI** |
| 113. | CsbHLH113 | 447 | 5.25e-19 | **RPEADEIGMN** | **HALAERKRRERLNEKFSILKGMVPSLSKE** | **DKVSILDDAI** |
| 112. | CsbHLH112 | 445 | 5.25e-19 | **RPEADEIGMN** | **HALAERKRRERLNEKFSILKGMVPSLSKE** | **DKVSILDDAI** |
| 55. | CsbHLH55 | 165 | 7.11e-19 | **KEELEIQRMT** | **HIAVERNRRKQMNEYLAVLRSLMPSSYVQ** | **RGDQASIIGG** |
| 58. | CsbHLH58 | 169 | 9.60e-19 | **KEEAETQRMT** | **HIAVERNRRKQMNEHLAVLRSLMPDSYVQ** | **RGDQASIVGG** |
| 62. | CsbHLH62 | 125 | 1.43e-18 | **TTEGQGNKMS** | **HIAIERNRRKQMNENLSVLRSLMPCFYVK** | **RGDQASIIGG** |
| 61. | CsbHLH61 | 125 | 1.43e-18 | **TTEGQGNKMS** | **HIAIERNRRKQMNENLSVLRSLMPCFYVK** | **RGDQASIIGG** |
| 79. | CsbHLH79 | 89 | 2.56e-18 | **SDPSMVKKLN** | **HNASERDRRKKINDLYSSLRALLPLSDQT** | **KKMSIPATVS** |
| 57. | CsbHLH57 | 162 | 2.82e-18 | **KEDIENQRMT** | **HIAVERNRRKQMNEYLSVLRSLMPDSYVQ** | **RGDQASIIGG** |
| 43. | CsbHLH43 | 24 | 5.00e-18 | **SYSKQIVAFK** | **HSEAERIRRMRINSQYATLRTILPNLIKM** | **DKASVLAETI** |
| 56. | CsbHLH56 | 180 | 1.16e-17 | **KEEVENQRMT** | **HIAVERNRRKQMNDYLAVLRSMMPSSYVQ** | **RGDQASIVGG** |
| 77. | CsbHLH77 | 80 | 3.14e-17 | **DDPTVIKKLC** | **HNASERDRRKKINSLYSSLRSLLPAADQM** | **KKLSNPSTIS** |
| 78. | CsbHLH78 | 101 | 4.11e-17 | **DLTMVEKKLN** | **HNASERDRRKKVNHMYSALRSLLPPSDHT** | **KKLSIPATVS** |
| 107. | CsbHLH107 | 418 | 1.39e-16 | **KRARNDQTQS** | **PLPAFKVRKEKMGDRITALQQLVSPFGKT** | **DTASVLSEAI** |
| 106. | CsbHLH106 | 419 | 1.39e-16 | **KRARNDQTQS** | **PLPAFKVRKEKMGDRITALQQLVSPFGKT** | **DTASVLSEAI** |
| 105. | CsbHLH105 | 419 | 1.39e-16 | **KRARNDQTQS** | **PLPAFKVRKEKMGDRITALQQLVSPFGKT** | **DTASVLSEAI** |
| 72. | CsbHLH72 | 74 | 1.39e-16 | **DSTSKKRART** | **KSIPERDRRMKMNESYDLLQSLVPGILPK** | **ASKDKVLHAA** |
| 51. | CsbHLH51 | 304 | 2.12e-16 | **DRGRDGRGTK** | **HFASERHRRVQMSDKYHVLKSLVPNPTKN** | **DRASVVGDAI** |
| 110. | CsbHLH110 | 400 | 7.95e-16 | **VKRPRIETPS** | **PLPTFKVRKEKLGDRITALQQLVSPFGKT** | **DTASVLHEAI** |
| 109. | CsbHLH109 | 400 | 7.95e-16 | **VKRPRIETPS** | **PLPTFKVRKEKLGDRITALQQLVSPFGKT** | **DTASVLHEAI** |
| 108. | CsbHLH108 | 399 | 7.95e-16 | **VKRPRIETPS** | **PLPTFKVRKEKLGDRITALQQLVSPFGKT** | **DTASVLHEAI** |
| 24. | CsbHLH24 | 210 | 9.34e-16 | **SSSLKKMIPP** | **SSLLARKRRQKISDKTRCLQKLLPWDKKM** | **DMATMLEETF** |
| 75. | CsbHLH75 | 140 | 1.77e-15 | **EKDTLIKKQE** | **HNAKERIRRMKISETYMALGSLLPSTRLS** | **KLKKRWTAPR** |
| 76. | CsbHLH76 | 148 | 1.87e-14 | **IEDLMVKKQD** | **HNAKEKIRRMKLSETYMALGSLLPTNSRP** | **SKKKWTAPII** |
| 98. | CsbHLH98 | 51 | 2.02e-14 | **KDGTVARKLQ** | **KADREKLRRDRLNEHFIELGSVLDPDRPK** | **NDKATILTDT** |
| 102. | CsbHLH102 | 74 | 2.17e-14 | **RPGSCSTTGS** | **KACREKMRRDKLNDRFLELGSILDPGRPP** | **KMDKGVILGE** |
| 103. | CsbHLH103 | 80 | 5.16e-14 | **RSESRNGSST** | **KACREKQRRDRLNDKFVELGSILEPGRPP** | **KTDKAAILID** |
| 101. | CsbHLH101 | 90 | 1.69e-13 | **SSGGASCSNY** | **KACREKLRRDRLNERFLELVAVMELGRLP** | **KTDKAAILSD** |
| 71. | CsbHLH71 | 94 | 1.94e-13 | **HEHEKERCYR** | **HMINERIRREKQKQSYFTLHSMLPVGTKN** | **DKNSIVQIAT** |
| 100. | CsbHLH100 | 81 | 2.23e-13 | **RTDSCRKQGT** | **KACREKMRRERLNDRFLDLSAVLEPDRPA** | **KTDKTAILDD** |
| 63. | CsbHLH63 | 140 | 2.23e-13 | **GGGGDSDHEI** | **HIWTERERRKKMRNMFANLHALLPQLPSK** | **ADKSTIVDEA** |
| 99. | CsbHLH99 | 42 | 7.42e-13 | **VDPVTARKVQ** | **KADREKLRRDKLNEYFLELGNTLDSDRPK** | **NDKGTILTDS** |
| 74. | CsbHLH74 | 77 | 9.63e-13 | **SDNKSKEMMK** | **HRDIERQRRQEMAALHASLRSLLPLELIK** | **GKRSISDHIN** |
| 73. | CsbHLH73 | 89 | 2.08e-12 | **TECKSNKMMK** | **HRDIERQRRQEMSTLHASLRSLLPLELIK** | **GKRSISDHIN** |
| 121. | CsbHLH121 | 538 | 2.22e-12 | **LKSTRGRSGN** | **IQKQRPRDRQLIQDRVKELRELVPNGAKC** | **SIDGLLDRTV** |
| 120. | CsbHLH120 | 472 | 2.22e-12 | **LKSTRGRSGN** | **IQKQRPRDRQLIQDRVKELRELVPNGAKC** | **SIDGLLDRTV** |

MEME MOTIF 4 E-value 1.1e-181 Sites 15 Width 30


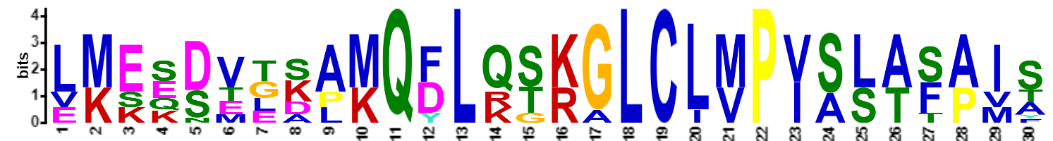


| 23. | CsbHLH23 | 407 | 6.52e-33 | **IVLFERELVK** | **LMESSVTKAMQFLQTKGLCLMPVALASAIS** | **TKKRNDQSPP** |
| --- | --- | --- | --- | --- | --- | --- |
| 22. | CsbHLH22 | 377 | 6.52e-33 | **IVLFERELVK** | **LMESSVTKAMQFLQTKGLCLMPVALASAIS** | **TKKRNDQSPP** |
| 21. | CsbHLH21 | 389 | 6.52e-33 | **IVLFERELVK** | **LMESSVTKAMQFLQTKGLCLMPVALASAIS** | **TKKRNDQSPP** |
| 20. | CsbHLH20 | 407 | 6.52e-33 | **IVLFERELVK** | **LMESSVTKAMQFLQTKGLCLMPVALASAIS** | **TKKRNDQSPP** |
| 19. | CsbHLH19 | 377 | 6.52e-33 | **IVLFERELVK** | **LMESSVTKAMQFLQTKGLCLMPVALASAIS** | **TKKRNDQSPP** |
| 16. | CsbHLH16 | 394 | 3.27e-31 | **MTVTEHQVAK** | **LMEEDMGSAMQYLQGKGLCLMPISLATAIS** | **TATCHTRNPM** |
| 15. | CsbHLH15 | 392 | 3.27e-31 | **MTVTEHQVAK** | **LMEEDMGSAMQYLQGKGLCLMPISLATAIS** | **TATCHTRNPM** |
| 18. | CsbHLH18 | 281 | 3.11e-29 | **TDGTEQQVAK** | **LMEEDVGAAMQFLQSKALCIMPISLASAIF** | **RTHQPDGPTM** |
| 17. | CsbHLH17 | 253 | 2.55e-28 | **SDGTERQVAK** | **LMEENVGAAMQFLQSKALCIMPISLASAIY** | **HSQPPDSSSV** |
| 110. | CsbHLH110 | 474 | 4.37e-26 | **QAHQQGSNKL** | **VKKQDELSPKQDLKSRGLCLVPISSTFPVA** | **NETTADFWTP** |
| 109. | CsbHLH109 | 472 | 4.37e-26 | **QAHQQGSNKL** | **VKKQDELSPKQDLKSRGLCLVPISSTFPVA** | **NETTADFWTP** |
| 108. | CsbHLH108 | 473 | 4.37e-26 | **QAHQQGSNKL** | **VKKQDELSPKQDLKSRGLCLVPISSTFPVA** | **NETTADFWTP** |
| 107. | CsbHLH107 | 488 | 4.37e-26 | **AAIQQQQNNS** | **EKSKDTEDLKQDLRSRGLCLVPVSSTFPMT** | **HETAVDFWTP** |
| 106. | CsbHLH106 | 489 | 4.37e-26 | **AAIQQQQNNS** | **EKSKDTEDLKQDLRSRGLCLVPVSSTFPMT** | **HETAVDFWTP** |
| 105. | CsbHLH105 | 487 | 4.37e-26 | **AAIQQQQNNS** | **EKSKDTEDLKQDLRSRGLCLVPVSSTFPMT** | **HETAVDFWTP** |

MEME MOTIF 5 E-value 4.5e-151 Sites 8 Width 41


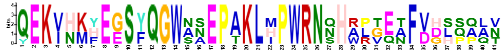


| 92. | CsbHLH92 | 201 | 1.94e-46 | **LEVIEYIQYL** | **QEKVHKHEGSFQGWANEPAKLMPWRNNHRLGEAFDHSQALN** | **GVSGPPLVFA** |
| --- | --- | --- | --- | --- | --- | --- |
| 91. | CsbHLH91 | 315 | 1.94e-46 | **LEVIEYIQYL** | **QEKVHKHEGSFQGWANEPAKLMPWRNNHRLGEAFDHSQALN** | **GVSGPPLVFA** |
| 90. | CsbHLH90 | 314 | 1.94e-46 | **LEVIEYIQYL** | **QEKVHKHEGSFQGWANEPAKLMPWRNNHRLGEAFDHSQALN** | **GVSGPPLVFA** |
| 97. | CsbHLH97 | 90 | 6.35e-46 | **LELIEYVQYL** | **QEKINMYEESYQGWNSEPAKLHPWRNQHAPTETFVDLSQAV** | **KNGSDLPNNV** |
| 96. | CsbHLH96 | 83 | 6.35e-46 | **LELIEYVQYL** | **QEKINMYEESYQGWNSEPAKLHPWRNQHAPTETFVDLSQAV** | **KNGSDLPNNV** |
| 95. | CsbHLH95 | 90 | 1.34e-44 | **LEVIVCMYKA** | **YEKINMYEESYQGWNSEPAKLHPWRNQHAPTETFVDLSQAV** | **KNGSDLPNNV** |
| 94. | CsbHLH94 | 96 | 8.18e-44 | **LEVIEYVQFL** | **QEKVHKFEGSYQGWSAEPTKLIPWRNSHWRVQNFVGHPPQT** | **IKNGPAAAFP** |
| 93. | CsbHLH93 | 96 | 8.18e-44 | **LEVIEYVQFL** | **QEKVHKFEGSYQGWSAEPTKLIPWRNSHWRVQNFVGHPPQT** | **IKNGPAAAFP** |

1. **Motif site distribution analysis of cannabis MYBs**

MEME Analysis I

Query: All CsMYBs amino acid sequences

Motif 1 (Logo sequence of **R3 repeat**) E-value: 2.1e-3487 Site Count: 104 Width: 50


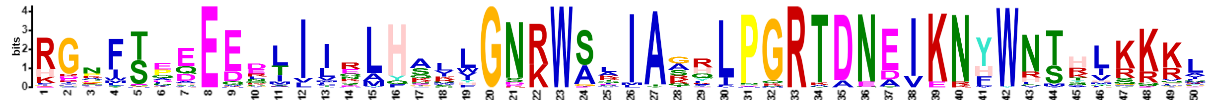


|  |  |  |  |  |  |  |
| --- | --- | --- | --- | --- | --- | --- |
| 38. | CsMYB48 | 67 | 8.42e-52 | **WINYLRPDLK** | **RGNFTEEEDELIIKLHSLLGNKWSLIAGRLPGRTDNEIKNYWNTHIRRKL** | **LNRGIDPATH** |
| 19. | CsMYB29 | 67 | 3.49e-51 | **WTNYLRPDIK** | **RGRFSFEEEETIIQLHSVLGNKWSAIAARLPGRTDNEIKNYWNTHIRKRL** | **LKMGIDPVTH** |
| 18. | CsMYB28 | 67 | 3.49e-51 | **WTNYLRPDIK** | **RGRFSFEEEETIIQLHSVLGNKWSAIAARLPGRTDNEIKNYWNTHIRKRL** | **LRMGIDPVTH** |
| 49. | CsMYB59 | 66 | 5.18e-51 | **WINYLRPDLK** | **RGNFAEDEEDLIIKLHALLGNRWSLIAGRLPGRTDNEIKNYWNSHLRRKL** | **INMGIDPNNH** |
| 12. | CsMYB22 | 67 | 5.18e-51 | **WINYLRPDLK** | **RGNFTEDEDELIINLHSLLGNKWSLIASRLPGRTDNEIKNYWNTHIKRKL** | **YSRGIDPQTH** |
| 11. | CsMYB21 | 67 | 5.18e-51 | **WINYLRPDLK** | **RGNFTEDEDELIINLHSLLGNKWSLIASRLPGRTDNEIKNYWNTHIKRKL** | **YSRGIDPQTH** |
| 20. | CsMYB30 | 67 | 6.30e-51 | **WTNYLRPDIK** | **RGRFSFEEEETIIQLHSILGNKWSAIAARLPGRTDNEIKNYWNTHIRKRL** | **LRMGIDPVTH** |
| 70. | CsMYB76 | 67 | 9.29e-51 | **WLNYLRPDIK** | **RGNISEEEEDLIIRLHKLLGNRWSLIAGRLPGRTDNEIKNYWNTTLAKKL** | **VQKQQQIEEN** |
| 47. | CsMYB57 | 66 | 1.65e-50 | **WINYLRPDIK** | **RGNFGEDEEDLIIKLHALLGNRWSLIAGRLPGRTDNEVKNYWNSHLRKKL** | **INLGIDPNNH** |
| 51. | CsMYB61 | 67 | 2.89e-50 | **WINYLRPDIK** | **RGNFTAEEEQTIINLHQMLGNRWSAIAARLPGRTDNEIKNVWHTHLKKRL** | **LNNNNINSNN** |
| 50. | CsMYB60 | 67 | 2.89e-50 | **WINYLRPDIK** | **RGNFTKEEEETIIELHQMLGNRWSAIAARLPGRTDNEIKNVWHTHLKKRL** | **IINNNDDVIV** |
| 5. | CsMYB15 | 67 | 5.03e-50 | **WTNYLRPDIK** | **RGKFSQEEEETILNLHSILGNKWSAIASHLPGRTDNEIKNFWNTHLKKKL** | **IQMGIDPMTH** |
| 54. | CsMYB62 | 67 | 7.24e-50 | **WINYLRPDLK** | **RGMFSQQEEDLIISLHQVLGNRWAQIAAQLPGRTDNEIKNFWNSCLKKKL** | **VKQGIDPTTH** |
| 88. | CsMYB93 | 79 | 8.68e-50 | **WINYLRPDLK** | **RGAFSPQEEELIIHLHSLLGNRWSQIAARLPGRTDNEIKNFWNSTVKKRL** | **KNLSSSSTTS** |
| 8. | CsMYB18 | 67 | 1.04e-49 | **WTNYLRPDIK** | **RGKFSEDEEKLIINLHAVLGNKWSTIAGHLPGRTDNEIKNLWNTHLKKKL** | **LQMGIDPVTH** |
| 6. | CsMYB16 | 60 | 1.04e-49 | **WTNYLRPDIK** | **RGKFTLQEERTIIQLHALLGNRWSAIASHLPNRTDNEIKNYWNTHLKKRL** | **TTMGIDPVTH** |
| 7. | CsMYB17 | 67 | 1.77e-49 | **WTNYLRPDIK** | **RGKFSLQEEQTIIQLHALLGNRWSAIATHLPKRTDNEIKNYWNTHLKKRL** | **TKMGIDPVTH** |
| 61. | CsMYB67 | 67 | 2.11e-49 | **WMNYLRPDIK** | **RGNITADEDDLIIRLHSLLGNRWSLIAGRLPGRTDNEIKNYWNSHLSKRL** | **NTNNKERTKK** |
| 17. | CsMYB27 | 68 | 5.96e-49 | **WTNYLRPDIK** | **RGPFTQDEEKLIIQLHGMLGNRWAAIASQLPGRTDNEIKNLWNTHLKKRL** | **LCMGLDPLTH** |
| 93. | CsMYB97 | 67 | 7.06e-49 | **WINYLRPDLK** | **RGPFSQQEENLIIELHAVLGNRWSQIAAQLPGRTDNEIKNLWNSCIKKKL** | **RQKGIDPNTH** |
| 92. | CsMYB96 | 67 | 7.06e-49 | **WINYLRPDLK** | **RGPFSQQEENLIIELHAVLGNRWSQIAAQLPGRTDNEIKNLWNSCIKKKL** | **RQKGIDPNTH** |
| 58. | CsMYB64 | 86 | 1.92e-48 | **WLNYLRPNIK** | **RGNITDQEEDLILRLHKLLGNRWSLIAGRLPGRTDNEIKNYWNSHLCKKM** | **SQKEKKKKKI** |
| 13. | CsMYB23 | 70 | 3.15e-48 | **WNNYLRPDIK** | **RGKFSEDEERIIINLHSALGNKWSRIASHLPGRTDNEIKNFWNTYLRKKL** | **LQMGIDPQTH** |
| 29. | CsMYB39 | 68 | 3.70e-48 | **WLNYLRPNIK** | **RGNISEAEEDLILRLHKLLGNRWALIAGRLPGRTDNEIKNYWNSHLSKKI** | **NQSQHQPSLS** |
| 14. | CsMYB24 | 67 | 5.11e-48 | **WTNYLRPGIK** | **RGPFSQEEESTIIQLHAMFGNRWAVIASQIPGRTDNEIKNYWNTHLKKRG** | **ICLAENLQHS** |
| 2. | CsMYB12 | 67 | 7.03e-48 | **WTNYLRPGIK** | **RGNFTDHEEKMIIHLQALLGNRWAAIASYLPQRTDNDIKNYWNTHLKKKL** | **RKLHTGHDGQ** |
| 9. | CsMYB19 | 67 | 6.12e-47 | **WTNYLRPDIK** | **RGKFSLQEEQTIIQLHALLGNRWSAIATHLAKRTDNEIKNYWNTHLKKRL** | **AKMGIDPITH** |
| 74. | CsMYB80 | 67 | 7.11e-47 | **WLNYLRPDIK** | **RGNISPDEEELIIRLHNLLGNRWSLIAGRLPGRTDNEIKNYWNTNIGKKV** | **QVAADHHNNF** |
| 66. | CsMYB72 | 87 | 1.73e-46 | **WANHLRPNLK** | **KGSFTPEEERLILELHAKYGNKWARMAAQLPGRTDNEIKNYWNTRVKRRK** | **RQGLPLYPHD** |
| 64. | CsMYB70 | 42 | 1.73e-46 | **WANHLRPNLK** | **KGSFTPEEERLILELHAKYGNKWARMAAQLPGRTDNEIKNYWNTRVKRRK** | **RQGLPLYPHD** |
| 62. | CsMYB68 | 87 | 1.73e-46 | **WANHLRPNLK** | **KGSFTPEEERLILELHAKYGNKWARMAAQLPGRTDNEIKNYWNTRVKRRK** | **RQGLPLYPHD** |
| 46. | CsMYB56 | 79 | 2.00e-46 | **WMNYLRPSVK** | **RGHIQPDEEDLILRLHRLLGNRWSLIAGRIPGRTDNEIKNYWNTHLSKKL** | **INQGIDPRTH** |
| 35. | CsMYB45 | 67 | 2.00e-46 | **WKNYLRPNIK** | **RGGMSQEEEDLIIRMHKLLGNRWSLIAGRLPGRTDNEVKNYWNTHLNKKT** | **SAVGKRKRVN** |
| 42. | CsMYB52 | 67 | 3.57e-46 | **WLNYLRPDIT** | **RGNISTDEDDLIVRLHRLLGNRWALIAGRLPGRTDNEIKNYWNTNLRKRV** | **NNINNININI** |
| 41. | CsMYB51 | 67 | 3.57e-46 | **WLNYLRPDIT** | **RGNISTDEDDLIVRLHRLLGNRWALIAGRLPGRTDNEIKNYWNTNLRKRV** | **NNINNININI** |
| 23. | CsMYB33 | 67 | 3.57e-46 | **WINYLRPDIR** | **RGRFTPEEEKLIISLHGVVGNRWAHIASHLPGRTDNEIKNYWNSWIKKKI** | **RKSSSTNSIN** |
| 22. | CsMYB32 | 67 | 3.57e-46 | **WINYLRPDIR** | **RGRFTPEEEKLIISLHGVVGNRWAHIASHLPGRTDNEIKNYWNSWIKKKI** | **RKSSSTNSIN** |
| 4. | CsMYB14 | 67 | 3.57e-46 | **WTNYLRPGIK** | **RGNFTDNEEKMIIHLQALLGNRWAAIASYLPQRTDNDIKNYWNTYLKKKL** | **SKIQNQSSSR** |
| 3. | CsMYB13 | 67 | 3.57e-46 | **WTNYLRPGIK** | **RGNFTDNEEKMIIHLQALLGNRWAAIASYLPQRTDNDIKNYWNTYLKKKL** | **SKIQNQSSSR** |
| 25. | CsMYB35 | 67 | 4.12e-46 | **WTNYLRPDLK** | **RGLLTEAEEQLVIDLHARLGNRWSKIASRLPGRTDNEIKNHWNTHIKKKL** | **LKMGIDPVTH** |
| 67. | CsMYB73 | 95 | 7.27e-46 | **WINYLRPDLK** | **RGAFSSHEQDLILHLHSILGNRWSQIAARLPGRTDNEIKNFWNSTLKKKL** | **NKLGVNNNNN** |
| 1. | CsMYB11 | 67 | 7.27e-46 | **WTNYLRPGIK** | **RGNFTPHEEGMIIHLQALLGNKWASIASYLPQRTDNDIKNYWNTHLKKKL** | **KKFHSALEPN** |
| 53. | CsMYB2 | 95 | 1.27e-45 | **WVNYLHPGLK** | **RGKMTPQEERLVLELHSKWGNRWSRIARKLPGRTDNEIKNYWRTHMRKKA** | **QEKKKSTTTT** |
| 52. | CsMYB1 | 62 | 1.27e-45 | **WVNYLHPGLK** | **RGKMTPQEERLVLELHSKWGNRWSRIARKLPGRTDNEIKNYWRTHMRKKA** | **QEKKKSTTTT** |
| 10. | CsMYB20 | 73 | 1.68e-45 | **WVNYLRPGLK** | **RGQITPQEEGIIIELHALWGNKWSTIARYLPGRTDNEIKNFWRTHFKKKD** | **KAKYSRKQQK** |
| 40. | CsMYB50 | 92 | 2.54e-45 | **WANHLRPNLK** | **KGSFSPDEERIIIELHAKIGNKWARMASQLPGRTDNEIKNYWNTRMKRRQ** | **RAGLPLYPHD** |
| 27. | CsMYB37 | 67 | 3.83e-45 | **WINYLRADLK** | **RGNISSEEEDIIINLHSTLGNRWSLIASHLPGRTDNEIKNYWNSHLSRKI** | **HTFRRCNTTH** |
| 15. | CsMYB25 | 68 | 3.83e-45 | **WINYLRPDIK** | **RGNFSKEEEDTIIKLHHNLGNRWSSIATQLPGRTDNEIKNFWHTHLKKKI** | **KKKLIMKQSS** |
| 39. | CsMYB49 | 67 | 4.38e-45 | **WANYLRPDIK** | **RGAFSPEEELTIVRLHSVLGNRWSAIAKNLPKRTDNEIKNHWNTRLKKCL** | **IESAAYHHQN** |
| 68. | CsMYB74 | 92 | 5.01e-45 | **WTNYLRPDLK** | **HGQFSDAEEQTIVKLHSIVGNRWSIIAAQLPGRTDNDVKNHWNTKLKKKL** | **SGMGIDPVTH** |
| 78. | CsMYB84 | 67 | 5.73e-45 | **WINYLRPDLK** | **RGGFTELEENQIIQLHSRLGNRWSKIAAHFPGRTDNEIKNHWNTRIKKRL** | **KVLGVDPITH** |
| 73. | CsMYB79 | 66 | 1.27e-44 | **WINYLRPDLR** | **KGSFTQEEEQIIIDVHRILGNRWAQIAKHLPGRTDNEVKNFWNSCIKKKL** | **MSQGLDPQTH** |
| 24. | CsMYB34 | 69 | 1.27e-44 | **WINYLRPDVK** | **RGNFSNEEEEAIIKLHETLGNKWSKIASHFPGRTDNEIKNVWNTHLKKRL** | **SSSSKDGDDQ** |
| 60. | CsMYB66 | 67 | 1.89e-44 | **WTNYLRPGIK** | **RGEFTNEEEEIIMRLHAVLGNKWSAIAKQLPMRTDNEIKNHWNTRLKRIV** | **AEKGKDNLIT** |
| 34. | CsMYB44 | 68 | 1.89e-44 | **WINYLRPGLK** | **RGTFSQHEEETILTLHHMLGNKWSQIAQHLPGRTDNEIKNYWHSYLKKKV** | **AKAEEELLTE** |
| 76. | CsMYB82 | 82 | 3.17e-44 | **WLNYLKPDIK** | **RGEFTADEVDLILRLHKLLGNRWSLIAGRIPGRTANDVKNYWNTHLGKKV** | **MMSYDKLNQK** |
| 26. | CsMYB36 | 68 | 4.11e-44 | **WVNYLRPDIK** | **HGNFSQEEKEIIVTLHETLGNRWSAIAAKLPGRTDNEVKNYWHTHLKKRF** | **QKQLPLHSSS** |
| 77. | CsMYB83 | 67 | 4.67e-44 | **WTNYLRPDLK** | **HDTFTPQEEDLIINLHQAIGSRWSLIAKQLPGRTDNDVKNYWNTKLRKKL** | **YTMGIDPVTH** |
| 16. | CsMYB26 | 67 | 5.30e-44 | **WTNYLRPDLK** | **RGLLSDYEEQMVIDLHAQLGNRWSKIASHLPGRTDNEIKNHWNTHIKKKL** | **KKMGIDPLTH** |
| 89. | CsMYB94 | 108 | 6.02e-44 | **WLNYLRPDVR** | **RGNITLEEQLLILELHSRWGNRWSKIAQHLPGRTDNEIKNYWRTRVQKHA** | **KQLKCDVNSK** |
| 87. | CsMYB92 | 71 | 7.76e-44 | **WLNYLKPDIK** | **RGNLTPQEQLLILELHSKWGNRWSRIAQHLPGRTDNEIKNYWRTRVQKQA** | **RQLNIESNSK** |
| 86. | CsMYB91 | 71 | 7.76e-44 | **WLNYLKPDIK** | **RGNLTPQEQLLILELHSKWGNRWSRIAQHLPGRTDNEIKNYWRTRVQKQA** | **RQLNIESNSS** |
| 80. | CsMYB86 | 73 | 7.76e-44 | **WLNYLRPDVR** | **RGNITNEEQLIIMELHAKWGNRWSKIAKHLPGRTDNEIKNYWRTRIQKHM** | **KQADHHPNQN** |
| 36. | CsMYB46 | 75 | 7.76e-44 | **WVNYLRPDLK** | **RGQITPHEESIILELHARWGNRWSTIARSLPGRTDNEIKNYWRTHFKKKQ** | **AKVTSDASEK** |
| 72. | CsMYB78 | 67 | 1.28e-43 | **WINYLRPDLK** | **RGSFSPQEAALIIELHTILGNRWAQIAKHLPGRTDNEVKNFWNSSIKKKL** | **LSGHDNHLHH** |
| 71. | CsMYB77 | 92 | 1.45e-43 | **WLNYLRPSVR** | **RGNITLQEQLLILELHSRWGNRWSKIAQYLPGRTDNEIKNYWRTRVQKQA** | **KQLKCDVNSK** |
| 45. | CsMYB55 | 75 | 1.45e-43 | **WLNYLRPNLK** | **HDQISAEEENIIIQLHERWGNKWSKIARILPGRTDNEIKNYWRTYLRKKL** | **AQNQEEKGKE** |
| 44. | CsMYB54 | 63 | 1.45e-43 | **WLNYLRPNLK** | **HDQISAEEENIIIQLHERWGNKWSKIARILPGRTDNEIKNYWRTYLRKKL** | **AQNQEEKGKE** |
| 59. | CsMYB65 | 67 | 3.06e-43 | **WMNYLRPDVR** | **RGNYTAEEENTIIKLHQQHGKKWSMIAAKLPGRTDNEIKNHWHTHLKKRA** | **ITNNYNNNNN** |
| 21. | CsMYB31 | 68 | 4.41e-43 | **WLNYLRPNIK** | **HGEFSDEEDRIICNLFANIGSRWSIIAAHLPGRTDNDIKNYWNTKLKKKL** | **MGLAPQISQR** |
| 43. | CsMYB53 | 67 | 5.62e-43 | **WINYLRADLK** | **RGNITSEEEETIVNLHNALGNRWSVIADHLPGRTDNEIKNYWNSHLSRKI** | **YSFTKRLPNE** |
| 65. | CsMYB71 | 113 | 7.16e-43 | **WCNQLDPCVK** | **RKPFTEEEDRLIVTAHAIHGNKWAAIARLLPGRTDNAIKNHWNSTLKRKY** | **PECGRFNTGP** |
| 63. | CsMYB69 | 109 | 7.16e-43 | **WCNQLDPCVK** | **RKPFTEEEDRLIVTAHAIHGNKWAAIARLLPGRTDNAIKNHWNSTLKRKY** | **PECGRFNTGP** |
| 57. | CsMYB63 | 78 | 1.47e-42 | **WLNYLKPDVK** | **RGNLTPEEQILILDLHSKWGNRWSKIAQYLPGRTDNEIKNYWRTRVQKQA** | **KHLKIDAEST** |
| 28. | CsMYB38 | 67 | 1.65e-42 | **WMNYLRPNVK** | **RGNYTHEEEETITRLHASLGNKWSAIAAELPGRTDNEVKNYWHTNLKKRQ** | **KQNSSSVEIS** |
| 69. | CsMYB75 | 67 | 2.09e-42 | **WANYLRPGIK** | **RGEFSPEEEQTIIRLHAILGNKWSIISRHLYRRTDNEVKNYWNTRLKKRG** | **TTEINSKDSP** |
| 81. | CsMYB87 | 68 | 2.97e-42 | **WLNYLKPDIK** | **RGEFEEDEVDLVLRLHKLLGNRWSLIAGRIPGRTANDVKNYWNTHQRKKM** | **VIINIDQSNK** |
| 75. | CsMYB81 | 67 | 3.34e-42 | **WNNHLRPDLN** | **HESFTPKEEELIVKLHAAIGSRWSMIAQQLPGRTDNDVKNYWNTKLRKKL** | **SEMGIDPVTH** |
| 32. | CsMYB42 | 68 | 4.73e-42 | **WLNYLRPNIK** | **HGGFSEEEDNIICSLYISIGSRWSIIAAQLPGRTDNDIKNYWNTRLKKKL** | **LGNKQRKDHP** |
| 31. | CsMYB41 | 68 | 4.73e-42 | **WLNYLRPNIK** | **HGGFSEEEDNIICSLYISIGSRWSIIAAQLPGRTDNDIKNYWNTRLKKKL** | **LGRRKQSNGS** |
| 33. | CsMYB43 | 68 | 1.18e-41 | **WLNYLRPHIK** | **HGGFSEEEDNIICSLYLSIGSRWSIIAAQLPGRTDNDIKNYWNTRLKKKL** | **LGKQRKEHAA** |
| 84. | CsMYB90 | 65 | 2.91e-41 | **WCNQLSPQVE** | **HRAFTAEEDDTIIRAHARFGNKWATIARLLNGRTDNAIKNHWNSTLKRKC** | **STMLEETDNG** |
| 83. | CsMYB89 | 65 | 2.91e-41 | **WCNQLSPQVE** | **HRAFTAEEDDTIIRAHARFGNKWATIARLLNGRTDNAIKNHWNSTLKRKC** | **STMLEETDNG** |
| 37. | CsMYB47 | 68 | 1.09e-40 | **WLNYLRPDIK** | **HGGFTEEEDNVIWTLYSNIGSRWSVIASQLPGRTDNDVKNYWNTKLKKKL** | **LARSTNSNET** |
| 91. | CsMYB95 | 93 | 2.09e-40 | **WCNQLSPSVE** | **HRPFSPAEDETILAAHAQYGNRWATIARLLPGRTDNAVKNHWNSTLKRRA** | **REAHQMEDEG** |
| 82. | CsMYB88 | 60 | 2.10e-39 | **WCNQLCPSVQ** | **HRPFTPEEDSMIIQAHAAHGNKWATIARLLPGRTDNAIKNHWNSTLRRGR** | **RAGDKFSSSS** |
| 79. | CsMYB85 | 62 | 1.74e-38 | **WCNQLSPEVK** | **HRPFTDMEDQIIIDAHSKHGNKWATIARLLEGRTDNAIKNHWNSTLKRKC** | **SSSENDDVDD** |
| 56. | CsMYB100 | 179 | 3.44e-37 | **WHNHLNPEIR** | **KDAWTIAEELSLMHAHQIHGNKWAEIAKVLPGRTDNAIKNHWNSSLKKKL** | **DFFINTGSLP** |
| 55. | CsMYB99 | 163 | 3.44e-37 | **WHNHLNPEIR** | **KDAWTIAEELSLMHAHQIHGNKWAEIAKVLPGRTDNAIKNHWNSSLKKKL** | **DFFINTGSLP** |
| 95. | CsMYB104 | 146 | 4.55e-37 | **WHNHLNPAIN** | **KEPWTQEEELTLIRAHQVFGNKWAELTKFLPGRADNAIKNHWNSSVKKKL** | **ETYLASGLLD** |
| 94. | CsMYB103 | 146 | 4.55e-37 | **WHNHLNPAIN** | **KEPWTQEEELTLIRAHQVFGNKWAELTKFLPGRADNAIKNHWNSSVKKKL** | **ETYLASGLLD** |
| 30. | CsMYB40 | 66 | 4.55e-37 | **VNKLRPDLKT** | **GCKFSAEEERLVIELQAQVGNKWAKIATYLPGRTDNDVKNFWSSRKKKLQ** | **RLNHHRRPSS** |
| 85. | CsMYB101 | 137 | 1.99e-36 | **WHNHLNPAIN** | **KEAWTQEEELALIRAHQVYGNKWAELTKFLPGRTDNSIKNHWNSSVKKKL** | **DSYLKSGLLT** |
| 48. | CsMYB58 | 65 | 3.43e-36 | **VNKLRPNLKN** | **GCKFSLEEERVVIELQAQFGNKWARIATYLPGRTDNDVKNFWSSRQKRLA** | **RILQTPPTSS** |
| 90. | CsMYB102 | 131 | 4.49e-36 | **WHNHLDPSLN** | **KNPWTEEEESTLIKYHQIYGNKWADIAKFLPGRSDNAIKNHWHCKVKKKM** | **QVVSSQLYSP** |
| 99. | CsMYB6 | 24 | 2.01e-32 | **QVEVSSIEWE** | **FIHMSEQEEDLIFRMYKLVGDRWGLIAGRIPGRKAEEIERFWLMKHRQVF** | **DK** |
| 96. | CsMYB3 | 23 | 2.01e-32 | **SQEVSSIEWE** | **FIHMSEQEEDLIFRMYKLVGDRWGLIAGRIPGRKAEEIERFWLMKHRQVF** | **DK** |
| 102. | CsMYB9 | 35 | 2.35e-32 | **SEESIRREVV** | **EVEFSEDEESLIIRMFNLIGERWSLIAGRIPGRTAEEIEKYWVSKYSNHS** | **TKTNNNNQQL** |
| 101. | CsMYB8 | 34 | 2.35e-32 | **SEESIRREVV** | **EVEFSEDEESLIIRMFNLIGERWSLIAGRIPGRTAEEIEKYWVSKYSNHS** | **TKTNNNNQQL** |
| 104. | CsMYB98 | 75 | 1.63e-31 | **WYNQLDPNIN** | **KKPFTEEEEERLLAAHRIYGNKWACIAKYFHGRTDNAVKNHYHVVMARRK** | **RERFSSSSSS** |
| 98. | CsMYB5 | 33 | 2.58e-31 | **DQEVCSLEWE** | **FINMTEQEEDLIYRMFSLVGNRWDLIAGRVPGRTAQEIERFWIMRHHDMF** | **AERRKNQTK** |
| 100. | CsMYB7 | 45 | 6.88e-31 | **VEVSSIEWEF** | **IHMSEEQEEDLIFRMYKLVGDRWGLIAGRIPGRKAEEIERFWLMKHRQVF** | **DK** |
| 97. | CsMYB4 | 44 | 6.88e-31 | **QEVSSIEWEF** | **IHMSEEQEEDLIFRMYKLVGDRWGLIAGRIPGRKAEEIERFWLMKHRQVF** | **DK** |
| 103. | CsMYB10 | 29 | 1.12e-29 | **STEESSKLEF** | **KLDFSEDEEALITRMFNLVGERWTLIAGRIPGRTAEEIEKYWSTRYSTSE** |  |
|  |  |  |  |  |  |  |

MEME Motif 3 (Logo sequence of **R2 repeat**) E-value: 2.9e-1108 Site Count: 93 Width: 21


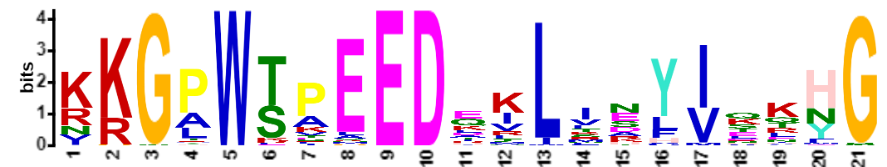


| 20. | CsMYB30 | 13 | 2.04e-26 | **RAPCCDKNGL** | **KKGPWTPEEDQKLVDYIQKHG** | **YGNWRTLPKN** |
| --- | --- | --- | --- | --- | --- | --- |
| 51. | CsMYB61 | 13 | 1.21e-23 | **RAPCCEKMGL** | **KKGPWTPEEDQILINYVQKFG** | **HANWRALPKQ** |
| 9. | CsMYB19 | 13 | 1.21e-23 | **RSPCCDKVGL** | **KKGPWTPEEDQKLLAYIEQHG** | **HGSWRALPAK** |
| 50. | CsMYB60 | 13 | 1.54e-23 | **RAPCCEKMGL** | **KKGPWTPEEDQILISYIHKYG** | **HGNWRALPKL** |
| 14. | CsMYB24 | 13 | 3.96e-23 | **KIACCDKNGV** | **KKGAWTPEEDQILVNYIKRHG** | **HGTWRSLPKH** |
| 39. | CsMYB49 | 13 | 7.71e-23 | **RSPCCNVQGL** | **KKGAWTPEEDQKLLSYIQQHG** | **EGGWRSLPQK** |
| 7. | CsMYB17 | 13 | 9.56e-23 | **RSPCCEKVGL** | **KKGPWTPEEDQKLLAYIEEHG** | **HGSWRALPAK** |
| 8. | CsMYB18 | 13 | 3.26e-22 | **RSPSCDENGL** | **KKGPWTPEEDEKLVDYITRNG** | **HGSWRALPKL** |
| 4. | CsMYB14 | 13 | 3.26e-22 | **RPPCCDKEGV** | **KKGPWTPEEDIILVSYIQEHG** | **PGNWRAVPTN** |
| 3. | CsMYB13 | 13 | 3.26e-22 | **RPPCCDKEGV** | **KKGPWTPEEDIILVSYIQEHG** | **PGNWRAVPTN** |
| 2. | CsMYB12 | 13 | 3.26e-22 | **RPPCCDKVGV** | **KKGPWTPEEDIILVSYIQEHG** | **PGNWRSVPTH** |
| 1. | CsMYB11 | 13 | 3.26e-22 | **RPPCCDKVGI** | **KKGPWTPEEDIILVSYIQEHG** | **PGNWRSVPTN** |
| 13. | CsMYB23 | 15 | 3.95e-22 | **PCCDGINIGL** | **KKGPWTPEEDKKLIDYIERNG** | **HHGSWKSLPK** |
| 5. | CsMYB15 | 13 | 5.79e-22 | **RSPCCDESGL** | **KKGPWTPEEDQKLVKFIQKNG** | **HGSWRALPRL** |
| 21. | CsMYB31 | 13 | 1.01e-21 | **RSPCCDKANV** | **KKGPWSPEEDLKLKEYIEKYG** | **TGGNWIALPQ** |
| 15. | CsMYB25 | 14 | 2.04e-21 | **RRACCENMGL** | **RKGPWTPEEDHILISYIQQFG** | **HANWRALPKQ** |
| 93. | CsMYB97 | 13 | 6.54e-21 | **RHSCCYKQKL** | **RKGLWSPEEDEKLLNYITKHG** | **HGCWSSVPKL** |
| 92. | CsMYB96 | 13 | 6.54e-21 | **RHSCCYKQKL** | **RKGLWSPEEDEKLLNYITKHG** | **HGCWSSVPKL** |
| 69. | CsMYB75 | 13 | 6.54e-21 | **RTPCCDQKGL** | **RKGAWTAEEDQILFSYIKQHG** | **EGGWRHLPQK** |
| 6. | CsMYB16 | 6 | 6.54e-21 | **MVDRL** | **KRGPWKPEEDKKLLAYIQQHG** | **HGSWRLVPKK** |
| 23. | CsMYB33 | 13 | 1.23e-20 | **HHSCCNQQKV** | **KRGLWSPEEDEKLIRYITTHG** | **YGCWSEVPEK** |
| 22. | CsMYB32 | 13 | 1.23e-20 | **HHSCCNQQKV** | **KRGLWSPEEDEKLIRYITTHG** | **YGCWSEVPEK** |
| 31. | CsMYB41 | 13 | 1.93e-20 | **RAPCCDKANV** | **KKGPWSPEEDTKLKAYIDTYG** | **TGGNWIALPQ** |
| 72. | CsMYB78 | 13 | 2.24e-20 | **HHSCCNKQKV** | **KRGLWSPEEDEKLINYISTYG** | **HGCWSSVPKL** |
| 28. | CsMYB38 | 13 | 2.60e-20 | **RTPCCDQNGM** | **KKGTWTPEEDRKLVAYVTRYG** | **CWNWRQLPKF** |
| 26. | CsMYB36 | 14 | 3.01e-20 | **KSRLCEKTHL** | **KKGVWSPEEDQKLRSYIKKYG** | **IWNWTEMSKA** |
| 18. | CsMYB28 | 13 | 3.01e-20 | **RAPCCDKNGL** | **KKGPWTPEEDLNLINYIQKNG** | **PGNWRNLPKN** |
| 60. | CsMYB66 | 13 | 4.02e-20 | **RTPCCNVQGL** | **KKGAWTTEEDQKLSAYITQHG** | **EGGWRSLPEK** |
| 43. | CsMYB53 | 13 | 4.63e-20 | **RAPCCEKLGL** | **KKGRWTAEEDQVLTNYIQTHG** | **EGSWRSLPKN** |
| 66. | CsMYB72 | 33 | 7.05e-20 | **CVSGPDGAIL** | **KKGPWTAAEDHVLMEYVKKHG** | **EGNWNSVQKY** |
| 62. | CsMYB68 | 33 | 7.05e-20 | **CVSGPDGAIL** | **KKGPWTAAEDHVLMEYVKKHG** | **EGNWNSVQKY** |
| 59. | CsMYB65 | 13 | 7.05e-20 | **RAPFIDENGV** | **KRGAWSPEEDDKLRAYVERYG** | **HWNWRELPKF** |
| 25. | CsMYB35 | 13 | 1.39e-19 | **RQPCCDKLGV** | **KKGPWTAEEDKKLINFILTNG** | **HCCWRAVPKL** |
| 16. | CsMYB26 | 13 | 1.39e-19 | **RQPCCDKVGL** | **KKGPWTAEEDKKLINFILTNG** | **QCCWRAVPKL** |
| 12. | CsMYB22 | 13 | 1.39e-19 | **RSPCCEKEHT** | **NKGAWTKEEDQRLIHYIKLHG** | **EGCWRSLPKA** |
| 11. | CsMYB21 | 13 | 1.39e-19 | **RSPCCEKEHT** | **NKGAWTKEEDQRLIHYIKLHG** | **EGCWRSLPKA** |
| 33. | CsMYB43 | 13 | 1.81e-19 | **RAPCCDKANV** | **KKGPWSPEEDATLKAYIEQNG** | **TGGNWIALPQ** |
| 19. | CsMYB29 | 13 | 1.81e-19 | **KITCCEKNGL** | **KKGPWTPEEDQALIDHIKRNG** | **HGRWRTLPKN** |
| 32. | CsMYB42 | 13 | 2.06e-19 | **RAPCCDKANV** | **KKGPWSVEEDAKLKSYIENHG** | **TGGNWIALPQ** |
| 57. | CsMYB63 | 24 | 2.66e-19 | **SSSEDDGNEL** | **RRGPWTVEEDTLLIHYITRHG** | **EGRWNLLAKR** |
| 80. | CsMYB86 | 19 | 3.90e-19 | **SGNSSEEVEV** | **RKGPWTMEEDLILINYIANHG** | **EGVWNSLAKA** |
| 49. | CsMYB59 | 12 | 8.13e-19 | **RKPCCEKQDK** | **NKGAWSIEEDQKLIDYINQHG** | **EGCWRTLPKA** |
| 54. | CsMYB62 | 13 | 9.17e-19 | **RHSCCLKQKL** | **RKGLWSPEEDEKLFNYITHFG** | **VGCWSSVPKL** |
| 10. | CsMYB20 | 19 | 9.17e-19 | **MTNQDQQQGW** | **RKGPWTPEEDKMLTEYVSFNG** | **EGRWSSVARS** |
| 56. | CsMYB100 | 126 | 1.16e-18 | **RWQKVLNPDL** | **VKGPWTPEEDKKITELVSKYG** | **ATKWSLIAKS** |
| 55. | CsMYB99 | 110 | 1.16e-18 | **RWQKVLNPDL** | **VKGPWTPEEDKKITELVSKYG** | **ATKWSLIAKS** |
| 17. | CsMYB27 | 13 | 1.16e-18 | **RTPCCDKKGL** | **KKGPWTAEEDETLVEYIKKNN** | **GHGSWRSLPK** |
| 36. | CsMYB46 | 21 | 1.65e-18 | **GWGIIEEEGW** | **RKGPWTSEEDRLLIEYVRLHG** | **EGRWNSVARL** |
| 30. | CsMYB40 | 11 | 2.08e-18 | **MKRGSDGEYI** | **RKGPWRAEEDEVLINHVKKNG** | **PRDWSSIRSK** |
| 48. | CsMYB58 | 10 | 2.33e-18 | **MVGNREEGI** | **RKGPWKAEEDEVLINHVNKYG** | **PRDWSSIRSK** |
| 89. | CsMYB94 | 54 | 2.92e-18 | **HDQEMNNSDL** | **RRGPWTVEEDLTLINYIAIHG** | **EGRWNSLARC** |
| 67. | CsMYB73 | 41 | 3.27e-18 | **NNKMIKNVKL** | **RKGLWSPEEDEKLMRYMLRNG** | **QGCWSDIARN** |
| 38. | CsMYB48 | 13 | 3.65e-18 | **RSPCCEKAHT** | **NKGAWTKEEDDRLIAYIRAHG** | **EGCWRSLPKA** |
| 61. | CsMYB67 | 13 | 4.08e-18 | **RTACCSRFGL** | **RRGPWTPREDTLLVNYIQLHG** | **EGHWRIVPKK** |
| 40. | CsMYB50 | 38 | 4.08e-18 | **PGGSRGGAGL** | **KKGPWTTSEDAILVEYVRKHG** | **EGNWNAVQKN** |
| 87. | CsMYB92 | 17 | 5.08e-18 | **KFNNEEEIEL** | **RRGPWTLEEDTLLIHYISLHG** | **EGHWNLLAKR** |
| 86. | CsMYB91 | 17 | 5.08e-18 | **KFNNEEEIEL** | **RRGPWTLEEDTLLIHYISLHG** | **EGHWNLLAKR** |
| 68. | CsMYB74 | 38 | 5.66e-18 | **RIPCCEKENV** | **KRGQWTPEEDNKLSSYIAQHG** | **TRNWRLIPKN** |
| 34. | CsMYB44 | 14 | 5.66e-18 | **KSQDKPKPKH** | **RKGLWSPEEDLRLRNFVLKHG** | **HGCWSSVPIK** |
| 29. | CsMYB39 | 14 | 6.31e-18 | **KKEGSSKKVM** | **NRGAWTAEEDKILSQYIKLHG** | **PRRWKTLSIQ** |
| 73. | CsMYB79 | 12 | 7.83e-18 | **GHRCCNKLKV** | **KRGLWSPEEDEKLLTHITTHG** | **HGNWSSVPKL** |
| 71. | CsMYB77 | 38 | 9.68e-18 | **EEDHHRQLDL** | **RKGPWTVEEDNMLFNYVSIHG** | **EGRWNSLARH** |
| 46. | CsMYB56 | 25 | 1.08e-17 | **TTPCCSKVGI** | **KRGPWTAEEDEVLCEFIRKEG** | **EGRWRTLPKR** |
| 47. | CsMYB57 | 12 | 1.20e-17 | **RKPCCDKDGT** | **NKGAWSKHEDQKLIDYITTHG** | **EGCWRSLPKA** |
| 85. | CsMYB101 | 84 | 1.47e-17 | **RWQKVLNPEL** | **VKGPWSKEEDDVIVELVKQYG** | **PKKWSTIANH** |
| 88. | CsMYB93 | 25 | 1.81e-17 | **NNNTNNNNKL** | **RKGLWSPEEDDKLMNYMLNNG** | **QGCWSDVARN** |
| 70. | CsMYB76 | 13 | 1.81e-17 | **RSPCCSKKGL** | **NKGAWTVLEDQILTDYIKTHG** | **EGKWRNLPKQ** |
| 95. | CsMYB104 | 93 | 2.46e-17 | **RWQKVLNPEL** | **IKGPWSKEEDEVIVELVNKYG** | **AKKWSMIAEA** |
| 94. | CsMYB103 | 93 | 2.46e-17 | **RWQKVLNPEL** | **IKGPWSKEEDEVIVELVNKYG** | **AKKWSMIAEA** |
| 81. | CsMYB87 | 14 | 2.72e-17 | **VLVKKSSLNV** | **KKGAWSREEDIVLRECIDKYG** | **EGKWHLVPLR** |
| 84. | CsMYB90 | 12 | 3.01e-17 | **ATITRKEMDR** | **IKGPWSPEEDDSLQKLVEKHG** | **PRNWSLISKS** |
| 83. | CsMYB89 | 12 | 3.01e-17 | **ATITRKEMDR** | **IKGPWSPEEDDSLQKLVEKHG** | **PRNWSLISKS** |
| 79. | CsMYB85 | 9 | 3.33e-17 | **MESLLSRK** | **VKGPWSPEEDEKLQSLVKQHS** | **ARNWSVISKS** |
| 65. | CsMYB71 | 60 | 3.33e-17 | **GDGDGGGDGR** | **VKGPWSPEEDVVLSRLVSKFG** | **ARNWSLIARG** |
| 63. | CsMYB69 | 56 | 3.33e-17 | **GDGDGGGDGR** | **VKGPWSPEEDVVLSRLVSKFG** | **ARNWSLIARG** |
| 78. | CsMYB84 | 13 | 3.67e-17 | **RQPCCDKIGL** | **KRGPWTIEEDHKLMNFILNNG** | **IHCWRMVPKL** |
| 24. | CsMYB34 | 15 | 4.06e-17 | **RAPCCDKSQV** | **KRGPWSPAEDLRLITFIQKNG** | **HENWRALPKQ** |
| 37. | CsMYB47 | 13 | 4.94e-17 | **RAPCCDKTKV** | **KRGPWSPEEDAALKHYMHNNG** | **TGGNWIALPH** |
| 58. | CsMYB64 | 32 | 5.45e-17 | **EEENNFNFKF** | **NRGAWTAEEDQKLAEVISHHG** | **AQKWKTVAAI** |
| 76. | CsMYB82 | 28 | 9.74e-17 | **EGGSSGGRAV** | **RKGAWTREEDDLLRDCVDKYG** | **EGKWHLVPLR** |
| 35. | CsMYB45 | 13 | 9.74e-17 | **ENKRVKKQLP** | **KKNLWKPEEDLILKNYVETHG** | **EGNWQTVSKL** |
| 27. | CsMYB37 | 13 | 1.07e-16 | **RAPCCEKIGL** | **KKGRWTSEEDEILTKYIQSNG** | **EGSWRSLPKN** |
| 77. | CsMYB83 | 13 | 1.29e-16 | **RPPCCDKSNV** | **KRGLWTAEEDAKLLAHVSKLG** | **IGNWTLVPKK** |
| 91. | CsMYB95 | 40 | 1.72e-16 | **VPRNGNKPER** | **IKGPWSTEEDRILTRLVDRYG** | **ARNWSLISRY** |
| 74. | CsMYB80 | 13 | 2.27e-16 | **RSPCCSKEGL** | **NRGAWTAMEDRILSEYIKVHG** | **EGKWRSLPKR** |
| 42. | CsMYB52 | 13 | 4.29e-16 | **RSPCCEKDGL** | **NRGAWSALEDKILADYIHKNG** | **PGKWRDLPKR** |
| 41. | CsMYB51 | 13 | 4.29e-16 | **RSPCCEKDGL** | **NRGAWSALEDKILADYIHKNG** | **PGKWRDLPKR** |
| 75. | CsMYB81 | 13 | 9.51e-16 | **RPSSCEKANL** | **KGGLWSEEEDARMLEYVSKHG** | **KAKWTSVPKG** |
| 45. | CsMYB55 | 21 | 1.74e-15 | **RAMQGEHDQL** | **RKGTWLEEEDERLISFVQLMG** | **EKRWDALAKA** |
| 44. | CsMYB54 | 9 | 1.74e-15 | **MQGEHDQL** | **RKGTWLEEEDERLISFVQLMG** | **EKRWDALAKA** |
| 90. | CsMYB102 | 78 | 3.41e-15 | **RWQKVLNPAL** | **VKGPWEKEEDKQLIKLVGEYG** | **VKRWSTIAKF** |
| 82. | CsMYB88 | 7 | 7.15e-15 | **MKGGDR** | **IKGSWSPQEDETLKKLVEQHG** | **PRNWSVISTG** |
| 104. | CsMYB98 | 22 | 2.54e-14 | **DHGGGVGKNC** | **YRGHWRPGEDEKLRELVDRYG** | **PQNWNFIAEH** |

MEME Motif 2 (Logo sequence of **SANT domain**) E-value: 1.7e-1662 Site Count: 96 Width: 21


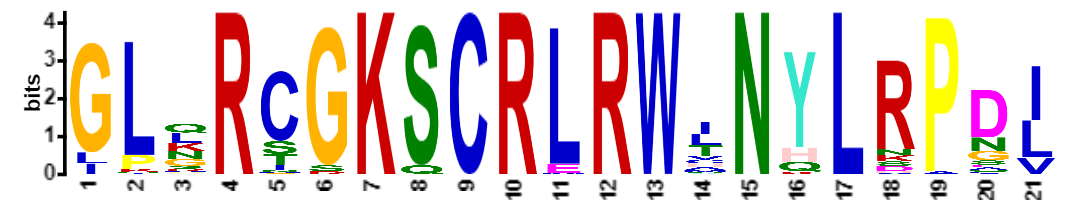


| 23. | CsMYB33 | 45 | 3.50e-28 | **GCWSEVPEKA** | **GLQRCGKSCRLRWINYLRPDI** | **RRGRFTPEEE** |
| --- | --- | --- | --- | --- | --- | --- |
| 22. | CsMYB32 | 45 | 3.50e-28 | **GCWSEVPEKA** | **GLQRCGKSCRLRWINYLRPDI** | **RRGRFTPEEE** |
| 20. | CsMYB30 | 45 | 9.40e-28 | **GNWRTLPKNA** | **GLQRCGKSCRLRWTNYLRPDI** | **KRGRFSFEEE** |
| 18. | CsMYB28 | 45 | 9.40e-28 | **GNWRNLPKNA** | **GLQRCGKSCRLRWTNYLRPDI** | **KRGRFSFEEE** |
| 9. | CsMYB19 | 45 | 9.40e-28 | **GSWRALPAKA** | **GLQRCGKSCRLRWTNYLRPDI** | **KRGKFSLQEE** |
| 7. | CsMYB17 | 45 | 9.40e-28 | **GSWRALPAKA** | **GLQRCGKSCRLRWTNYLRPDI** | **KRGKFSLQEE** |
| 6. | CsMYB16 | 38 | 9.40e-28 | **GSWRLVPKKA** | **GLQRCGKSCRLRWTNYLRPDI** | **KRGKFTLQEE** |
| 70. | CsMYB76 | 45 | 1.99e-27 | **GKWRNLPKQA** | **GLQRCGKSCRLRWLNYLRPDI** | **KRGNISEEEE** |
| 51. | CsMYB61 | 45 | 3.64e-27 | **ANWRALPKQA** | **GLLRCGKSCRLRWINYLRPDI** | **KRGNFTAEEE** |
| 50. | CsMYB60 | 45 | 3.64e-27 | **GNWRALPKLA** | **GLLRCGKSCRLRWINYLRPDI** | **KRGNFTKEEE** |
| 15. | CsMYB25 | 46 | 3.64e-27 | **ANWRALPKQA** | **GLLRCGKSCRLRWINYLRPDI** | **KRGNFSKEEE** |
| 19. | CsMYB29 | 45 | 4.66e-27 | **GRWRTLPKNA** | **GLKRCGKSCRLRWTNYLRPDI** | **KRGRFSFEEE** |
| 93. | CsMYB97 | 45 | 5.72e-27 | **GCWSSVPKLA** | **GLQRCGKSCRLRWINYLRPDL** | **KRGPFSQQEE** |
| 92. | CsMYB96 | 45 | 5.72e-27 | **GCWSSVPKLA** | **GLQRCGKSCRLRWINYLRPDL** | **KRGPFSQQEE** |
| 88. | CsMYB93 | 57 | 5.72e-27 | **GCWSDVARNA** | **GLQRCGKSCRLRWINYLRPDL** | **KRGAFSPQEE** |
| 73. | CsMYB79 | 44 | 5.72e-27 | **GNWSSVPKLA** | **GLQRCGKSCRLRWINYLRPDL** | **RKGSFTQEEE** |
| 72. | CsMYB78 | 45 | 5.72e-27 | **GCWSSVPKLA** | **GLQRCGKSCRLRWINYLRPDL** | **KRGSFSPQEA** |
| 67. | CsMYB73 | 73 | 5.72e-27 | **GCWSDIARNA** | **GLQRCGKSCRLRWINYLRPDL** | **KRGAFSSHEQ** |
| 54. | CsMYB62 | 45 | 5.72e-27 | **GCWSSVPKLA** | **GLQRCGKSCRLRWINYLRPDL** | **KRGMFSQQEE** |
| 74. | CsMYB80 | 45 | 9.49e-27 | **GKWRSLPKRA** | **GLKRCGKSCRLRWLNYLRPDI** | **KRGNISPDEE** |
| 42. | CsMYB52 | 45 | 9.49e-27 | **GKWRDLPKRA** | **GLKRCGKSCRLRWLNYLRPDI** | **TRGNISTDED** |
| 41. | CsMYB51 | 45 | 9.49e-27 | **GKWRDLPKRA** | **GLKRCGKSCRLRWLNYLRPDI** | **TRGNISTDED** |
| 17. | CsMYB27 | 46 | 9.49e-27 | **GSWRSLPKLA** | **GLLRCGKSCRLRWTNYLRPDI** | **KRGPFTQDEE** |
| 68. | CsMYB74 | 70 | 1.40e-26 | **RNWRLIPKNA** | **GLQRCGKSCRLRWTNYLRPDL** | **KHGQFSDAEE** |
| 8. | CsMYB18 | 45 | 1.74e-26 | **GSWRALPKLA** | **GLNRCGKSCRLRWTNYLRPDI** | **KRGKFSEDEE** |
| 5. | CsMYB15 | 45 | 1.74e-26 | **GSWRALPRLA** | **GLNRCGKSCRLRWTNYLRPDI** | **KRGKFSQEEE** |
| 61. | CsMYB67 | 45 | 2.10e-26 | **GHWRIVPKKA** | **GLLRCGKSCRLRWMNYLRPDI** | **KRGNITADED** |
| 24. | CsMYB34 | 47 | 2.10e-26 | **ENWRALPKQA** | **GLLRCGKSCRLRWINYLRPDV** | **KRGNFSNEEE** |
| 78. | CsMYB84 | 45 | 2.61e-26 | **HCWRMVPKLA** | **GLLRCGKSCRLRWINYLRPDL** | **KRGGFTELEE** |
| 49. | CsMYB59 | 44 | 2.61e-26 | **GCWRTLPKAA** | **GLLRCGKSCRLRWINYLRPDL** | **KRGNFAEDEE** |
| 38. | CsMYB48 | 45 | 2.61e-26 | **GCWRSLPKAA** | **GLLRCGKSCRLRWINYLRPDL** | **KRGNFTEEED** |
| 37. | CsMYB47 | 46 | 2.61e-26 | **GNWIALPHKA** | **GLNRCGKSCRLRWLNYLRPDI** | **KHGGFTEEED** |
| 12. | CsMYB22 | 45 | 2.61e-26 | **GCWRSLPKAA** | **GLLRCGKSCRLRWINYLRPDL** | **KRGNFTEDED** |
| 11. | CsMYB21 | 45 | 2.61e-26 | **GCWRSLPKAA** | **GLLRCGKSCRLRWINYLRPDL** | **KRGNFTEDED** |
| 16. | CsMYB26 | 45 | 4.36e-26 | **CCWRAVPKLA** | **GLLRCGKSCRLRWTNYLRPDL** | **KRGLLSDYEE** |
| 77. | CsMYB83 | 45 | 6.70e-26 | **GNWTLVPKKA** | **GLNRCGKSCRLRWTNYLRPDL** | **KHDTFTPQEE** |
| 47. | CsMYB57 | 44 | 1.03e-25 | **GCWRSLPKAA** | **GLHRCGKSCRLRWINYLRPDI** | **KRGNFGEDEE** |
| 25. | CsMYB35 | 45 | 1.03e-25 | **CCWRAVPKLA** | **GLRRCGKSCRLRWTNYLRPDL** | **KRGLLTEAEE** |
| 14. | CsMYB24 | 45 | 1.03e-25 | **GTWRSLPKHA** | **GLLRCGKSCRLRWTNYLRPGI** | **KRGPFSQEEE** |
| 32. | CsMYB42 | 46 | 1.20e-25 | **GNWIALPQKI** | **GLKRCGKSCRLRWLNYLRPNI** | **KHGGFSEEED** |
| 31. | CsMYB41 | 46 | 1.20e-25 | **GNWIALPQKI** | **GLKRCGKSCRLRWLNYLRPNI** | **KHGGFSEEED** |
| 21. | CsMYB31 | 46 | 1.20e-25 | **GNWIALPQKA** | **GLKRCGKSCRLRWLNYLRPNI** | **KHGEFSDEED** |
| 39. | CsMYB49 | 45 | 2.10e-25 | **GGWRSLPQKA** | **GLSRCGKSCRLRWANYLRPDI** | **KRGAFSPEEE** |
| 29. | CsMYB39 | 46 | 2.46e-25 | **RRWKTLSIQS** | **GLNRCGKSCRLRWLNYLRPNI** | **KRGNISEAEE** |
| 59. | CsMYB65 | 45 | 4.61e-25 | **WNWRELPKFA** | **GLSRCGKSCRLRWMNYLRPDV** | **RRGNYTAEEE** |
| 28. | CsMYB38 | 45 | 4.61e-25 | **WNWRQLPKFA** | **GLKRCGKSCRLRWMNYLRPNV** | **KRGNYTHEEE** |
| 60. | CsMYB66 | 45 | 7.25e-25 | **GGWRSLPEKA** | **GLSRCGKSCRLRWTNYLRPGI** | **KRGEFTNEEE** |
| 33. | CsMYB43 | 46 | 1.11e-24 | **GNWIALPQKI** | **GLKRCGKSCRLRWLNYLRPHI** | **KHGGFSEEED** |
| 69. | CsMYB75 | 45 | 1.65e-24 | **GGWRHLPQKA** | **GLSRCGKSCRLRWANYLRPGI** | **KRGEFSPEEE** |
| 89. | CsMYB94 | 86 | 1.85e-24 | **GRWNSLARCA** | **GLKRTGKSCRLRWLNYLRPDV** | **RRGNITLEEQ** |
| 80. | CsMYB86 | 51 | 1.85e-24 | **GVWNSLAKAA** | **GLKRTGKSCRLRWLNYLRPDV** | **RRGNITNEEQ** |
| 26. | CsMYB36 | 46 | 2.40e-24 | **WNWTEMSKAA** | **GLNRSGKSCRLRWVNYLRPDI** | **KHGNFSQEEK** |
| 46. | CsMYB56 | 57 | 2.71e-24 | **GRWRTLPKRA** | **GLLRCGKSCRLRWMNYLRPSV** | **KRGHIQPDEE** |
| 43. | CsMYB53 | 45 | 2.71e-24 | **GSWRSLPKNA** | **GLLRCGKSCRLRWINYLRADL** | **KRGNITSEEE** |
| 27. | CsMYB37 | 45 | 2.71e-24 | **GSWRSLPKNA** | **GLLRCGKSCRLRWINYLRADL** | **KRGNISSEEE** |
| 13. | CsMYB23 | 48 | 4.92e-24 | **GSWKSLPKLA** | **GLIRCGKSCRLRWNNYLRPDI** | **KRGKFSEDEE** |
| 58. | CsMYB64 | 64 | 7.85e-24 | **QKWKTVAAIA** | **GLNRCGKSCRMRWLNYLRPNI** | **KRGNITDQEE** |
| 4. | CsMYB14 | 45 | 8.86e-24 | **GNWRAVPTNT** | **GLLRCSKSCRLRWTNYLRPGI** | **KRGNFTDNEE** |
| 3. | CsMYB13 | 45 | 8.86e-24 | **GNWRAVPTNT** | **GLLRCSKSCRLRWTNYLRPGI** | **KRGNFTDNEE** |
| 36. | CsMYB46 | 53 | 1.60e-23 | **GRWNSVARLA** | **GLKRNGKSCRLRWVNYLRPDL** | **KRGQITPHEE** |
| 66. | CsMYB72 | 65 | 1.80e-23 | **GNWNSVQKYS** | **GLNRCGKSCRLRWANHLRPNL** | **KKGSFTPEEE** |
| 64. | CsMYB70 | 20 | 1.80e-23 | **GNWNSVQKYS** | **GLNRCGKSCRLRWANHLRPNL** | **KKGSFTPEEE** |
| 62. | CsMYB68 | 65 | 1.80e-23 | **GNWNSVQKYS** | **GLNRCGKSCRLRWANHLRPNL** | **KKGSFTPEEE** |
| 87. | CsMYB92 | 49 | 2.02e-23 | **GHWNLLAKRA** | **GLKRTGKSCRLRWLNYLKPDI** | **KRGNLTPQEQ** |
| 86. | CsMYB91 | 49 | 2.02e-23 | **GHWNLLAKRA** | **GLKRTGKSCRLRWLNYLKPDI** | **KRGNLTPQEQ** |
| 2. | CsMYB12 | 45 | 2.02e-23 | **GNWRSVPTHT** | **GLMRCSKSCRLRWTNYLRPGI** | **KRGNFTDHEE** |
| 1. | CsMYB11 | 45 | 2.02e-23 | **GNWRSVPTNT** | **GLMRCSKSCRLRWTNYLRPGI** | **KRGNFTPHEE** |
| 34. | CsMYB44 | 46 | 2.53e-23 | **GCWSSVPIKA** | **GLQRNGKSCRLRWINYLRPGL** | **KRGTFSQHEE** |
| 75. | CsMYB81 | 45 | 2.83e-23 | **AKWTSVPKGA** | **GLRRCGKSCRLRWNNHLRPDL** | **NHESFTPKEE** |
| 10. | CsMYB20 | 51 | 3.16e-23 | **GRWSSVARSA** | **GLNRSGKSCRLRWVNYLRPGL** | **KRGQITPQEE** |
| 71. | CsMYB77 | 70 | 3.93e-23 | **GRWNSLARHA** | **GLKRTGKSCRLRWLNYLRPSV** | **RRGNITLQEQ** |
| 45. | CsMYB55 | 53 | 4.36e-23 | **KRWDALAKAS** | **GLRRSGKSCRLRWLNYLRPNL** | **KHDQISAEEE** |
| 44. | CsMYB54 | 41 | 4.36e-23 | **KRWDALAKAS** | **GLRRSGKSCRLRWLNYLRPNL** | **KHDQISAEEE** |
| 76. | CsMYB82 | 60 | 5.97e-23 | **GKWHLVPLRA** | **GLNRCRKSCRLRWLNYLKPDI** | **KRGEFTADEV** |
| 40. | CsMYB50 | 70 | 6.62e-23 | **GNWNAVQKNS** | **GLARCGKSCRLRWANHLRPNL** | **KKGSFSPDEE** |
| 57. | CsMYB63 | 56 | 9.94e-23 | **GRWNLLAKRS** | **GLRRTGKSCRLRWLNYLKPDV** | **KRGNLTPEEQ** |
| 81. | CsMYB87 | 46 | 1.64e-22 | **GKWHLVPLRA** | **GLSRCRKSCRLRWLNYLKPDI** | **KRGEFEEDEV** |
| 35. | CsMYB45 | 45 | 3.60e-22 | **GNWQTVSKLT** | **GLKRGGKSCRLRWKNYLRPNI** | **KRGGMSQEEE** |
| 53. | CsMYB2 | 73 | 6.35e-22 | **LFLFVMFIFV** | **GLNRTGKSCRLRWVNYLHPGL** | **KRGKMTPQEE** |
| 52. | CsMYB1 | 40 | 6.35e-22 | **FEGGGRQNKI** | **GLNRTGKSCRLRWVNYLHPGL** | **KRGKMTPQEE** |
| 48. | CsMYB58 | 42 | 3.78e-21 | **RDWSSIRSKG** | **LLQRTGKSCRLRWVNKLRPNL** | **KNGCKFSLEE** |
| 30. | CsMYB40 | 43 | 8.81e-21 | **RDWSSIRSKG** | **LLPRTGKSCRLRWVNKLRPDL** | **KTGCKFSAEE** |
| 65. | CsMYB71 | 91 | 8.26e-20 | **ARNWSLIARG** | **IPGRSGKSCRLRWCNQLDPCV** | **KRKPFTEEED** |
| 63. | CsMYB69 | 87 | 8.26e-20 | **ARNWSLIARG** | **IPGRSGKSCRLRWCNQLDPCV** | **KRKPFTEEED** |
| 84. | CsMYB90 | 43 | 1.77e-19 | **PRNWSLISKS** | **IPGRSGKSCRLRWCNQLSPQV** | **EHRAFTAEED** |
| 83. | CsMYB89 | 43 | 1.77e-19 | **PRNWSLISKS** | **IPGRSGKSCRLRWCNQLSPQV** | **EHRAFTAEED** |
| 79. | CsMYB85 | 40 | 1.77e-19 | **ARNWSVISKS** | **IPGRSGKSCRLRWCNQLSPEV** | **KHRPFTDMED** |
| 82. | CsMYB88 | 38 | 4.00e-19 | **PRNWSVISTG** | **IIGRSGKSCRLRWCNQLCPSV** | **QHRPFTPEED** |
| 104. | CsMYB98 | 53 | 5.36e-19 | **PQNWNFIAEH** | **LQGRSGKSCRLRWYNQLDPNI** | **NKKPFTEEEE** |
| 91. | CsMYB95 | 71 | 5.36e-19 | **ARNWSLISRY** | **IKGRSGKSCRLRWCNQLSPSV** | **EHRPFSPAED** |
| 95. | CsMYB104 | 124 | 1.69e-18 | **AKKWSMIAEA** | **LPGRIGKQCRERWHNHLNPAI** | **NKEPWTQEEE** |
| 94. | CsMYB103 | 124 | 1.69e-18 | **AKKWSMIAEA** | **LPGRIGKQCRERWHNHLNPAI** | **NKEPWTQEEE** |
| 85. | CsMYB101 | 115 | 1.69e-18 | **PKKWSTIANH** | **LPGRIGKQCRERWHNHLNPAI** | **NKEAWTQEEE** |
| 56. | CsMYB100 | 157 | 1.94e-18 | **ATKWSLIAKS** | **LPGRIGKQCRERWHNHLNPEI** | **RKDAWTIAEE** |
| 55. | CsMYB99 | 141 | 1.94e-18 | **ATKWSLIAKS** | **LPGRIGKQCRERWHNHLNPEI** | **RKDAWTIAEE** |
| 90. | CsMYB102 | 109 | 1.01e-17 | **VKRWSTIAKF** | **IKGRMGKQCRERWHNHLDPSL** | **NKNPWTEEEE** |

MEME Motif 4 E-value: 1.4e-298 Site Count: 50 Width: 11


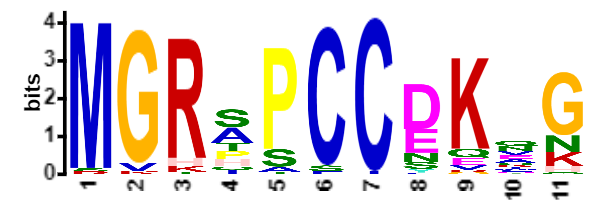


| 20. | CsMYB30 | 1 | 6.72e-15 |  | **MGRAPCCDKNG** | **LKKGPWTPEE** |
| --- | --- | --- | --- | --- | --- | --- |
| 18. | CsMYB28 | 1 | 6.72e-15 |  | **MGRAPCCDKNG** | **LKKGPWTPEE** |
| 9. | CsMYB19 | 1 | 8.60e-15 |  | **MGRSPCCDKVG** | **LKKGPWTPEE** |
| 2. | CsMYB12 | 1 | 8.60e-15 |  | **MGRPPCCDKVG** | **VKKGPWTPEE** |
| 1. | CsMYB11 | 1 | 8.60e-15 |  | **MGRPPCCDKVG** | **IKKGPWTPEE** |
| 51. | CsMYB61 | 1 | 2.18e-14 |  | **MGRAPCCEKMG** | **LKKGPWTPEE** |
| 50. | CsMYB60 | 1 | 2.18e-14 |  | **MGRAPCCEKMG** | **LKKGPWTPEE** |
| 16. | CsMYB26 | 1 | 2.18e-14 |  | **MGRQPCCDKVG** | **LKKGPWTAEE** |
| 4. | CsMYB14 | 1 | 2.18e-14 |  | **MGRPPCCDKEG** | **VKKGPWTPEE** |
| 3. | CsMYB13 | 1 | 2.18e-14 |  | **MGRPPCCDKEG** | **VKKGPWTPEE** |
| 7. | CsMYB17 | 1 | 2.84e-14 |  | **MGRSPCCEKVG** | **LKKGPWTPEE** |
| 27. | CsMYB37 | 1 | 4.03e-14 |  | **MGRAPCCEKIG** | **LKKGRWTSEE** |
| 33. | CsMYB43 | 1 | 5.89e-14 |  | **MGRAPCCDKAN** | **VKKGPWSPEE** |
| 32. | CsMYB42 | 1 | 5.89e-14 |  | **MGRAPCCDKAN** | **VKKGPWSVEE** |
| 31. | CsMYB41 | 1 | 5.89e-14 |  | **MGRAPCCDKAN** | **VKKGPWSPEE** |
| 17. | CsMYB27 | 1 | 6.46e-14 |  | **MGRTPCCDKKG** | **LKKGPWTAEE** |
| 78. | CsMYB84 | 1 | 9.36e-14 |  | **MGRQPCCDKIG** | **LKRGPWTIEE** |
| 43. | CsMYB53 | 1 | 1.24e-13 |  | **MGRAPCCEKLG** | **LKKGRWTAEE** |
| 21. | CsMYB31 | 1 | 1.74e-13 |  | **MGRSPCCDKAN** | **VKKGPWSPEE** |
| 42. | CsMYB52 | 1 | 2.12e-13 |  | **MGRSPCCEKDG** | **LNRGAWSALE** |
| 41. | CsMYB51 | 1 | 2.12e-13 |  | **MGRSPCCEKDG** | **LNRGAWSALE** |
| 25. | CsMYB35 | 1 | 2.67e-13 |  | **MGRQPCCDKLG** | **VKKGPWTAEE** |
| 37. | CsMYB47 | 1 | 4.24e-13 |  | **MGRAPCCDKTK** | **VKRGPWSPEE** |
| 38. | CsMYB48 | 1 | 4.69e-13 |  | **MGRSPCCEKAH** | **TNKGAWTKEE** |
| 74. | CsMYB80 | 1 | 5.66e-13 |  | **MGRSPCCSKEG** | **LNRGAWTAME** |
| 12. | CsMYB22 | 1 | 6.16e-13 |  | **MGRSPCCEKEH** | **TNKGAWTKEE** |
| 11. | CsMYB21 | 1 | 6.16e-13 |  | **MGRSPCCEKEH** | **TNKGAWTKEE** |
| 69. | CsMYB75 | 1 | 6.86e-13 |  | **MGRTPCCDQKG** | **LRKGAWTAEE** |
| 77. | CsMYB83 | 1 | 9.23e-13 |  | **MGRPPCCDKSN** | **VKRGLWTAEE** |
| 70. | CsMYB76 | 1 | 9.23e-13 |  | **MGRSPCCSKKG** | **LNKGAWTVLE** |
| 68. | CsMYB74 | 26 | 1.12e-12 | **VDQASCGLEK** | **MGRIPCCEKEN** | **VKRGQWTPEE** |
| 5. | CsMYB15 | 1 | 2.24e-12 |  | **MGRSPCCDESG** | **LKKGPWTPEE** |
| 39. | CsMYB49 | 1 | 2.45e-12 |  | **MGRSPCCNVQG** | **LKKGAWTPEE** |
| 60. | CsMYB66 | 1 | 3.15e-12 |  | **MGRTPCCNVQG** | **LKKGAWTTEE** |
| 93. | CsMYB97 | 1 | 5.22e-12 |  | **MGRHSCCYKQK** | **LRKGLWSPEE** |
| 92. | CsMYB96 | 1 | 5.22e-12 |  | **MGRHSCCYKQK** | **LRKGLWSPEE** |
| 28. | CsMYB38 | 1 | 6.71e-12 |  | **MVRTPCCDQNG** | **MKKGTWTPEE** |
| 13. | CsMYB23 | 1 | 1.55e-11 |  | **MGRSPCCDGIN** | **IGLKKGPWTP** |
| 54. | CsMYB62 | 1 | 3.22e-11 |  | **MGRHSCCLKQK** | **LRKGLWSPEE** |
| 14. | CsMYB24 | 1 | 4.82e-11 |  | **MGKIACCDKNG** | **VKKGAWTPEE** |
| 72. | CsMYB78 | 1 | 1.35e-10 |  | **MGHHSCCNKQK** | **VKRGLWSPEE** |
| 19. | CsMYB29 | 1 | 2.13e-10 |  | **MGKITCCEKNG** | **LKKGPWTPEE** |
| 24. | CsMYB34 | 3 | 2.87e-10 | **MG** | **RGRAPCCDKSQ** | **VKRGPWSPAE** |
| 8. | CsMYB18 | 1 | 3.10e-10 |  | **MGRSPSCDENG** | **LKKGPWTPEE** |
| 61. | CsMYB67 | 1 | 3.59e-10 |  | **MGRTACCSRFG** | **LRRGPWTPRE** |
| 23. | CsMYB33 | 1 | 6.38e-10 |  | **MGHHSCCNQQK** | **VKRGLWSPEE** |
| 22. | CsMYB32 | 1 | 6.38e-10 |  | **MGHHSCCNQQK** | **VKRGLWSPEE** |
| 75. | CsMYB81 | 1 | 8.60e-9 |  | **MVRPSSCEKAN** | **LKGGLWSEEE** |
| 59. | CsMYB65 | 1 | 6.23e-8 |  | **MVRAPFIDENG** | **VKRGAWSPEE** |
| 46. | CsMYB56 | 13 | 1.08e-7 | **RGSSSSSGGG** | **SKTTPCCSKVG** | **IKRGPWTAEE** |

MEME Motif 5 E-value: 1.3e-220 Site Count: 75 Width: 9


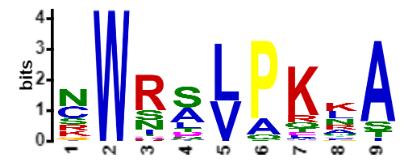


| 25. | CsMYB35 | 36 | 1.88e-12 | **INFILTNGHC** | **CWRAVPKLA** | **GLRRCGKSCR** |
| --- | --- | --- | --- | --- | --- | --- |
| 16. | CsMYB26 | 36 | 1.88e-12 | **INFILTNGQC** | **CWRAVPKLA** | **GLLRCGKSCR** |
| 47. | CsMYB57 | 35 | 1.29e-11 | **IDYITTHGEG** | **CWRSLPKAA** | **GLHRCGKSCR** |
| 38. | CsMYB48 | 36 | 1.29e-11 | **IAYIRAHGEG** | **CWRSLPKAA** | **GLLRCGKSCR** |
| 12. | CsMYB22 | 36 | 1.29e-11 | **IHYIKLHGEG** | **CWRSLPKAA** | **GLLRCGKSCR** |
| 11. | CsMYB21 | 36 | 1.29e-11 | **IHYIKLHGEG** | **CWRSLPKAA** | **GLLRCGKSCR** |
| 50. | CsMYB60 | 36 | 5.73e-11 | **ISYIHKYGHG** | **NWRALPKLA** | **GLLRCGKSCR** |
| 51. | CsMYB61 | 36 | 1.26e-10 | **INYVQKFGHA** | **NWRALPKQA** | **GLLRCGKSCR** |
| 24. | CsMYB34 | 38 | 1.26e-10 | **ITFIQKNGHE** | **NWRALPKQA** | **GLLRCGKSCR** |
| 15. | CsMYB25 | 37 | 1.26e-10 | **ISYIQQFGHA** | **NWRALPKQA** | **GLLRCGKSCR** |
| 49. | CsMYB59 | 35 | 1.56e-10 | **IDYINQHGEG** | **CWRTLPKAA** | **GLLRCGKSCR** |
| 78. | CsMYB84 | 36 | 1.87e-10 | **MNFILNNGIH** | **CWRMVPKLA** | **GLLRCGKSCR** |
| 93. | CsMYB97 | 36 | 3.64e-10 | **LNYITKHGHG** | **CWSSVPKLA** | **GLQRCGKSCR** |
| 92. | CsMYB96 | 36 | 3.64e-10 | **LNYITKHGHG** | **CWSSVPKLA** | **GLQRCGKSCR** |
| 74. | CsMYB80 | 36 | 3.64e-10 | **SEYIKVHGEG** | **KWRSLPKRA** | **GLKRCGKSCR** |
| 72. | CsMYB78 | 36 | 3.64e-10 | **INYISTYGHG** | **CWSSVPKLA** | **GLQRCGKSCR** |
| 54. | CsMYB62 | 36 | 3.64e-10 | **FNYITHFGVG** | **CWSSVPKLA** | **GLQRCGKSCR** |
| 8. | CsMYB18 | 36 | 3.64e-10 | **VDYITRNGHG** | **SWRALPKLA** | **GLNRCGKSCR** |
| 17. | CsMYB27 | 37 | 7.97e-10 | **EYIKKNNGHG** | **SWRSLPKLA** | **GLLRCGKSCR** |
| 6. | CsMYB16 | 29 | 7.97e-10 | **LAYIQQHGHG** | **SWRLVPKKA** | **GLQRCGKSCR** |
| 43. | CsMYB53 | 36 | 1.07e-9 | **TNYIQTHGEG** | **SWRSLPKNA** | **GLLRCGKSCR** |
| 27. | CsMYB37 | 36 | 1.07e-9 | **TKYIQSNGEG** | **SWRSLPKNA** | **GLLRCGKSCR** |
| 46. | CsMYB56 | 48 | 1.25e-9 | **CEFIRKEGEG** | **RWRTLPKRA** | **GLLRCGKSCR** |
| 20. | CsMYB30 | 36 | 1.25e-9 | **VDYIQKHGYG** | **NWRTLPKNA** | **GLQRCGKSCR** |
| 73. | CsMYB79 | 35 | 1.70e-9 | **LTHITTHGHG** | **NWSSVPKLA** | **GLQRCGKSCR** |
| 19. | CsMYB29 | 36 | 1.95e-9 | **IDHIKRNGHG** | **RWRTLPKNA** | **GLKRCGKSCR** |
| 42. | CsMYB52 | 36 | 2.27e-9 | **ADYIHKNGPG** | **KWRDLPKRA** | **GLKRCGKSCR** |
| 41. | CsMYB51 | 36 | 2.27e-9 | **ADYIHKNGPG** | **KWRDLPKRA** | **GLKRCGKSCR** |
| 59. | CsMYB65 | 36 | 3.96e-9 | **RAYVERYGHW** | **NWRELPKFA** | **GLSRCGKSCR** |
| 5. | CsMYB15 | 36 | 3.96e-9 | **VKFIQKNGHG** | **SWRALPRLA** | **GLNRCGKSCR** |
| 18. | CsMYB28 | 36 | 5.15e-9 | **INYIQKNGPG** | **NWRNLPKNA** | **GLQRCGKSCR** |
| 61. | CsMYB67 | 36 | 5.91e-9 | **VNYIQLHGEG** | **HWRIVPKKA** | **GLLRCGKSCR** |
| 21. | CsMYB31 | 37 | 6.70e-9 | **EYIEKYGTGG** | **NWIALPQKA** | **GLKRCGKSCR** |
| 39. | CsMYB49 | 36 | 7.61e-9 | **LSYIQQHGEG** | **GWRSLPQKA** | **GLSRCGKSCR** |
| 28. | CsMYB38 | 36 | 7.61e-9 | **VAYVTRYGCW** | **NWRQLPKFA** | **GLKRCGKSCR** |
| 9. | CsMYB19 | 36 | 7.61e-9 | **LAYIEQHGHG** | **SWRALPAKA** | **GLQRCGKSCR** |
| 7. | CsMYB17 | 36 | 7.61e-9 | **LAYIEEHGHG** | **SWRALPAKA** | **GLQRCGKSCR** |
| 77. | CsMYB83 | 36 | 1.11e-8 | **LAHVSKLGIG** | **NWTLVPKKA** | **GLNRCGKSCR** |
| 70. | CsMYB76 | 36 | 1.40e-8 | **TDYIKTHGEG** | **KWRNLPKQA** | **GLQRCGKSCR** |
| 14. | CsMYB24 | 36 | 1.40e-8 | **VNYIKRHGHG** | **TWRSLPKHA** | **GLLRCGKSCR** |
| 68. | CsMYB74 | 61 | 1.57e-8 | **SSYIAQHGTR** | **NWRLIPKNA** | **GLQRCGKSCR** |
| 34. | CsMYB44 | 37 | 1.98e-8 | **RNFVLKHGHG** | **CWSSVPIKA** | **GLQRNGKSCR** |
| 60. | CsMYB66 | 36 | 2.22e-8 | **SAYITQHGEG** | **GWRSLPEKA** | **GLSRCGKSCR** |
| 23. | CsMYB33 | 36 | 3.10e-8 | **IRYITTHGYG** | **CWSEVPEKA** | **GLQRCGKSCR** |
| 22. | CsMYB32 | 36 | 3.10e-8 | **IRYITTHGYG** | **CWSEVPEKA** | **GLQRCGKSCR** |
| 37. | CsMYB47 | 37 | 3.86e-8 | **HYMHNNGTGG** | **NWIALPHKA** | **GLNRCGKSCR** |
| 13. | CsMYB23 | 39 | 3.86e-8 | **DYIERNGHHG** | **SWKSLPKLA** | **GLIRCGKSCR** |
| 4. | CsMYB14 | 36 | 5.31e-8 | **VSYIQEHGPG** | **NWRAVPTNT** | **GLLRCSKSCR** |
| 3. | CsMYB13 | 36 | 5.31e-8 | **VSYIQEHGPG** | **NWRAVPTNT** | **GLLRCSKSCR** |
| 69. | CsMYB75 | 36 | 6.53e-8 | **FSYIKQHGEG** | **GWRHLPQKA** | **GLSRCGKSCR** |
| 36. | CsMYB46 | 44 | 7.23e-8 | **IEYVRLHGEG** | **RWNSVARLA** | **GLKRNGKSCR** |
| 1. | CsMYB11 | 36 | 8.84e-8 | **VSYIQEHGPG** | **NWRSVPTNT** | **GLMRCSKSCR** |
| 88. | CsMYB93 | 48 | 9.74e-8 | **MNYMLNNGQG** | **CWSDVARNA** | **GLQRCGKSCR** |
| 87. | CsMYB92 | 40 | 1.08e-7 | **IHYISLHGEG** | **HWNLLAKRA** | **GLKRTGKSCR** |
| 86. | CsMYB91 | 40 | 1.08e-7 | **IHYISLHGEG** | **HWNLLAKRA** | **GLKRTGKSCR** |
| 80. | CsMYB86 | 42 | 1.08e-7 | **INYIANHGEG** | **VWNSLAKAA** | **GLKRTGKSCR** |
| 75. | CsMYB81 | 36 | 1.08e-7 | **LEYVSKHGKA** | **KWTSVPKGA** | **GLRRCGKSCR** |
| 33. | CsMYB43 | 37 | 1.44e-7 | **AYIEQNGTGG** | **NWIALPQKI** | **GLKRCGKSCR** |
| 32. | CsMYB42 | 37 | 1.44e-7 | **SYIENHGTGG** | **NWIALPQKI** | **GLKRCGKSCR** |
| 31. | CsMYB41 | 37 | 1.44e-7 | **AYIDTYGTGG** | **NWIALPQKI** | **GLKRCGKSCR** |
| 2. | CsMYB12 | 36 | 1.44e-7 | **VSYIQEHGPG** | **NWRSVPTHT** | **GLMRCSKSCR** |
| 71. | CsMYB77 | 61 | 2.09e-7 | **FNYVSIHGEG** | **RWNSLARHA** | **GLKRTGKSCR** |
| 89. | CsMYB94 | 77 | 2.98e-7 | **INYIAIHGEG** | **RWNSLARCA** | **GLKRTGKSCR** |
| 40. | CsMYB50 | 61 | 2.98e-7 | **VEYVRKHGEG** | **NWNAVQKNS** | **GLARCGKSCR** |
| 45. | CsMYB55 | 44 | 4.21e-7 | **ISFVQLMGEK** | **RWDALAKAS** | **GLRRSGKSCR** |
| 44. | CsMYB54 | 32 | 4.21e-7 | **ISFVQLMGEK** | **RWDALAKAS** | **GLRRSGKSCR** |
| 81. | CsMYB87 | 37 | 4.58e-7 | **RECIDKYGEG** | **KWHLVPLRA** | **GLSRCRKSCR** |
| 76. | CsMYB82 | 51 | 4.58e-7 | **RDCVDKYGEG** | **KWHLVPLRA** | **GLNRCRKSCR** |
| 66. | CsMYB72 | 56 | 4.58e-7 | **MEYVKKHGEG** | **NWNSVQKYS** | **GLNRCGKSCR** |
| 64. | CsMYB70 | 11 | 4.58e-7 | **MEYVKKHGEG** | **NWNSVQKYS** | **GLNRCGKSCR** |
| 62. | CsMYB68 | 56 | 4.58e-7 | **MEYVKKHGEG** | **NWNSVQKYS** | **GLNRCGKSCR** |
| 57. | CsMYB63 | 47 | 5.89e-7 | **IHYITRHGEG** | **RWNLLAKRS** | **GLRRTGKSCR** |
| 10. | CsMYB20 | 42 | 6.39e-7 | **TEYVSFNGEG** | **RWSSVARSA** | **GLNRSGKSCR** |
| 67. | CsMYB73 | 64 | 8.82e-7 | **MRYMLRNGQG** | **CWSDIARNA** | **GLQRCGKSCR** |
| 35. | CsMYB45 | 36 | 7.60e-6 | **KNYVETHGEG** | **NWQTVSKLT** | **GLKRGGKSCR** |

**MEME Analysis II**

**Query: R1R2R3-CsMYBs amino acid sequences**

Motif 2 Light blue boxes (Logo sequence of R1 repeat) E-value: 8.9e-156 Site Count: 6 Width: 50


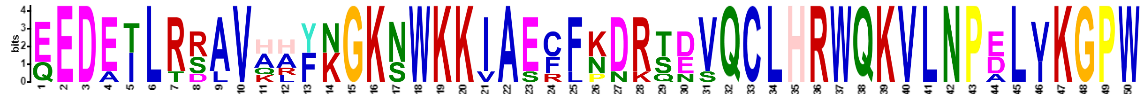


| 6. | CsMYB104 | 48 | 2.86e-65 | **RRSTTGKWTA** | **EEDEILRRAVHHYKGKNWKKIAECFKDRTDVQCLHRWQKVLNPELIKGPW** | **SKEEDEVIVE** |
| --- | --- | --- | --- | --- | --- | --- |
| 5. | CsMYB103 | 48 | 2.86e-65 | **RRSTTGKWTA** | **EEDEILRRAVHHYKGKNWKKIAECFKDRTDVQCLHRWQKVLNPELIKGPW** | **SKEEDEVIVE** |
| 3. | CsMYB101 | 39 | 7.17e-63 | **RRSTKGQWTP** | **EEDETLRRAVQRFKGKNWKKIAECFKDRTDVQCLHRWQKVLNPELVKGPW** | **SKEEDDVIVE** |
| 2. | CsMYB100 | 81 | 3.13e-57 | **VRRAKGGWTP** | **QEDETLRSAVAAFNGKSWKKIAEFFNDRSEVQCLHRWQKVLNPDLVKGPW** | **TPEEDKKITE** |
| 1. | CsMYB99 | 65 | 3.13e-57 | **VRRAKGGWTP** | **QEDETLRSAVAAFNGKSWKKIAEFFNDRSEVQCLHRWQKVLNPDLVKGPW** | **TPEEDKKITE** |
| 4. | CsMYB102 | 33 | 3.49e-43 | **GSIRTSTWTK** | **EEDAILTDLVKLYNGKNWKKVASRLPNKQNSQCLHRWQKVLNPALVKGPW** | **EKEEDKQLIK** |

Motif 4 Purple boxes (Logo sequence of R2 repeat) E-value: 2.4e-057 Site Count: 6 Width: 28


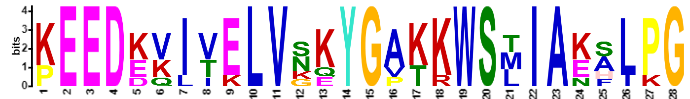


| 6. | CsMYB104 | 99 | 2.13e-35 | **NPELIKGPWS** | **KEEDEVIVELVNKYGAKKWSMIAEALPG** | **RIGKQCRERW** |
| --- | --- | --- | --- | --- | --- | --- |
| 5. | CsMYB103 | 99 | 2.13e-35 | **NPELIKGPWS** | **KEEDEVIVELVNKYGAKKWSMIAEALPG** | **RIGKQCRERW** |
| 2. | CsMYB100 | 132 | 6.59e-30 | **NPDLVKGPWT** | **PEEDKKITELVSKYGATKWSLIAKSLPG** | **RIGKQCRERW** |
| 1. | CsMYB99 | 116 | 6.59e-30 | **NPDLVKGPWT** | **PEEDKKITELVSKYGATKWSLIAKSLPG** | **RIGKQCRERW** |
| 3. | CsMYB101 | 90 | 1.35e-29 | **NPELVKGPWS** | **KEEDDVIVELVKQYGPKKWSTIANHLPG** | **RIGKQCRERW** |
| 4. | CsMYB102 | 84 | 2.93e-23 | **NPALVKGPWE** | **KEEDKQLIKLVGEYGVKRWSTIAKFIKG** | **RMGKQCRERW** |

Motif 1 Red boxes (Logo sequence of R3 repeat) E-value: 8.1e-173 Site Count: 6 Width: 50


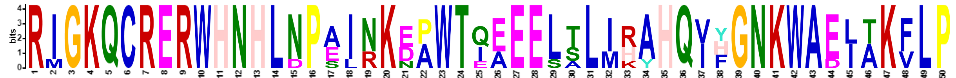


| 6. | CsMYB104 | 127 | 2.06e-64 | **WSMIAEALPG** | **RIGKQCRERWHNHLNPAINKEPWTQEEELTLIRAHQVFGNKWAELTKFLP** | **GRADNAIKNH** |
| --- | --- | --- | --- | --- | --- | --- |
| 5. | CsMYB103 | 127 | 2.06e-64 | **WSMIAEALPG** | **RIGKQCRERWHNHLNPAINKEPWTQEEELTLIRAHQVFGNKWAELTKFLP** | **GRADNAIKNH** |
| 3. | CsMYB101 | 118 | 1.87e-63 | **WSTIANHLPG** | **RIGKQCRERWHNHLNPAINKEAWTQEEELALIRAHQVYGNKWAELTKFLP** | **GRTDNSIKNH** |
| 2. | CsMYB100 | 160 | 3.09e-59 | **WSLIAKSLPG** | **RIGKQCRERWHNHLNPEIRKDAWTIAEELSLMHAHQIHGNKWAEIAKVLP** | **GRTDNAIKNH** |
| 1. | CsMYB99 | 144 | 3.09e-59 | **WSLIAKSLPG** | **RIGKQCRERWHNHLNPEIRKDAWTIAEELSLMHAHQIHGNKWAEIAKVLP** | **GRTDNAIKNH** |
| 4. | CsMYB102 | 112 | 1.24e-52 | **WSTIAKFIKG** | **RMGKQCRERWHNHLDPSLNKNPWTEEEESTLIKYHQIYGNKWADIAKFLP** | **GRSDNAIKNH** |

Motif 3 Green boxes E-value: 4.7e-058 Site Count: 6 Width: 29


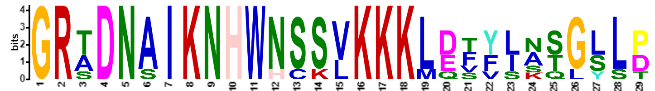


| 6. | CsMYB104 | 177 | 1.34e-33 | **KWAELTKFLP** | **GRADNAIKNHWNSSVKKKLETYLASGLLD** | **QFQGLPNVGN** |
| --- | --- | --- | --- | --- | --- | --- |
| 5. | CsMYB103 | 177 | 1.34e-33 | **KWAELTKFLP** | **GRADNAIKNHWNSSVKKKLETYLASGLLD** | **QFQGLPNVGN** |
| 2. | CsMYB100 | 210 | 2.36e-32 | **KWAEIAKVLP** | **GRTDNAIKNHWNSSLKKKLDFFINTGSLP** | **PVSKSNYQNG** |
| 1. | CsMYB99 | 194 | 2.36e-32 | **KWAEIAKVLP** | **GRTDNAIKNHWNSSLKKKLDFFINTGSLP** | **PVSKSNYQNG** |
| 3. | CsMYB101 | 168 | 2.09e-30 | **KWAELTKFLP** | **GRTDNSIKNHWNSSVKKKLDSYLKSGLLT** | **QFQGLPQVGQ** |
| 4. | CsMYB102 | 162 | 1.18e-22 | **KWADIAKFLP** | **GRSDNAIKNHWHCKVKKKMQVVSSQLYSP** | **VVLHGTNNSN** |

Motif 5 Orange boxes E-value: 6.5e-028 Site Count: 2 Width: 50


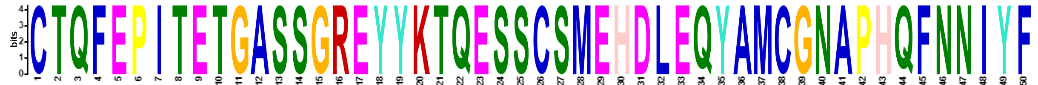


| 6. | CsMYB104 | 248 | 6.31e-67 | **ECSQSSNGVN** | **CTQFEPITETGASSGREYYKTQESSCSMEHDLEQYAMCGNAPHQFNNIYF** | **GSHKLSRMAH** |
| --- | --- | --- | --- | --- | --- | --- |
| 5. | CsMYB103 | 248 | 6.31e-67 | **ECSQSSNGVN** | **CTQFEPITETGASSGREYYKTQESSCSMEHDLEQYAMCGNAPHQFNNIYF** | **GSHKLSRMAH** |

**MEME Analysis III**

**Query: R3-CsMYBs amino acid sequences**

Motif 1 Red boxes (Logo sequence of R3 repeat) E-value: 3.4e-221 Site Count: 10 Width: 44


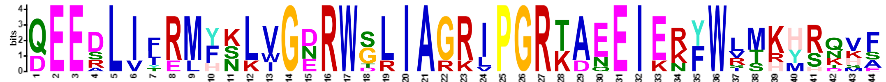


| 7. | CsMYB7 | 51 | 4.11e-55 | **EWEFIHMSEE** | **QEEDLIFRMYKLVGDRWGLIAGRIPGRKAEEIERFWLMKHRQVF** | **DK** |
| --- | --- | --- | --- | --- | --- | --- |
| 6. | CsMYB6 | 30 | 4.11e-55 | **IEWEFIHMSE** | **QEEDLIFRMYKLVGDRWGLIAGRIPGRKAEEIERFWLMKHRQVF** | **DK** |
| 4. | CsMYB4 | 50 | 4.11e-55 | **EWEFIHMSEE** | **QEEDLIFRMYKLVGDRWGLIAGRIPGRKAEEIERFWLMKHRQVF** | **DK** |
| 3. | CsMYB3 | 29 | 4.11e-55 | **IEWEFIHMSE** | **QEEDLIFRMYKLVGDRWGLIAGRIPGRKAEEIERFWLMKHRQVF** | **DK** |
| 5. | CsMYB5 | 39 | 9.52e-45 | **LEWEFINMTE** | **QEEDLIYRMFSLVGNRWDLIAGRVPGRTAQEIERFWIMRHHDMF** | **AERRKNQTK** |
| 9. | CsMYB9 | 41 | 2.24e-43 | **REVVEVEFSE** | **DEESLIIRMFNLIGERWSLIAGRIPGRTAEEIEKYWVSKYSNHS** | **TKTNNNNQQL** |
| 8. | CsMYB8 | 40 | 2.24e-43 | **REVVEVEFSE** | **DEESLIIRMFNLIGERWSLIAGRIPGRTAEEIEKYWVSKYSNHS** | **TKTNNNNQQL** |
| 10. | CsMYB10 | 35 | 5.64e-41 | **KLEFKLDFSE** | **DEEALITRMFNLVGERWTLIAGRIPGRTAEEIEKYWSTRYSTSE** |  |
| 2. | CsMYB2 | 101 | 1.02e-34 | **PGLKRGKMTP** | **QEERLVLELHSKWGNRWSRIARKLPGRTDNEIKNYWRTHMRKKA** | **QEKKKSTTTT** |
| 1. | CsMYB1 | 68 | 1.02e-34 | **PGLKRGKMTP** | **QEERLVLELHSKWGNRWSRIARKLPGRTDNEIKNYWRTHMRKKA** | **QEKKKSTTTT** |

Motif 2 Light blue boxes E-value: 3.4e-034 Site Count: 9 Width: 15


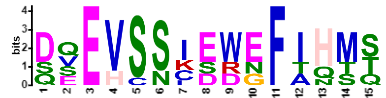


| 7. | CsMYB7 | 34 | 4.75e-17 | **EPNNCISPCS** | **QVEVSSIEWEFIHMS** | **EEQEEDLIFR** |
| --- | --- | --- | --- | --- | --- | --- |
| 6. | CsMYB6 | 14 | 4.75e-17 | **PTNNCISPCS** | **QVEVSSIEWEFIHMS** | **EQEEDLIFRM** |
| 4. | CsMYB4 | 33 | 7.89e-17 | **EEPNNCISPC** | **SQEVSSIEWEFIHMS** | **EEQEEDLIFR** |
| 3. | CsMYB3 | 13 | 7.89e-17 | **NPTNNCISPC** | **SQEVSSIEWEFIHMS** | **EQEEDLIFRM** |
| 5. | CsMYB5 | 23 | 1.19e-14 | **QAKHPHNFES** | **DQEVCSLEWEFINMT** | **EQEEDLIYRM** |
| 9. | CsMYB9 | 3 | 1.50e-13 | **MA** | **DSEHSSCDDNFAHSQ** | **AEEEEVMSEE** |
| 8. | CsMYB8 | 3 | 1.50e-13 | **MA** | **DSEHSSCDDNFAHSQ** | **AEEEVMSEES** |
| 2. | CsMYB2 | 299 | 8.57e-12 | **CSDSLWINNL** | **DEEVSNKSRGFTQTT** | **ATAINDPFNY** |
| 1. | CsMYB1 | 266 | 8.57e-12 | **CSDSLWINNL** | **DEEVSNKSRGFTQTT** | **ATAINDPFNY** |

Motif 3 Green boxes E-value:  6.6e-031 Site Count: 2 Width: 15


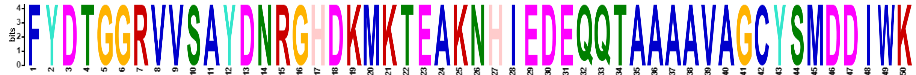


| 2. | CsMYB2 | 204 | 6.26e-67 | **FPDSTTGVES** | **FYDTGGRVVSAYDNRGHDKMKTEAKNHIEDEQQTAAAAVAGCYSMDDIWK** | **EISLPEEGVY** |
| --- | --- | --- | --- | --- | --- | --- |
| 1. | CsMYB1 | 171 | 6.26e-67 | **FPDSTTGVES** | **FYDTGGRVVSAYDNRGHDKMKTEAKNHIEDEQQTAAAAVAGCYSMDDIWK** | **EISLPEEGVY** |

Motif 4 Purple boxes E-value: 9.3e-022 Site Count: 2 Width: 41


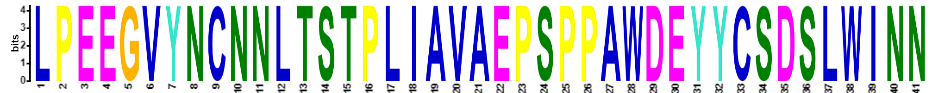


| 2. | CsMYB2 | 257 | 4.51e-54 | **SMDDIWKEIS** | **LPEEGVYNCNNLTSTPLIAVAEPSPPAWDEYYCSDSLWINN** | **LDEEVSNKSR** |
| --- | --- | --- | --- | --- | --- | --- |
| 1. | CsMYB1 | 224 | 4.51e-54 | **SMDDIWKEIS** | **LPEEGVYNCNNLTSTPLIAVAEPSPPAWDEYYCSDSLWINN** | **LDEEVSNKSR** |

Motif 5 Orange boxes E-value: 6.1e-015 Site Count: 2 Width: 40


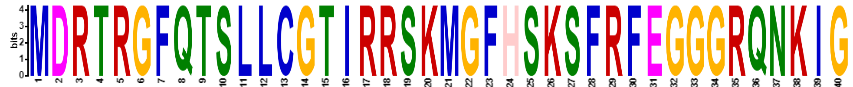


| 2. | CsMYB2 | 1 | 6.08e-51 |  | **MDRTRGFQTSLLCGTIRRSKMGFHSKSFRFEGGGRQNKIG** | **MVIRRWRIIQ** |
| --- | --- | --- | --- | --- | --- | --- |
| 1. | CsMYB1 | 1 | 6.08e-51 |  | **MDRTRGFQTSLLCGTIRRSKMGFHSKSFRFEGGGRQNKIG** | **LNRTGKSCRL** |

**R2R3-MYB Type**

Motif 1 Red boxes E-value: 6.9e-2054 Site Count: 88 Width: 29


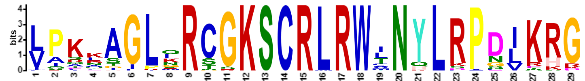


| 6. | CsMYB16 | 33 | 4.48e-38 | **QQHGHGSWRL** | **VPKKAGLQRCGKSCRLRWTNYLRPDIKRG** | **KFTLQEERTI** |
| --- | --- | --- | --- | --- | --- | --- |
| 57. | CsMYB67 | 40 | 2.54e-36 | **QLHGEGHWRI** | **VPKKAGLLRCGKSCRLRWMNYLRPDIKRG** | **NITADEDDLI** |
| 87. | CsMYB97 | 40 | 3.63e-36 | **TKHGHGCWSS** | **VPKLAGLQRCGKSCRLRWINYLRPDLKRG** | **PFSQQEENLI** |
| 86. | CsMYB96 | 40 | 3.63e-36 | **TKHGHGCWSS** | **VPKLAGLQRCGKSCRLRWINYLRPDLKRG** | **PFSQQEENLI** |
| 68. | CsMYB78 | 40 | 3.63e-36 | **STYGHGCWSS** | **VPKLAGLQRCGKSCRLRWINYLRPDLKRG** | **SFSPQEAALI** |
| 52. | CsMYB62 | 40 | 3.63e-36 | **THFGVGCWSS** | **VPKLAGLQRCGKSCRLRWINYLRPDLKRG** | **MFSQQEEDLI** |
| 70. | CsMYB80 | 40 | 4.72e-36 | **KVHGEGKWRS** | **LPKRAGLKRCGKSCRLRWLNYLRPDIKRG** | **NISPDEEELI** |
| 66. | CsMYB76 | 40 | 7.51e-36 | **KTHGEGKWRN** | **LPKQAGLQRCGKSCRLRWLNYLRPDIKRG** | **NISEEEEDLI** |
| 50. | CsMYB60 | 40 | 7.51e-36 | **HKYGHGNWRA** | **LPKLAGLLRCGKSCRLRWINYLRPDIKRG** | **NFTKEEEETI** |
| 20. | CsMYB30 | 40 | 7.51e-36 | **QKHGYGNWRT** | **LPKNAGLQRCGKSCRLRWTNYLRPDIKRG** | **RFSFEEEETI** |
| 18. | CsMYB28 | 40 | 7.51e-36 | **QKNGPGNWRN** | **LPKNAGLQRCGKSCRLRWTNYLRPDIKRG** | **RFSFEEEETI** |
| 51. | CsMYB61 | 40 | 1.18e-35 | **QKFGHANWRA** | **LPKQAGLLRCGKSCRLRWINYLRPDIKRG** | **NFTAEEEQTI** |
| 15. | CsMYB25 | 41 | 1.18e-35 | **QQFGHANWRA** | **LPKQAGLLRCGKSCRLRWINYLRPDIKRG** | **NFSKEEEDTI** |
| 74. | CsMYB84 | 40 | 1.48e-35 | **LNNGIHCWRM** | **VPKLAGLLRCGKSCRLRWINYLRPDLKRG** | **GFTELEENQI** |
| 19. | CSMYB29 | 40 | 1.48e-35 | **KRNGHGRWRT** | **LPKNAGLKRCGKSCRLRWTNYLRPDIKRG** | **RFSFEEEETI** |
| 17. | CsMYB27 | 41 | 1.48e-35 | **KNNGHGSWRS** | **LPKLAGLLRCGKSCRLRWTNYLRPDIKRG** | **PFTQDEEKLI** |
| 49. | CsMYB59 | 39 | 2.20e-35 | **NQHGEGCWRT** | **LPKAAGLLRCGKSCRLRWINYLRPDLKRG** | **NFAEDEEDLI** |
| 38. | CsMYB48 | 40 | 2.20e-35 | **RAHGEGCWRS** | **LPKAAGLLRCGKSCRLRWINYLRPDLKRG** | **NFTEEEDELI** |
| 12. | CsMYB22 | 40 | 2.20e-35 | **KLHGEGCWRS** | **LPKAAGLLRCGKSCRLRWINYLRPDLKRG** | **NFTEDEDELI** |
| 11. | CsMYB21 | 40 | 2.20e-35 | **KLHGEGCWRS** | **LPKAAGLLRCGKSCRLRWINYLRPDLKRG** | **NFTEDEDELI** |
| 16. | CsMYB26 | 40 | 2.67e-35 | **LTNGQCCWRA** | **VPKLAGLLRCGKSCRLRWTNYLRPDLKRG** | **LLSDYEEQMV** |
| 9. | CsMYB19 | 40 | 2.67e-35 | **EQHGHGSWRA** | **LPAKAGLQRCGKSCRLRWTNYLRPDIKRG** | **KFSLQEEQTI** |
| 7. | CsMYB17 | 40 | 2.67e-35 | **EEHGHGSWRA** | **LPAKAGLQRCGKSCRLRWTNYLRPDIKRG** | **KFSLQEEQTI** |
| 24. | CsMYB34 | 42 | 3.28e-35 | **QKNGHENWRA** | **LPKQAGLLRCGKSCRLRWINYLRPDVKRG** | **NFSNEEEEAI** |
| 8. | CsMYB18 | 40 | 3.93e-35 | **TRNGHGSWRA** | **LPKLAGLNRCGKSCRLRWTNYLRPDIKRG** | **KFSEDEEKLI** |
| 25. | CsMYB35 | 40 | 8.06e-35 | **LTNGHCCWRA** | **VPKLAGLRRCGKSCRLRWTNYLRPDLKRG** | **LLTEAEEQLV** |
| 47. | CsMYB57 | 39 | 1.37e-34 | **TTHGEGCWRS** | **LPKAAGLHRCGKSCRLRWINYLRPDIKRG** | **NFGEDEEDLI** |
| 5. | CsMYB15 | 40 | 6.55e-34 | **QKNGHGSWRA** | **LPRLAGLNRCGKSCRLRWTNYLRPDIKRG** | **KFSQEEEETI** |
| 14. | CsMYB24 | 40 | 9.09e-34 | **KRHGHGTWRS** | **LPKHAGLLRCGKSCRLRWTNYLRPGIKRG** | **PFSQEEESTI** |
| 23. | CsMYB33 | 40 | 1.25e-33 | **TTHGYGCWSE** | **VPEKAGLQRCGKSCRLRWINYLRPDIRRG** | **RFTPEEEKLI** |
| 22. | CsMYB32 | 40 | 1.25e-33 | **TTHGYGCWSE** | **VPEKAGLQRCGKSCRLRWINYLRPDIRRG** | **RFTPEEEKLI** |
| 83. | CsMYB93 | 52 | 1.46e-33 | **LNNGQGCWSD** | **VARNAGLQRCGKSCRLRWINYLRPDLKRG** | **AFSPQEEELI** |
| 39. | CsMYB49 | 40 | 1.71e-33 | **QQHGEGGWRS** | **LPQKAGLSRCGKSCRLRWANYLRPDIKRG** | **AFSPEEELTI** |
| 28. | CsMYB38 | 40 | 2.00e-33 | **TRYGCWNWRQ** | **LPKFAGLKRCGKSCRLRWMNYLRPNVKRG** | **NYTHEEEETI** |
| 64. | CsMYB74 | 65 | 3.14e-33 | **AQHGTRNWRL** | **IPKNAGLQRCGKSCRLRWTNYLRPDLKHG** | **QFSDAEEQTI** |
| 42. | CsMYB52 | 40 | 3.64e-33 | **HKNGPGKWRD** | **LPKRAGLKRCGKSCRLRWLNYLRPDITRG** | **NISTDEDDLI** |
| 41. | CsMYB51 | 40 | 3.64e-33 | **HKNGPGKWRD** | **LPKRAGLKRCGKSCRLRWLNYLRPDITRG** | **NISTDEDDLI** |
| 21. | CsMYB31 | 41 | 3.64e-33 | **KYGTGGNWIA** | **LPQKAGLKRCGKSCRLRWLNYLRPNIKHG** | **EFSDEEDRII** |
| 46. | CsMYB56 | 52 | 4.22e-33 | **RKEGEGRWRT** | **LPKRAGLLRCGKSCRLRWMNYLRPSVKRG** | **HIQPDEEDLI** |
| 69. | CsMYB79 | 39 | 6.53e-33 | **TTHGHGNWSS** | **VPKLAGLQRCGKSCRLRWINYLRPDLRKG** | **SFTQEEEQII** |
| 43. | CsMYB53 | 40 | 8.69e-33 | **QTHGEGSWRS** | **LPKNAGLLRCGKSCRLRWINYLRADLKRG** | **NITSEEEETI** |
| 27. | CsMYB37 | 40 | 8.69e-33 | **QSNGEGSWRS** | **LPKNAGLLRCGKSCRLRWINYLRADLKRG** | **NISSEEEDII** |
| 37. | CsMYB47 | 41 | 1.00e-32 | **NNGTGGNWIA** | **LPHKAGLNRCGKSCRLRWLNYLRPDIKHG** | **GFTEEEDNVI** |
| 73. | CsMYB83 | 40 | 1.33e-32 | **SKLGIGNWTL** | **VPKKAGLNRCGKSCRLRWTNYLRPDLKHD** | **TFTPQEEDLI** |
| 13. | CsMYB23 | 43 | 1.53e-32 | **RNGHHGSWKS** | **LPKLAGLIRCGKSCRLRWNNYLRPDIKRG** | **KFSEDEERII** |
| 65. | CsMYB75 | 40 | 1.76e-32 | **KQHGEGGWRH** | **LPQKAGLSRCGKSCRLRWANYLRPGIKRG** | **EFSPEEEQTI** |
| 56. | CsMYB66 | 40 | 2.02e-32 | **TQHGEGGWRS** | **LPEKAGLSRCGKSCRLRWTNYLRPGIKRG** | **EFTNEEEEII** |
| 63. | CsMYB73 | 68 | 3.06e-32 | **LRNGQGCWSD** | **IARNAGLQRCGKSCRLRWINYLRPDLKRG** | **AFSSHEQDLI** |
| 55. | CsMYB65 | 40 | 4.59e-32 | **ERYGHWNWRE** | **LPKFAGLSRCGKSCRLRWMNYLRPDVRRG** | **NYTAEEENTI** |
| 32. | CsMYB42 | 41 | 1.91e-31 | **NHGTGGNWIA** | **LPQKIGLKRCGKSCRLRWLNYLRPNIKHG** | **GFSEEEDNII** |
| 31. | CsMYB41 | 41 | 1.91e-31 | **TYGTGGNWIA** | **LPQKIGLKRCGKSCRLRWLNYLRPNIKHG** | **GFSEEEDNII** |
| 82. | CsMYB92 | 44 | 2.78e-31 | **SLHGEGHWNL** | **LAKRAGLKRTGKSCRLRWLNYLKPDIKRG** | **NLTPQEQLLI** |
| 81. | CsMYB91 | 44 | 2.78e-31 | **SLHGEGHWNL** | **LAKRAGLKRTGKSCRLRWLNYLKPDIKRG** | **NLTPQEQLLI** |
| 76. | CsMYB86 | 46 | 3.14e-31 | **ANHGEGVWNS** | **LAKAAGLKRTGKSCRLRWLNYLRPDVRRG** | **NITNEEQLII** |
| 34. | CsMYB44 | 41 | 9.38e-31 | **LKHGHGCWSS** | **VPIKAGLQRNGKSCRLRWINYLRPGLKRG** | **TFSQHEEETI** |
| 33. | CsMYB43 | 41 | 1.34e-30 | **QNGTGGNWIA** | **LPQKIGLKRCGKSCRLRWLNYLRPHIKHG** | **GFSEEEDNII** |
| 36. | CsMYB46 | 48 | 1.51e-30 | **RLHGEGRWNS** | **VARLAGLKRNGKSCRLRWVNYLRPDLKRG** | **QITPHEESII** |
| 72. | CsMYB82 | 55 | 4.74e-30 | **DKYGEGKWHL** | **VPLRAGLNRCRKSCRLRWLNYLKPDIKRG** | **EFTADEVDLI** |
| 54. | CsMYB64 | 59 | 4.74e-30 | **SHHGAQKWKT** | **VAAIAGLNRCGKSCRMRWLNYLRPNIKRG** | **NITDQEEDLI** |
| 84. | CsMYB94 | 81 | 8.28e-30 | **AIHGEGRWNS** | **LARCAGLKRTGKSCRLRWLNYLRPDVRRG** | **NITLEEQLLI** |
| 77. | CsMYB87 | 41 | 1.03e-29 | **DKYGEGKWHL** | **VPLRAGLSRCRKSCRLRWLNYLKPDIKRG** | **EFEEDEVDLV** |
| 4. | CsMYB14 | 40 | 1.29e-29 | **QEHGPGNWRA** | **VPTNTGLLRCSKSCRLRWTNYLRPGIKRG** | **NFTDNEEKMI** |
| 3. | CsMYB13 | 40 | 1.29e-29 | **QEHGPGNWRA** | **VPTNTGLLRCSKSCRLRWTNYLRPGIKRG** | **NFTDNEEKMI** |
| 26. | CsMYB36 | 41 | 1.78e-29 | **KKYGIWNWTE** | **MSKAAGLNRSGKSCRLRWVNYLRPDIKHG** | **NFSQEEKEII** |
| 53. | CsMYB63 | 51 | 2.21e-29 | **TRHGEGRWNL** | **LAKRSGLRRTGKSCRLRWLNYLKPDVKRG** | **NLTPEEQILI** |
| 1. | CsMYB11 | 40 | 2.73e-29 | **QEHGPGNWRS** | **VPTNTGLMRCSKSCRLRWTNYLRPGIKRG** | **NFTPHEEGMI** |
| 29. | CsMYB39 | 41 | 3.76e-29 | **KLHGPRRWKT** | **LSIQSGLNRCGKSCRLRWLNYLRPNIKRG** | **NISEAEEDLI** |
| 10. | CsMYB20 | 46 | 4.17e-29 | **SFNGEGRWSS** | **VARSAGLNRSGKSCRLRWVNYLRPGLKRG** | **QITPQEEGII** |
| 2. | CsMYB12 | 40 | 4.17e-29 | **QEHGPGNWRS** | **VPTHTGLMRCSKSCRLRWTNYLRPGIKRG** | **NFTDHEEKMI** |
| 67. | CsMYB77 | 65 | 1.06e-28 | **SIHGEGRWNS** | **LARHAGLKRTGKSCRLRWLNYLRPSVRRG** | **NITLQEQLLI** |
| 62. | CsMYB72 | 60 | 2.90e-28 | **KKHGEGNWNS** | **VQKYSGLNRCGKSCRLRWANHLRPNLKKG** | **SFTPEEERLI** |
| 60. | CsMYB70 | 15 | 2.90e-28 | **KKHGEGNWNS** | **VQKYSGLNRCGKSCRLRWANHLRPNLKKG** | **SFTPEEERLI** |
| 58. | CsMYB68 | 60 | 2.90e-28 | **KKHGEGNWNS** | **VQKYSGLNRCGKSCRLRWANHLRPNLKKG** | **SFTPEEERLI** |
| 45. | CsMYB55 | 48 | 5.24e-28 | **QLMGEKRWDA** | **LAKASGLRRSGKSCRLRWLNYLRPNLKHD** | **QISAEEENII** |
| 44. | CsMYB54 | 36 | 5.24e-28 | **QLMGEKRWDA** | **LAKASGLRRSGKSCRLRWLNYLRPNLKHD** | **QISAEEENII** |
| 35. | CsMYB45 | 40 | 7.02e-28 | **ETHGEGNWQT** | **VSKLTGLKRGGKSCRLRWKNYLRPNIKRG** | **GMSQEEEDLI** |
| 40. | CsMYB50 | 65 | 8.51e-28 | **RKHGEGNWNA** | **VQKNSGLARCGKSCRLRWANHLRPNLKKG** | **SFSPDEERII** |
| 71. | CsMYB81 | 40 | 1.15e-26 | **SKHGKAKWTS** | **VPKGAGLRRCGKSCRLRWNNHLRPDLNHE** | **SFTPKEEELI** |
| 61. | CsMYB71 | 86 | 6.89e-24 | **VSKFGARNWS** | **LIARGIPGRSGKSCRLRWCNQLDPCVKRK** | **PFTEEEDRLI** |
| 59. | CsMYB69 | 82 | 6.89e-24 | **VSKFGARNWS** | **LIARGIPGRSGKSCRLRWCNQLDPCVKRK** | **PFTEEEDRLI** |
| 48. | CsMYB58 | 37 | 1.18e-23 | **NKYGPRDWSS** | **IRSKGLLQRTGKSCRLRWVNKLRPNLKNG** | **CKFSLEEERV** |
| 30. | CsMYB40 | 38 | 2.01e-23 | **KKNGPRDWSS** | **IRSKGLLPRTGKSCRLRWVNKLRPDLKTG** | **CKFSAEEERL** |
| 75. | CsMYB85 | 35 | 2.71e-23 | **VKQHSARNWS** | **VISKSIPGRSGKSCRLRWCNQLSPEVKHR** | **PFTDMEDQII** |
| 80. | CsMYB90 | 38 | 2.44e-22 | **VEKHGPRNWS** | **LISKSIPGRSGKSCRLRWCNQLSPQVEHR** | **AFTAEEDDTI** |
| 79. | CsMYB89 | 38 | 2.44e-22 | **VEKHGPRNWS** | **LISKSIPGRSGKSCRLRWCNQLSPQVEHR** | **AFTAEEDDTI** |
| 85. | CsMYB95 | 66 | 1.63e-21 | **VDRYGARNWS** | **LISRYIKGRSGKSCRLRWCNQLSPSVEHR** | **PFSPAEDETI** |
| 78. | CsMYB88 | 33 | 2.64e-21 | **VEQHGPRNWS** | **VISTGIIGRSGKSCRLRWCNQLCPSVQHR** | **PFTPEEDSMI** |
| 88. | CsMYB98 | 48 | 1.28e-19 | **VDRYGPQNWN** | **FIAEHLQGRSGKSCRLRWYNQLDPNINKK** | **PFTEEEEERL** |

Motif 2 light blue boxes E-value: 1.3e-2992 Site Count: 88 Width: 50


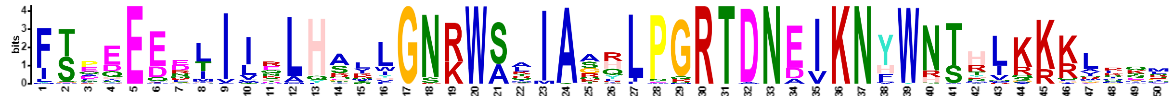


| 19. | CSMYB29 | 70 | 5.53e-52 | **YLRPDIKRGR** | **FSFEEEETIIQLHSVLGNKWSAIAARLPGRTDNEIKNYWNTHIRKRLLKM** | **GIDPVTHTPR** |
| --- | --- | --- | --- | --- | --- | --- |
| 18. | CsMYB28 | 70 | 1.40e-50 | **YLRPDIKRGR** | **FSFEEEETIIQLHSVLGNKWSAIAARLPGRTDNEIKNYWNTHIRKRLLRM** | **GIDPVTHAPR** |
| 5. | CsMYB15 | 70 | 2.48e-50 | **YLRPDIKRGK** | **FSQEEEETILNLHSILGNKWSAIASHLPGRTDNEIKNFWNTHLKKKLIQM** | **GIDPMTHRPR** |
| 20. | CsMYB30 | 70 | 2.99e-50 | **YLRPDIKRGR** | **FSFEEEETIIQLHSILGNKWSAIAARLPGRTDNEIKNYWNTHIRKRLLRM** | **GIDPVTHSPR** |
| 8. | CsMYB18 | 70 | 6.27e-50 | **YLRPDIKRGK** | **FSEDEEKLIINLHAVLGNKWSTIAGHLPGRTDNEIKNLWNTHLKKKLLQM** | **GIDPVTHRPR** |
| 7. | CsMYB17 | 70 | 4.39e-49 | **YLRPDIKRGK** | **FSLQEEQTIIQLHALLGNRWSAIATHLPKRTDNEIKNYWNTHLKKRLTKM** | **GIDPVTHKPK** |
| 49. | CsMYB59 | 69 | 7.30e-49 | **YLRPDLKRGN** | **FAEDEEDLIIKLHALLGNRWSLIAGRLPGRTDNEIKNYWNSHLRRKLINM** | **GIDPNNHRLK** |
| 38. | CsMYB48 | 70 | 1.21e-48 | **YLRPDLKRGN** | **FTEEEDELIIKLHSLLGNKWSLIAGRLPGRTDNEIKNYWNTHIRRKLLNR** | **GIDPATHRPL** |
| 6. | CsMYB16 | 63 | 1.42e-48 | **YLRPDIKRGK** | **FTLQEERTIIQLHALLGNRWSAIASHLPNRTDNEIKNYWNTHLKKRLTTM** | **GIDPVTHKPL** |
| 17. | CsMYB27 | 71 | 1.68e-48 | **YLRPDIKRGP** | **FTQDEEKLIIQLHGMLGNRWAAIASQLPGRTDNEIKNLWNTHLKKRLLCM** | **GLDPLTHEPF** |
| 87. | CsMYB97 | 70 | 3.21e-48 | **YLRPDLKRGP** | **FSQQEENLIIELHAVLGNRWSQIAAQLPGRTDNEIKNLWNSCIKKKLRQK** | **GIDPNTHKPL** |
| 86. | CsMYB96 | 70 | 3.21e-48 | **YLRPDLKRGP** | **FSQQEENLIIELHAVLGNRWSQIAAQLPGRTDNEIKNLWNSCIKKKLRQK** | **GIDPNTHKPL** |
| 66. | CsMYB76 | 70 | 3.21e-48 | **YLRPDIKRGN** | **ISEEEEDLIIRLHKLLGNRWSLIAGRLPGRTDNEIKNYWNTTLAKKLVQK** | **QQQIEENDTK** |
| 12. | CsMYB22 | 70 | 3.21e-48 | **YLRPDLKRGN** | **FTEDEDELIINLHSLLGNKWSLIASRLPGRTDNEIKNYWNTHIKRKLYSR** | **GIDPQTHRPL** |
| 11. | CsMYB21 | 70 | 3.21e-48 | **YLRPDLKRGN** | **FTEDEDELIINLHSLLGNKWSLIASRLPGRTDNEIKNYWNTHIKRKLYSR** | **GIDPQTHRPL** |
| 13. | CsMYB23 | 73 | 3.77e-48 | **YLRPDIKRGK** | **FSEDEERIIINLHSALGNKWSRIASHLPGRTDNEIKNFWNTYLRKKLLQM** | **GIDPQTHKPR** |
| 51. | CsMYB61 | 70 | 8.31e-48 | **YLRPDIKRGN** | **FTAEEEQTIINLHQMLGNRWSAIAARLPGRTDNEIKNVWHTHLKKRLLNN** | **NNINSNNLSS** |
| 50. | CsMYB60 | 70 | 1.13e-47 | **YLRPDIKRGN** | **FTKEEEETIIELHQMLGNRWSAIAARLPGRTDNEIKNVWHTHLKKRLIIN** | **NNDDVIVQKS** |
| 52. | CsMYB62 | 70 | 1.32e-47 | **YLRPDLKRGM** | **FSQQEEDLIISLHQVLGNRWAQIAAQLPGRTDNEIKNFWNSCLKKKLVKQ** | **GIDPTTHKPL** |
| 47. | CsMYB57 | 69 | 1.32e-47 | **YLRPDIKRGN** | **FGEDEEDLIIKLHALLGNRWSLIAGRLPGRTDNEVKNYWNSHLRKKLINL** | **GIDPNNHKVN** |
| 83. | CsMYB93 | 82 | 2.09e-47 | **YLRPDLKRGA** | **FSPQEEELIIHLHSLLGNRWSQIAARLPGRTDNEIKNFWNSTVKKRLKNL** | **SSSSTTSPNT** |
| 62. | CsMYB72 | 90 | 2.09e-47 | **HLRPNLKKGS** | **FTPEEERLILELHAKYGNKWARMAAQLPGRTDNEIKNYWNTRVKRRKRQG** | **LPLYPHDIQH** |
| 60. | CsMYB70 | 45 | 2.09e-47 | **HLRPNLKKGS** | **FTPEEERLILELHAKYGNKWARMAAQLPGRTDNEIKNYWNTRVKRRKRQG** | **LPLYPHDIQH** |
| 58. | CsMYB68 | 90 | 2.09e-47 | **HLRPNLKKGS** | **FTPEEERLILELHAKYGNKWARMAAQLPGRTDNEIKNYWNTRVKRRKRQG** | **LPLYPHDIQH** |
| 9. | CsMYB19 | 70 | 2.09e-47 | **YLRPDIKRGK** | **FSLQEEQTIIQLHALLGNRWSAIATHLAKRTDNEIKNYWNTHLKKRLAKM** | **GIDPITHKPK** |
| 2. | CsMYB12 | 70 | 2.09e-47 | **YLRPGIKRGN** | **FTDHEEKMIIHLQALLGNRWAAIASYLPQRTDNDIKNYWNTHLKKKLRKL** | **HTGHDGQSHD** |
| 25. | CsMYB35 | 70 | 5.94e-47 | **YLRPDLKRGL** | **LTEAEEQLVIDLHARLGNRWSKIASRLPGRTDNEIKNHWNTHIKKKLLKM** | **GIDPVTHEPL** |
| 54. | CsMYB64 | 89 | 5.01e-46 | **YLRPNIKRGN** | **ITDQEEDLILRLHKLLGNRWSLIAGRLPGRTDNEIKNYWNSHLCKKMSQK** | **EKKKKKIQME** |
| 14. | CsMYB24 | 70 | 5.75e-46 | **YLRPGIKRGP** | **FSQEEESTIIQLHAMFGNRWAVIASQIPGRTDNEIKNYWNTHLKKRGICL** | **AENLQHSLLL** |
| 57. | CsMYB67 | 70 | 7.56e-46 | **YLRPDIKRGN** | **ITADEDDLIIRLHSLLGNRWSLIAGRLPGRTDNEIKNYWNSHLSKRLNTN** | **NKERTKKKQQ** |
| 73. | CsMYB83 | 70 | 1.30e-45 | **YLRPDLKHDT** | **FTPQEEDLIINLHQAIGSRWSLIAKQLPGRTDNDVKNYWNTKLRKKLYTM** | **GIDPVTHKPI** |
| 23. | CsMYB33 | 70 | 1.48e-45 | **YLRPDIRRGR** | **FTPEEEKLIISLHGVVGNRWAHIASHLPGRTDNEIKNYWNSWIKKKIRKS** | **SSTNSINSNT** |
| 22. | CsMYB32 | 70 | 1.48e-45 | **YLRPDIRRGR** | **FTPEEEKLIISLHGVVGNRWAHIASHLPGRTDNEIKNYWNSWIKKKIRKS** | **SSTNSINSNT** |
| 40. | CsMYB50 | 95 | 1.94e-45 | **HLRPNLKKGS** | **FSPDEERIIIELHAKIGNKWARMASQLPGRTDNEIKNYWNTRMKRRQRAG** | **LPLYPHDIRH** |
| 16. | CsMYB26 | 70 | 2.52e-45 | **YLRPDLKRGL** | **LSDYEEQMVIDLHAQLGNRWSKIASHLPGRTDNEIKNHWNTHIKKKLKKM** | **GIDPLTHQPL** |
| 45. | CsMYB55 | 78 | 3.73e-45 | **YLRPNLKHDQ** | **ISAEEENIIIQLHERWGNKWSKIARILPGRTDNEIKNYWRTYLRKKLAQN** | **QEEKGKEDIA** |
| 44. | CsMYB54 | 66 | 3.73e-45 | **YLRPNLKHDQ** | **ISAEEENIIIQLHERWGNKWSKIARILPGRTDNEIKNYWRTYLRKKLAQN** | **QEEKGKEDIA** |
| 4. | CsMYB14 | 70 | 5.50e-45 | **YLRPGIKRGN** | **FTDNEEKMIIHLQALLGNRWAAIASYLPQRTDNDIKNYWNTYLKKKLSKI** | **QNQSSSRDDH** |
| 3. | CsMYB13 | 70 | 5.50e-45 | **YLRPGIKRGN** | **FTDNEEKMIIHLQALLGNRWAAIASYLPQRTDNDIKNYWNTYLKKKLSKI** | **QNQSSSRDDH** |
| 29. | CsMYB39 | 71 | 6.26e-45 | **YLRPNIKRGN** | **ISEAEEDLILRLHKLLGNRWALIAGRLPGRTDNEIKNYWNSHLSKKINQS** | **QHQPSLSQQQ** |
| 1. | CsMYB11 | 70 | 6.26e-45 | **YLRPGIKRGN** | **FTPHEEGMIIHLQALLGNKWASIASYLPQRTDNDIKNYWNTHLKKKLKKF** | **HSALEPNNDI** |
| 34. | CsMYB44 | 71 | 1.04e-44 | **YLRPGLKRGT** | **FSQHEEETILTLHHMLGNKWSQIAQHLPGRTDNEIKNYWHSYLKKKVAKA** | **EEELLTEQST** |
| 64. | CsMYB74 | 95 | 1.52e-44 | **YLRPDLKHGQ** | **FSDAEEQTIVKLHSIVGNRWSIIAAQLPGRTDNDVKNHWNTKLKKKLSGM** | **GIDPVTHKPF** |
| 10. | CsMYB20 | 76 | 1.52e-44 | **YLRPGLKRGQ** | **ITPQEEGIIIELHALWGNKWSTIARYLPGRTDNEIKNFWRTHFKKKDKAK** | **YSRKQQKRRS** |
| 15. | CsMYB25 | 71 | 1.72e-44 | **YLRPDIKRGN** | **FSKEEEDTIIKLHHNLGNRWSSIATQLPGRTDNEIKNFWHTHLKKKIKKK** | **LIMKQSSNSE** |
| 71. | CsMYB81 | 70 | 1.95e-44 | **HLRPDLNHES** | **FTPKEEELIVKLHAAIGSRWSMIAQQLPGRTDNDVKNYWNTKLRKKLSEM** | **GIDPVTHKPF** |
| 63. | CsMYB73 | 98 | 2.21e-44 | **YLRPDLKRGA** | **FSSHEQDLILHLHSILGNRWSQIAARLPGRTDNEIKNFWNSTLKKKLNKL** | **GVNNNNNNTT** |
| 69. | CsMYB79 | 69 | 3.61e-44 | **YLRPDLRKGS** | **FTQEEEQIIIDVHRILGNRWAQIAKHLPGRTDNEVKNFWNSCIKKKLMSQ** | **GLDPQTHNLM** |
| 74. | CsMYB84 | 70 | 4.08e-44 | **YLRPDLKRGG** | **FTELEENQIIQLHSRLGNRWSKIAAHFPGRTDNEIKNHWNTRIKKRLKVL** | **GVDPITHQKI** |
| 46. | CsMYB56 | 82 | 4.08e-44 | **YLRPSVKRGH** | **IQPDEEDLILRLHRLLGNRWSLIAGRIPGRTDNEIKNYWNTHLSKKLINQ** | **GIDPRTHKPF** |
| 70. | CsMYB80 | 70 | 1.35e-43 | **YLRPDIKRGN** | **ISPDEEELIIRLHNLLGNRWSLIAGRLPGRTDNEIKNYWNTNIGKKVQVA** | **ADHHNNFLTK** |
| 56. | CsMYB66 | 70 | 1.93e-43 | **YLRPGIKRGE** | **FTNEEEEIIMRLHAVLGNKWSAIAKQLPMRTDNEIKNHWNTRLKRIVAEK** | **GKDNLITRAD** |
| 42. | CsMYB52 | 70 | 2.74e-43 | **YLRPDITRGN** | **ISTDEDDLIVRLHRLLGNRWALIAGRLPGRTDNEIKNYWNTNLRKRVNNI** | **NNINININVK** |
| 41. | CsMYB51 | 70 | 2.74e-43 | **YLRPDITRGN** | **ISTDEDDLIVRLHRLLGNRWALIAGRLPGRTDNEIKNYWNTNLRKRVNNI** | **NNINININVK** |
| 39. | CsMYB49 | 70 | 3.07e-43 | **YLRPDIKRGA** | **FSPEEELTIVRLHSVLGNRWSAIAKNLPKRTDNEIKNHWNTRLKKCLIES** | **AAYHHQNYNS** |
| 36. | CsMYB46 | 78 | 3.45e-43 | **YLRPDLKRGQ** | **ITPHEESIILELHARWGNRWSTIARSLPGRTDNEIKNYWRTHFKKKQAKV** | **TSDASEKAKA** |
| 82. | CsMYB92 | 74 | 4.87e-43 | **YLKPDIKRGN** | **LTPQEQLLILELHSKWGNRWSRIAQHLPGRTDNEIKNYWRTRVQKQARQL** | **NIESNSKKFI** |
| 81. | CsMYB91 | 74 | 4.87e-43 | **YLKPDIKRGN** | **LTPQEQLLILELHSKWGNRWSRIAQHLPGRTDNEIKNYWRTRVQKQARQL** | **NIESNSSLLL** |
| 26. | CsMYB36 | 71 | 4.87e-43 | **YLRPDIKHGN** | **FSQEEKEIIVTLHETLGNRWSAIAAKLPGRTDNEVKNYWHTHLKKRFQKQ** | **LPLHSSSMSI** |
| 84. | CsMYB94 | 111 | 6.12e-43 | **YLRPDVRRGN** | **ITLEEQLLILELHSRWGNRWSKIAQHLPGRTDNEIKNYWRTRVQKHAKQL** | **KCDVNSKQFK** |
| 27. | CsMYB37 | 70 | 6.86e-43 | **YLRADLKRGN** | **ISSEEEDIIINLHSTLGNRWSLIASHLPGRTDNEIKNYWNSHLSRKIHTF** | **RRCNTTHHIH** |
| 24. | CsMYB34 | 72 | 8.60e-43 | **YLRPDVKRGN** | **FSNEEEEAIIKLHETLGNKWSKIASHFPGRTDNEIKNVWNTHLKKRLSSS** | **SKDGDDQPNP** |
| 67. | CsMYB77 | 95 | 9.62e-43 | **YLRPSVRRGN** | **ITLQEQLLILELHSRWGNRWSKIAQYLPGRTDNEIKNYWRTRVQKQAKQL** | **KCDVNSKQFQ** |
| 35. | CsMYB45 | 70 | 1.35e-42 | **YLRPNIKRGG** | **MSQEEEDLIIRMHKLLGNRWSLIAGRLPGRTDNEVKNYWNTHLNKKTSAV** | **GKRKRVNNNS** |
| 76. | CsMYB86 | 76 | 1.88e-42 | **YLRPDVRRGN** | **ITNEEQLIIMELHAKWGNRWSKIAKHLPGRTDNEIKNYWRTRIQKHMKQA** | **DHHPNQNNNN** |
| 21. | CsMYB31 | 71 | 2.93e-42 | **YLRPNIKHGE** | **FSDEEDRIICNLFANIGSRWSIIAAHLPGRTDNDIKNYWNTKLKKKLMGL** | **APQISQRKVP** |
| 61. | CsMYB71 | 116 | 3.64e-42 | **QLDPCVKRKP** | **FTEEEDRLIVTAHAIHGNKWAAIARLLPGRTDNAIKNHWNSTLKRKYPEC** | **GRFNTGPEEM** |
| 59. | CsMYB69 | 112 | 3.64e-42 | **QLDPCVKRKP** | **FTEEEDRLIVTAHAIHGNKWAAIARLLPGRTDNAIKNHWNSTLKRKYPEC** | **GRFNTGPEEM** |
| 80. | CsMYB90 | 68 | 4.53e-42 | **QLSPQVEHRA** | **FTAEEDDTIIRAHARFGNKWATIARLLNGRTDNAIKNHWNSTLKRKCSTM** | **LEETDNGHGH** |
| 79. | CsMYB89 | 68 | 4.53e-42 | **QLSPQVEHRA** | **FTAEEDDTIIRAHARFGNKWATIARLLNGRTDNAIKNHWNSTLKRKCSTM** | **LEETDNGHGH** |
| 31. | CsMYB41 | 71 | 6.26e-42 | **YLRPNIKHGG** | **FSEEEDNIICSLYISIGSRWSIIAAQLPGRTDNDIKNYWNTRLKKKLLGR** | **RKQSNGSRLS** |
| 28. | CsMYB38 | 70 | 6.26e-42 | **YLRPNVKRGN** | **YTHEEEETITRLHASLGNKWSAIAAELPGRTDNEVKNYWHTNLKKRQKQN** | **SSSVEISQLE** |
| 32. | CsMYB42 | 71 | 8.65e-42 | **YLRPNIKHGG** | **FSEEEDNIICSLYISIGSRWSIIAAQLPGRTDNDIKNYWNTRLKKKLLGN** | **KQRKDHPRNK** |
| 68. | CsMYB78 | 70 | 9.62e-42 | **YLRPDLKRGS** | **FSPQEAALIIELHTILGNRWAQIAKHLPGRTDNEVKNFWNSSIKKKLLSG** | **HDNHLHHHHH** |
| 43. | CsMYB53 | 70 | 1.32e-41 | **YLRADLKRGN** | **ITSEEEETIVNLHNALGNRWSVIADHLPGRTDNEIKNYWNSHLSRKIYSF** | **TKRLPNESSS** |
| 33. | CsMYB43 | 71 | 1.32e-41 | **YLRPHIKHGG** | **FSEEEDNIICSLYLSIGSRWSIIAAQLPGRTDNDIKNYWNTRLKKKLLGK** | **QRKEHAAAAA** |
| 72. | CsMYB82 | 85 | 2.02e-41 | **YLKPDIKRGE** | **FTADEVDLILRLHKLLGNRWSLIAGRIPGRTANDVKNYWNTHLGKKVMMS** | **YDKLNQKHYL** |
| 55. | CsMYB65 | 70 | 2.02e-41 | **YLRPDVRRGN** | **YTAEEENTIIKLHQQHGKKWSMIAAKLPGRTDNEIKNHWHTHLKKRAITN** | **NYNNNNNSDH** |
| 85. | CsMYB95 | 96 | 4.19e-41 | **QLSPSVEHRP** | **FSPAEDETILAAHAQYGNRWATIARLLPGRTDNAVKNHWNSTLKRRAREA** | **HQMEDEGNQN** |
| 53. | CsMYB63 | 81 | 4.19e-41 | **YLKPDVKRGN** | **LTPEEQILILDLHSKWGNRWSKIAQYLPGRTDNEIKNYWRTRVQKQAKHL** | **KIDAESTAFQ** |
| 65. | CsMYB75 | 70 | 7.01e-41 | **YLRPGIKRGE** | **FSPEEEQTIIRLHAILGNKWSIISRHLYRRTDNEVKNYWNTRLKKRGTTE** | **INSKDSPVRH** |
| 37. | CsMYB47 | 71 | 1.43e-40 | **YLRPDIKHGG** | **FTEEEDNVIWTLYSNIGSRWSVIASQLPGRTDNDVKNYWNTKLKKKLLAR** | **STNSNETTDN** |
| 78. | CsMYB88 | 63 | 1.03e-39 | **QLCPSVQHRP** | **FTPEEDSMIIQAHAAHGNKWATIARLLPGRTDNAIKNHWNSTLRRGRRAG** | **DKFSSSSFTS** |
| 77. | CsMYB87 | 71 | 4.74e-39 | **YLKPDIKRGE** | **FEEDEVDLVLRLHKLLGNRWSLIAGRIPGRTANDVKNYWNTHQRKKMVII** | **NIDQSNKLNN** |
| 75. | CsMYB85 | 65 | 1.74e-38 | **QLSPEVKHRP** | **FTDMEDQIIIDAHSKHGNKWATIARLLEGRTDNAIKNHWNSTLKRKCSSS** | **ENDDVDDDET** |
| 30. | CsMYB40 | 69 | 4.33e-38 | **LRPDLKTGCK** | **FSAEEERLVIELQAQVGNKWAKIATYLPGRTDNDVKNFWSSRKKKLQRLN** | **HHRRPSSVPL** |
| 48. | CsMYB58 | 68 | 6.79e-38 | **LRPNLKNGCK** | **FSLEEERVVIELQAQFGNKWARIATYLPGRTDNDVKNFWSSRQKRLARIL** | **QTPPTSSKSQ** |
| 88. | CsMYB98 | 78 | 1.87e-32 | **QLDPNINKKP** | **FTEEEEERLLAAHRIYGNKWACIAKYFHGRTDNAVKNHYHVVMARRKRER** | **FSSSSSSPNS** |

Motif 3 green boxes E-value: 5.6e-1162 Site Count: 87 Width: 25


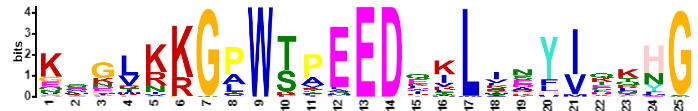


| 20. | CsMYB30 | 9 | 3.06e-29 | **MGRAPCCD** | **KNGLKKGPWTPEEDQKLVDYIQKHG** | **YGNWRTLPKN** |
| --- | --- | --- | --- | --- | --- | --- |
| 51. | CsMYB61 | 9 | 1.07e-27 | **MGRAPCCE** | **KMGLKKGPWTPEEDQILINYVQKFG** | **HANWRALPKQ** |
| 9. | CsMYB19 | 9 | 1.07e-27 | **MGRSPCCD** | **KVGLKKGPWTPEEDQKLLAYIEQHG** | **HGSWRALPAK** |
| 50. | CsMYB60 | 9 | 2.32e-27 | **MGRAPCCE** | **KMGLKKGPWTPEEDQILISYIHKYG** | **HGNWRALPKL** |
| 7. | CsMYB17 | 9 | 7.87e-27 | **MGRSPCCE** | **KVGLKKGPWTPEEDQKLLAYIEEHG** | **HGSWRALPAK** |
| 14. | CsMYB24 | 9 | 9.96e-27 | **MGKIACCD** | **KNGVKKGAWTPEEDQILVNYIKRHG** | **HGTWRSLPKH** |
| 2. | CsMYB12 | 9 | 2.48e-26 | **MGRPPCCD** | **KVGVKKGPWTPEEDIILVSYIQEHG** | **PGNWRSVPTH** |
| 4. | CsMYB14 | 9 | 3.84e-26 | **MGRPPCCD** | **KEGVKKGPWTPEEDIILVSYIQEHG** | **PGNWRAVPTN** |
| 3. | CsMYB13 | 9 | 3.84e-26 | **MGRPPCCD** | **KEGVKKGPWTPEEDIILVSYIQEHG** | **PGNWRAVPTN** |
| 39. | CsMYB49 | 9 | 5.35e-25 | **MGRSPCCN** | **VQGLKKGAWTPEEDQKLLSYIQQHG** | **EGGWRSLPQK** |
| 1. | CsMYB11 | 9 | 7.80e-25 | **MGRPPCCD** | **KVGIKKGPWTPEEDIILVSYIQEHG** | **PGNWRSVPTN** |
| 8. | CsMYB18 | 9 | 2.31e-24 | **MGRSPSCD** | **ENGLKKGPWTPEEDEKLVDYITRNG** | **HGSWRALPKL** |
| 21. | CsMYB31 | 9 | 3.28e-24 | **MGRSPCCD** | **KANVKKGPWSPEEDLKLKEYIEKYG** | **TGGNWIALPQ** |
| 87. | CsMYB97 | 9 | 4.63e-24 | **MGRHSCCY** | **KQKLRKGLWSPEEDEKLLNYITKHG** | **HGCWSSVPKL** |
| 86. | CsMYB96 | 9 | 4.63e-24 | **MGRHSCCY** | **KQKLRKGLWSPEEDEKLLNYITKHG** | **HGCWSSVPKL** |
| 13. | CsMYB23 | 11 | 1.26e-23 | **MGRSPCCDGI** | **NIGLKKGPWTPEEDKKLIDYIERNG** | **HHGSWKSLPK** |
| 15. | CsMYB25 | 10 | 1.48e-23 | **MGSRRACCE** | **NMGLRKGPWTPEEDHILISYIQQFG** | **HANWRALPKQ** |
| 5. | CsMYB15 | 9 | 1.48e-23 | **MGRSPCCD** | **ESGLKKGPWTPEEDQKLVKFIQKNG** | **HGSWRALPRL** |
| 68. | CsMYB78 | 9 | 1.74e-23 | **MGHHSCCN** | **KQKVKRGLWSPEEDEKLINYISTYG** | **HGCWSSVPKL** |
| 18. | CsMYB28 | 9 | 2.04e-23 | **MGRAPCCD** | **KNGLKKGPWTPEEDLNLINYIQKNG** | **PGNWRNLPKN** |
| 65. | CsMYB75 | 9 | 3.82e-23 | **MGRTPCCD** | **QKGLRKGAWTAEEDQILFSYIKQHG** | **EGGWRHLPQK** |
| 16. | CsMYB26 | 9 | 4.45e-23 | **MGRQPCCD** | **KVGLKKGPWTAEEDKKLINFILTNG** | **QCCWRAVPKL** |
| 31. | CsMYB41 | 9 | 5.19e-23 | **MGRAPCCD** | **KANVKKGPWSPEEDTKLKAYIDTYG** | **TGGNWIALPQ** |
| 23. | CsMYB33 | 9 | 7.02e-23 | **MGHHSCCN** | **QQKVKRGLWSPEEDEKLIRYITTHG** | **YGCWSEVPEK** |
| 22. | CsMYB32 | 9 | 7.02e-23 | **MGHHSCCN** | **QQKVKRGLWSPEEDEKLIRYITTHG** | **YGCWSEVPEK** |
| 19. | CSMYB29 | 9 | 9.46e-23 | **MGKITCCE** | **KNGLKKGPWTPEEDQALIDHIKRNG** | **HGRWRTLPKN** |
| 43. | CsMYB53 | 9 | 1.10e-22 | **MGRAPCCE** | **KLGLKKGRWTAEEDQVLTNYIQTHG** | **EGSWRSLPKN** |
| 25. | CsMYB35 | 9 | 1.70e-22 | **MGRQPCCD** | **KLGVKKGPWTAEEDKKLINFILTNG** | **HCCWRAVPKL** |
| 56. | CsMYB66 | 9 | 1.96e-22 | **MGRTPCCN** | **VQGLKKGAWTTEEDQKLSAYITQHG** | **EGGWRSLPEK** |
| 33. | CsMYB43 | 9 | 3.00e-22 | **MGRAPCCD** | **KANVKKGPWSPEEDATLKAYIEQNG** | **TGGNWIALPQ** |
| 32. | CsMYB42 | 9 | 3.45e-22 | **MGRAPCCD** | **KANVKKGPWSVEEDAKLKSYIENHG** | **TGGNWIALPQ** |
| 55. | CsMYB65 | 9 | 4.55e-22 | **MVRAPFID** | **ENGVKRGAWSPEEDDKLRAYVERYG** | **HWNWRELPKF** |
| 52. | CsMYB62 | 9 | 7.85e-22 | **MGRHSCCL** | **KQKLRKGLWSPEEDEKLFNYITHFG** | **VGCWSSVPKL** |
| 26. | CsMYB36 | 10 | 8.98e-22 | **MAMKSRLCE** | **KTHLKKGVWSPEEDQKLRSYIKKYG** | **IWNWTEMSKA** |
| 17. | CsMYB27 | 9 | 1.34e-21 | **MGRTPCCD** | **KKGLKKGPWTAEEDETLVEYIKKNN** | **GHGSWRSLPK** |
| 6. | CsMYB16 | 2 | 1.74e-21 | **M** | **VDRLKRGPWKPEEDKKLLAYIQQHG** | **HGSWRLVPKK** |
| 28. | CsMYB38 | 9 | 2.56e-21 | **MVRTPCCD** | **QNGMKKGTWTPEEDRKLVAYVTRYG** | **CWNWRQLPKF** |
| 62. | CsMYB72 | 29 | 4.81e-21 | **SGGKCVSGPD** | **GAILKKGPWTAAEDHVLMEYVKKHG** | **EGNWNSVQKY** |
| 58. | CsMYB68 | 29 | 4.81e-21 | **SGGKCVSGPD** | **GAILKKGPWTAAEDHVLMEYVKKHG** | **EGNWNSVQKY** |
| 76. | CsMYB86 | 15 | 5.44e-21 | **TRTCSGNSSE** | **EVEVRKGPWTMEEDLILINYIANHG** | **EGVWNSLAKA** |
| 12. | CsMYB22 | 9 | 1.00e-20 | **MGRSPCCE** | **KEHTNKGAWTKEEDQRLIHYIKLHG** | **EGCWRSLPKA** |
| 11. | CsMYB21 | 9 | 1.00e-20 | **MGRSPCCE** | **KEHTNKGAWTKEEDQRLIHYIKLHG** | **EGCWRSLPKA** |
| 53. | CsMYB63 | 20 | 1.27e-20 | **ANNTSSSEDD** | **GNELRRGPWTVEEDTLLIHYITRHG** | **EGRWNLLAKR** |
| 40. | CsMYB50 | 34 | 1.62e-20 | **EEDGPGGSRG** | **GAGLKKGPWTTSEDAILVEYVRKHG** | **EGNWNAVQKN** |
| 66. | CsMYB76 | 9 | 2.05e-20 | **MGRSPCCS** | **KKGLNKGAWTVLEDQILTDYIKTHG** | **EGKWRNLPKQ** |
| 64. | CsMYB74 | 34 | 2.58e-20 | **EKMGRIPCCE** | **KENVKRGQWTPEEDNKLSSYIAQHG** | **TRNWRLIPKN** |
| 46. | CsMYB56 | 21 | 3.26e-20 | **GGSKTTPCCS** | **KVGIKRGPWTAEEDEVLCEFIRKEG** | **EGRWRTLPKR** |
| 10. | CsMYB20 | 15 | 3.26e-20 | **MSWGMTNQDQ** | **QQGWRKGPWTPEEDKMLTEYVSFNG** | **EGRWSSVARS** |
| 69. | CsMYB79 | 8 | 3.65e-20 | **MGHRCCN** | **KLKVKRGLWSPEEDEKLLTHITTHG** | **HGNWSSVPKL** |
| 74. | CsMYB84 | 9 | 5.13e-20 | **MGRQPCCD** | **KIGLKRGPWTIEEDHKLMNFILNNG** | **IHCWRMVPKL** |
| 36. | CsMYB46 | 17 | 5.13e-20 | **AGHMGWGIIE** | **EEGWRKGPWTSEEDRLLIEYVRLHG** | **EGRWNSVARL** |
| 49. | CsMYB59 | 8 | 5.75e-20 | **MRKPCCE** | **KQDKNKGAWSIEEDQKLIDYINQHG** | **EGCWRTLPKA** |
| 63. | CsMYB73 | 37 | 7.19e-20 | **NDTNNNKMIK** | **NVKLRKGLWSPEEDEKLMRYMLRNG** | **QGCWSDIARN** |
| 48. | CsMYB58 | 6 | 1.25e-19 | **MVGNR** | **EEGIRKGPWKAEEDEVLINHVNKYG** | **PRDWSSIRSK** |
| 47. | CsMYB57 | 8 | 1.39e-19 | **MRKPCCD** | **KDGTNKGAWSKHEDQKLIDYITTHG** | **EGCWRSLPKA** |
| 57. | CsMYB67 | 9 | 1.73e-19 | **MGRTACCS** | **RFGLRRGPWTPREDTLLVNYIQLHG** | **EGHWRIVPKK** |
| 38. | CsMYB48 | 9 | 1.73e-19 | **MGRSPCCE** | **KAHTNKGAWTKEEDDRLIAYIRAHG** | **EGCWRSLPKA** |
| 82. | CsMYB92 | 13 | 1.92e-19 | **TITTKFNNEE** | **EIELRRGPWTLEEDTLLIHYISLHG** | **EGHWNLLAKR** |
| 81. | CsMYB91 | 13 | 1.92e-19 | **TITTKFNNEE** | **EIELRRGPWTLEEDTLLIHYISLHG** | **EGHWNLLAKR** |
| 70. | CsMYB80 | 9 | 1.92e-19 | **MGRSPCCS** | **KEGLNRGAWTAMEDRILSEYIKVHG** | **EGKWRSLPKR** |
| 27. | CsMYB37 | 9 | 2.38e-19 | **MGRAPCCE** | **KIGLKKGRWTSEEDEILTKYIQSNG** | **EGSWRSLPKN** |
| 37. | CsMYB47 | 9 | 4.03e-19 | **MGRAPCCD** | **KTKVKRGPWSPEEDAALKHYMHNNG** | **TGGNWIALPH** |
| 24. | CsMYB34 | 11 | 4.96e-19 | **MGRGRAPCCD** | **KSQVKRGPWSPAEDLRLITFIQKNG** | **HENWRALPKQ** |
| 84. | CsMYB94 | 50 | 5.49e-19 | **DEQDHDQEMN** | **NSDLRRGPWTVEEDLTLINYIAIHG** | **EGRWNSLARC** |
| 42. | CsMYB52 | 9 | 5.49e-19 | **MGRSPCCE** | **KDGLNRGAWSALEDKILADYIHKNG** | **PGKWRDLPKR** |
| 41. | CsMYB51 | 9 | 5.49e-19 | **MGRSPCCE** | **KDGLNRGAWSALEDKILADYIHKNG** | **PGKWRDLPKR** |
| 30. | CsMYB40 | 7 | 5.49e-19 | **MKRGSD** | **GEYIRKGPWRAEEDEVLINHVKKNG** | **PRDWSSIRSK** |
| 29. | CsMYB39 | 10 | 6.09e-19 | **MGPKKEGSS** | **KKVMNRGAWTAEEDKILSQYIKLHG** | **PRRWKTLSIQ** |
| 67. | CsMYB77 | 34 | 7.47e-19 | **LHSSEEDHHR** | **QLDLRKGPWTVEEDNMLFNYVSIHG** | **EGRWNSLARH** |
| 34. | CsMYB44 | 10 | 8.27e-19 | **MGCKSQDKP** | **KPKHRKGLWSPEEDLRLRNFVLKHG** | **HGCWSSVPIK** |
| 83. | CsMYB93 | 21 | 1.01e-18 | **SNNNNNNTNN** | **NNKLRKGLWSPEEDDKLMNYMLNNG** | **QGCWSDVARN** |
| 73. | CsMYB83 | 9 | 2.03e-18 | **MGRPPCCD** | **KSNVKRGLWTAEEDAKLLAHVSKLG** | **IGNWTLVPKK** |
| 61. | CsMYB71 | 56 | 2.47e-18 | **GSGDGDGDGG** | **GDGRVKGPWSPEEDVVLSRLVSKFG** | **ARNWSLIARG** |
| 59. | CsMYB69 | 52 | 2.47e-18 | **GAGSGDGDGG** | **GDGRVKGPWSPEEDVVLSRLVSKFG** | **ARNWSLIARG** |
| 80. | CsMYB90 | 8 | 5.87e-18 | **MATITRK** | **EMDRIKGPWSPEEDDSLQKLVEKHG** | **PRNWSLISKS** |
| 79. | CsMYB89 | 8 | 5.87e-18 | **MATITRK** | **EMDRIKGPWSPEEDDSLQKLVEKHG** | **PRNWSLISKS** |
| 71. | CsMYB81 | 9 | 8.54e-18 | **MVRPSSCE** | **KANLKGGLWSEEEDARMLEYVSKHG** | **KAKWTSVPKG** |
| 77. | CsMYB87 | 10 | 1.63e-17 | **MVRVLVKKS** | **SLNVKKGAWSREEDIVLRECIDKYG** | **EGKWHLVPLR** |
| 54. | CsMYB64 | 28 | 2.14e-17 | **SKPIEEENNF** | **NFKFNRGAWTAEEDQKLAEVISHHG** | **AQKWKTVAAI** |
| 72. | CsMYB82 | 24 | 2.81e-17 | **TDQREGGSSG** | **GRAVRKGAWTREEDDLLRDCVDKYG** | **EGKWHLVPLR** |
| 35. | CsMYB45 | 9 | 2.81e-17 | **MMENKRVK** | **KQLPKKNLWKPEEDLILKNYVETHG** | **EGNWQTVSKL** |
| 85. | CsMYB95 | 36 | 4.38e-17 | **GNNKVPRNGN** | **KPERIKGPWSTEEDRILTRLVDRYG** | **ARNWSLISRY** |
| 45. | CsMYB55 | 17 | 1.35e-16 | **TKTTRAMQGE** | **HDQLRKGTWLEEEDERLISFVQLMG** | **EKRWDALAKA** |
| 44. | CsMYB54 | 5 | 1.35e-16 | **MQGE** | **HDQLRKGTWLEEEDERLISFVQLMG** | **EKRWDALAKA** |
| 75. | CsMYB85 | 5 | 5.59e-16 | **MESL** | **LSRKVKGPWSPEEDEKLQSLVKQHS** | **ARNWSVISKS** |
| 78. | CsMYB88 | 3 | 5.07e-15 | **MK** | **GGDRIKGSWSPQEDETLKKLVEQHG** | **PRNWSVISTG** |
| 88. | CsMYB98 | 18 | 1.00e-14 | **SMSSDHGGGV** | **GKNCYRGHWRPGEDEKLRELVDRYG** | **PQNWNFIAEH** |

Motif 4 Purple boxes E-value: 2.6e-246 Site Count: 49 Width: 8


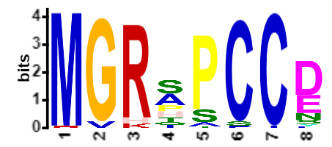


| 37. | CsMYB47 | 1 | 2.74e-12 |  | **MGRAPCCD** | **KTKVKRGPWS** |
| --- | --- | --- | --- | --- | --- | --- |
| 33. | CsMYB43 | 1 | 2.74e-12 |  | **MGRAPCCD** | **KANVKKGPWS** |
| 32. | CsMYB42 | 1 | 2.74e-12 |  | **MGRAPCCD** | **KANVKKGPWS** |
| 31. | CsMYB41 | 1 | 2.74e-12 |  | **MGRAPCCD** | **KANVKKGPWS** |
| 20. | CsMYB30 | 1 | 2.74e-12 |  | **MGRAPCCD** | **KNGLKKGPWT** |
| 18. | CsMYB28 | 1 | 2.74e-12 |  | **MGRAPCCD** | **KNGLKKGPWT** |
| 51. | CsMYB61 | 1 | 7.95e-12 |  | **MGRAPCCE** | **KMGLKKGPWT** |
| 50. | CsMYB60 | 1 | 7.95e-12 |  | **MGRAPCCE** | **KMGLKKGPWT** |
| 43. | CsMYB53 | 1 | 7.95e-12 |  | **MGRAPCCE** | **KLGLKKGRWT** |
| 27. | CsMYB37 | 1 | 7.95e-12 |  | **MGRAPCCE** | **KIGLKKGRWT** |
| 21. | CsMYB31 | 1 | 1.49e-11 |  | **MGRSPCCD** | **KANVKKGPWS** |
| 13. | CsMYB23 | 1 | 1.49e-11 |  | **MGRSPCCD** | **GINIGLKKGP** |
| 9. | CsMYB19 | 1 | 1.49e-11 |  | **MGRSPCCD** | **KVGLKKGPWT** |
| 5. | CsMYB15 | 1 | 1.49e-11 |  | **MGRSPCCD** | **ESGLKKGPWT** |
| 73. | CsMYB83 | 1 | 1.80e-11 |  | **MGRPPCCD** | **KSNVKRGLWT** |
| 4. | CsMYB14 | 1 | 1.80e-11 |  | **MGRPPCCD** | **KEGVKKGPWT** |
| 3. | CsMYB13 | 1 | 1.80e-11 |  | **MGRPPCCD** | **KEGVKKGPWT** |
| 2. | CsMYB12 | 1 | 1.80e-11 |  | **MGRPPCCD** | **KVGVKKGPWT** |
| 1. | CsMYB11 | 1 | 1.80e-11 |  | **MGRPPCCD** | **KVGIKKGPWT** |
| 65. | CsMYB75 | 1 | 2.48e-11 |  | **MGRTPCCD** | **QKGLRKGAWT** |
| 17. | CsMYB27 | 1 | 2.48e-11 |  | **MGRTPCCD** | **KKGLKKGPWT** |
| 74. | CsMYB84 | 1 | 3.42e-11 |  | **MGRQPCCD** | **KIGLKRGPWT** |
| 42. | CsMYB52 | 1 | 3.42e-11 |  | **MGRSPCCE** | **KDGLNRGAWS** |
| 41. | CsMYB51 | 1 | 3.42e-11 |  | **MGRSPCCE** | **KDGLNRGAWS** |
| 38. | CsMYB48 | 1 | 3.42e-11 |  | **MGRSPCCE** | **KAHTNKGAWT** |
| 25. | CsMYB35 | 1 | 3.42e-11 |  | **MGRQPCCD** | **KLGVKKGPWT** |
| 16. | CsMYB26 | 1 | 3.42e-11 |  | **MGRQPCCD** | **KVGLKKGPWT** |
| 12. | CsMYB22 | 1 | 3.42e-11 |  | **MGRSPCCE** | **KEHTNKGAWT** |
| 11. | CsMYB21 | 1 | 3.42e-11 |  | **MGRSPCCE** | **KEHTNKGAWT** |
| 7. | CsMYB17 | 1 | 3.42e-11 |  | **MGRSPCCE** | **KVGLKKGPWT** |
| 64. | CsMYB74 | 26 | 5.60e-11 | **VDQASCGLEK** | **MGRIPCCE** | **KENVKRGQWT** |
| 39. | CsMYB49 | 1 | 9.16e-11 |  | **MGRSPCCN** | **VQGLKKGAWT** |
| 56. | CsMYB66 | 1 | 1.38e-10 |  | **MGRTPCCN** | **VQGLKKGAWT** |
| 70. | CsMYB80 | 1 | 1.75e-10 |  | **MGRSPCCS** | **KEGLNRGAWT** |
| 66. | CsMYB76 | 1 | 1.75e-10 |  | **MGRSPCCS** | **KKGLNKGAWT** |
| 28. | CsMYB38 | 1 | 2.10e-10 |  | **MVRTPCCD** | **QNGMKKGTWT** |
| 87. | CsMYB97 | 1 | 5.61e-10 |  | **MGRHSCCY** | **KQKLRKGLWS** |
| 86. | CsMYB96 | 1 | 5.61e-10 |  | **MGRHSCCY** | **KQKLRKGLWS** |
| 24. | CsMYB34 | 3 | 7.74e-10 | **MG** | **RGRAPCCD** | **KSQVKRGPWS** |
| 57. | CsMYB67 | 1 | 2.39e-9 |  | **MGRTACCS** | **RFGLRRGPWT** |
| 52. | CsMYB62 | 1 | 2.90e-9 |  | **MGRHSCCL** | **KQKLRKGLWS** |
| 8. | CsMYB18 | 1 | 4.07e-9 |  | **MGRSPSCD** | **ENGLKKGPWT** |
| 14. | CsMYB24 | 1 | 5.69e-9 |  | **MGKIACCD** | **KNGVKKGAWT** |
| 68. | CsMYB78 | 1 | 1.15e-8 |  | **MGHHSCCN** | **KQKVKRGLWS** |
| 23. | CsMYB33 | 1 | 1.15e-8 |  | **MGHHSCCN** | **QQKVKRGLWS** |
| 22. | CsMYB32 | 1 | 1.15e-8 |  | **MGHHSCCN** | **QQKVKRGLWS** |
| 19. | CSMYB29 | 1 | 2.31e-8 |  | **MGKITCCE** | **KNGLKKGPWT** |
| 71. | CsMYB81 | 1 | 2.53e-7 |  | **MVRPSSCE** | **KANLKGGLWS** |
| 55. | CsMYB65 | 1 | 6.28e-7 |  | **MVRAPFID** | **ENGVKRGAWS** |

Motif 5 Orange boxes E-value: 5.3e-109 Site Count: 26 Width: 12


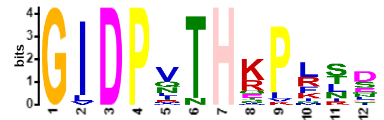


| 8. | CsMYB18 | 120 | 6.46e-15 | **THLKKKLLQM** | **GIDPVTHRPRTD** | **IHLNLLSNLP** |
| --- | --- | --- | --- | --- | --- | --- |
| 71. | CsMYB81 | 120 | 1.34e-14 | **TKLRKKLSEM** | **GIDPVTHKPFSQ** | **IIADYGNVGG** |
| 64. | CsMYB74 | 145 | 1.77e-14 | **TKLKKKLSGM** | **GIDPVTHKPFSH** | **LMAEIATTLE** |
| 13. | CsMYB23 | 123 | 3.59e-14 | **TYLRKKLLQM** | **GIDPQTHKPRTD** | **LNHFLNISHQ** |
| 7. | CsMYB17 | 120 | 5.72e-14 | **THLKKRLTKM** | **GIDPVTHKPKID** | **ALGSGSGNPK** |
| 73. | CsMYB83 | 120 | 1.46e-13 | **TKLRKKLYTM** | **GIDPVTHKPISQ** | **ILSEFGNISG** |
| 6. | CsMYB16 | 113 | 1.99e-13 | **THLKKRLTTM** | **GIDPVTHKPLKH** | **TLRSVAADPK** |
| 18. | CsMYB28 | 120 | 2.32e-13 | **THIRKRLLRM** | **GIDPVTHAPRLD** | **FLDLSSILAA** |
| 5. | CsMYB15 | 120 | 2.32e-13 | **THLKKKLIQM** | **GIDPMTHRPRTD** | **IFSSLPHLIA** |
| 19. | CSMYB29 | 120 | 5.73e-13 | **THIRKRLLKM** | **GIDPVTHTPRLD** | **LLELSSLLNS** |
| 87. | CsMYB97 | 120 | 7.69e-13 | **SCIKKKLRQK** | **GIDPNTHKPLSE** | **VENDIGNKLE** |
| 86. | CsMYB96 | 120 | 7.69e-13 | **SCIKKKLRQK** | **GIDPNTHKPLSE** | **VENDIGNKLE** |
| 20. | CsMYB30 | 120 | 9.98e-13 | **THIRKRLLRM** | **GIDPVTHSPRLD** | **LLDLSSILSS** |
| 9. | CsMYB19 | 120 | 1.13e-12 | **THLKKRLAKM** | **GIDPITHKPKND** | **NLLSQQDGGQ** |
| 12. | CsMYB22 | 120 | 2.47e-12 | **THIKRKLYSR** | **GIDPQTHRPLLL** | **MSTTTTNNKT** |
| 11. | CsMYB21 | 120 | 2.47e-12 | **THIKRKLYSR** | **GIDPQTHRPLLL** | **MSTTTTNSSS** |
| 25. | CsMYB35 | 120 | 3.89e-12 | **THIKKKLLKM** | **GIDPVTHEPLII** | **INKDNDQLNK** |
| 38. | CsMYB48 | 120 | 4.33e-12 | **THIRRKLLNR** | **GIDPATHRPLNE** | **SGQETTNTST** |
| 52. | CsMYB62 | 120 | 6.75e-12 | **SCLKKKLVKQ** | **GIDPTTHKPLSN** | **ENNNNNNTME** |
| 46. | CsMYB56 | 132 | 7.52e-12 | **THLSKKLINQ** | **GIDPRTHKPFNN** | **NNNINNNQTP** |
| 16. | CsMYB26 | 120 | 8.34e-12 | **THIKKKLKKM** | **GIDPLTHQPLSS** | **TVTDQYSHDD** |
| 17. | CsMYB27 | 121 | 1.98e-10 | **THLKKRLLCM** | **GLDPLTHEPFNS** | **NSHYPPTASS** |
| 74. | CsMYB84 | 120 | 1.37e-9 | **TRIKKRLKVL** | **GVDPITHQKISS** | **SSETTTTTTT** |
| 49. | CsMYB59 | 119 | 2.61e-9 | **SHLRRKLINM** | **GIDPNNHRLKTT** | **TNTTSPLLSF** |
| 69. | CsMYB79 | 119 | 3.71e-9 | **SCIKKKLMSQ** | **GLDPQTHNLMTS** | **SSHHQNKSQN** |
| 47. | CsMYB57 | 119 | 5.60e-9 | **SHLRKKLINL** | **GIDPNNHKVNRS** | **QNHDHSATTT** |

**Figure S1.** **Amino acid sequence conservation of R1R2R3-type (a), R2R2-type (b) and R3-type (c) CsMYB domain**. The sequence logos of each R MYB repeat are based on full-length alignments of each class of CsMYBs (for full sequences see Supplementary File S2). The overall height of each stack indicates the conservation of the sequence at each position, while letters height whithin each stack shows the relative frequency of the corresponding amino acid.

**A**


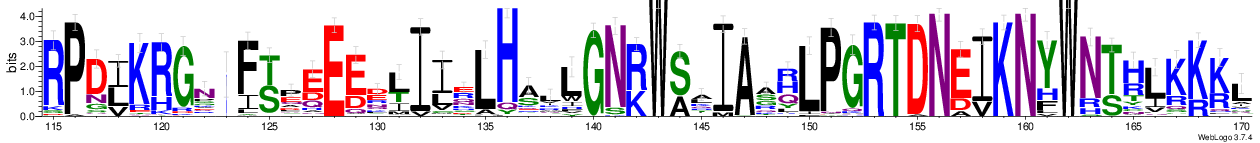

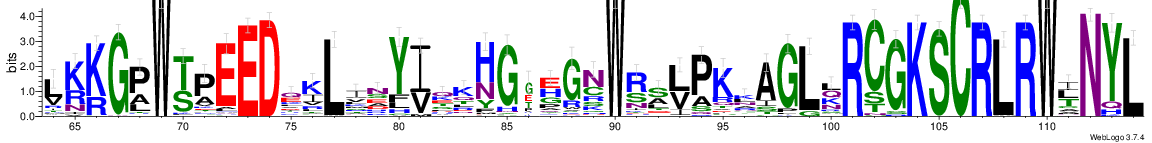

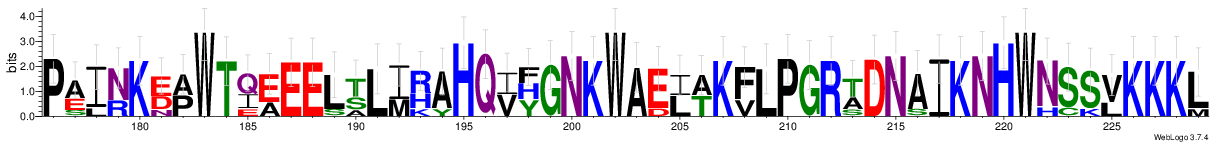

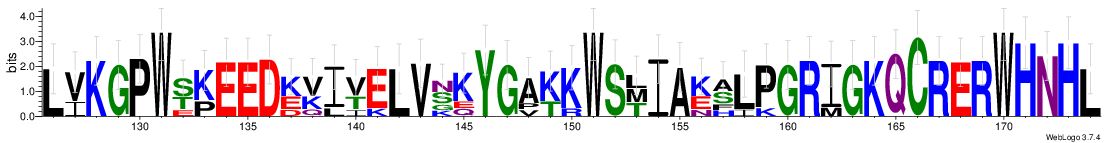

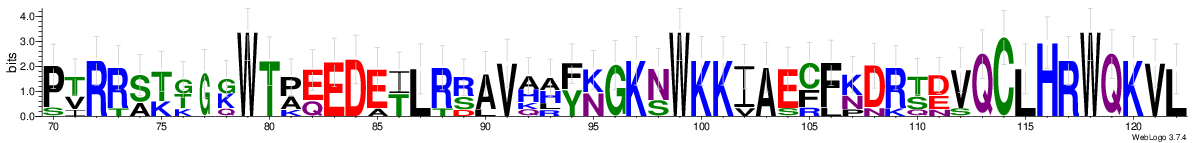


**B**

**
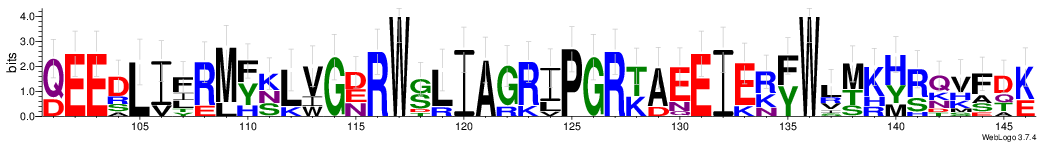
C**
